# *In vitro* antiplasmodial activity of cepharanthine

**DOI:** 10.1186/1475-2875-13-327

**Published:** 2014-08-22

**Authors:** Camille Desgrouas, Charles Chapus, Jérôme Desplans, Christelle Travaille, Aurélie Pascual, Béatrice Baghdikian, Evelyne Ollivier, Daniel Parzy, Nicolas Taudon

**Affiliations:** UMR-MD3, Institut de recherche biomédicale des armées, Faculté de Pharmacie, Aix-Marseille Université, 27 Bd Jean Moulin CS30064 13385 Marseille cedex 5, Marseille, France; UMR-MD3, Institut de recherche biomédicale des armées, BP73 91223 Brétigny-sur-Orge, France; Trypanosome Cell Biology Unit, CNRS URA2581 and Parasitology Department, Institut Pasteur, 25 rue du Docteur Roux, 75015 Paris, France; Département d’Infectiologie de Terrain, Unité de Parasitologie, Institut de Recherche Biomédicale des Armées, Marseille, France; UMR-MD3, Laboratoire de Pharmacognosie et Ethnopharmacologie, Faculté de Pharmacie, Aix-Marseille Université, 27 Bd Jean Moulin 13385 Marseille Cedex 5, Marseille, France

**Keywords:** *Stephania rotunda*, Cepharanthine, *Plasmodium falciparum*, Antiplasmodial activity, Transcriptomic analysis

## Abstract

**Background:**

New classes of anti-malarial drugs are needed to control the alarming *Plasmodium falciparum* resistance toward current anti-malarial therapy. The ethnopharmacological approach allows the discovery of original chemical structures from the vegetable biodiversity. Previous studies led to the selection of a bisbenzylisoquinoline, called cepharanthine and isolated from a Cambodian plant: *Stephania rotunda*. Cepharanthine could exert a mechanism of action different from commonly used drugs. Potential plasmodial targets are reported here.

**Methods:**

To study the mechanism of action of cepharanthine, a combined approach using phenotypic and transcriptomic techniques was undertaken.

**Results:**

Cepharanthine blocked *P. falciparum* development in ring stage. On a culture of synchronized ring stage, the comparisons of expression profiles showed that the samples treated with 5 μM of cepharanthine (IC_90_) were significantly closer to the initial controls than to the final ones. After a two-way ANOVA (p-value < 0.05) on the microarray results, 1,141 probes among 9,722 presented a significant differential expression.

A gene ontology analysis showed that the Maurer’s clefts seem particularly down-regulated by cepharanthine. The analysis of metabolic pathways showed an impact on cell-cell interactions (cytoadherence and rosetting), glycolysis and isoprenoid pathways. Organellar functions, more particularly constituted by apicoplast and mitochondrion, are targeted too.

**Conclusion:**

The blockage at the ring stage by cepharanthine is described for the first time. Transcriptomic approach confirmed that cepharanthine might have a potential innovative antiplasmodial mechanism of action. Thus, cepharanthine might play an ongoing role in the progress on anti-malarial drug discovery efforts.

**Electronic supplementary material:**

The online version of this article (doi:10.1186/1475-2875-13-327) contains supplementary material, which is available to authorized users.

## Background

Malaria remains a major public health problem which affected about 207 million people and caused an estimated 627,000 deaths in 2012 [1]. In the context of the widespread and increasing occurrence of *Plasmodium falciparum* resistance against current anti-malarial therapy, new anti-malarial compounds are urgently needed to treat this major endemic disease. In this perspective, it is interesting to note that for many synthetic anti-malarial drugs, *P. falciparum*-resistant isolates were observed one to 12 years after the first use, whereas it was longer for the natural compounds [2]. Indeed, the first reported resistance towards quinine appeared 278 years after its introduction [3]. The use of artemisinin combination therapy (ACT) as first-line treatment of uncomplicated malaria caused by *P. falciparum* was officially recommended by the WHO in 2006 [4]. Unfortunately, 2,000 years after the use of *Artemisia annua* in the Chinese Pharmacopoeia to treat fever, the emergence of resistance to artemisinin derivatives was recently reported from Southeast Asia [5, 6]. Molecules, structurally different from the available anti-malarial drugs and targeting innovative and independent metabolism pathways, are particularly needed to prevent the apparition of resistance and to improve care. Drawing from the rich plant biodiversity, new chemical structures may be helpful in the fight against malaria [7].

The ethnopharmacology, based on traditional medicine, offers interesting possibilities in the discovery of new bioactive compounds isolated from the nature. A collaboration between the Cambodian and French (UMR-MD3) faculties allowed inquiries on 28 Cambodian plants used in traditional medicine [8]. This work allowed the selection of *Stephania rotunda*, *Brucea javanica*, *Phyllanthus urinaria* and *Eurycoma longifolia,* among which, *S. rotunda* (Menispermaceae), a creeping plant growing on calcareous cliffs of Cambodian mountain areas [9], exhibited the most interesting antiplasmodial activity *in vitro*. Concentrations inhibiting 50% of parasitic growth (IC_50_) of the dichloromethane and water extracts of *S. rotunda* tuber were below 5 μg/ml on the *Plasmodium* strain W2 [8]. The fractionation of dichloromethane extracts allowed the isolation of nine alkaloids. The main compound is a bisbenzylisoquinoline, named cepharanthine. This alkaloid has recently been extracted by green chemistry using ultrasound and microwave technologies [10].

Possessing an interesting IC_50_ measured by flow cytometry (0.61 μM on W2 strain), the antiplasmodial activity of cepharanthine was tested in mice infected by *Plasmodium berghei* at a dose of 10 mg/kg [8]. By intraperitoneal injection and oral administration, this alkaloid decreased the parasitaemia by 47 and 50%, respectively. Despite the absence of mice sterilization, this molecule is interesting in combination with other anti-malarial drugs. Indeed, cepharanthine possesses a synergistic activity with chloroquine [8, 11] but the mechanism of this potentiation is not known currently. Two hypotheses have been proposed to explain this phenomenon: an alteration of the parasite membrane potential [12] or a modulation of P-glycoprotein [13] by cepharanthine.

Previous work showed that cepharanthine seemed to possess a putative mechanism of action different from those of anti-malarial drugs commonly used. Indeed, cepharanthine did not affect the crystallization of haem, unlike chloroquine. The measurement of mitochondrial membrane depolarization after labelling with DiOC6 did not show any effect on the mitochondrial membrane potential by cepharanthine, contrary to atovaquone. The use of ascorbic acid as a potential inhibitor of free radical production did not reveal any activity of free radicals production for cepharanthine, contrary to artemisinin and its derivatives [14].

The work presented here highlights potential plasmodial targets of cepharanthine using both phenotypic and transcriptional approaches.

## Methods

### Drug sensitivity assay

Chloroquine (CQ) and mefloquine (MQ) drugs were purchased from Sigma (St Louis, MO, USA). CQ-resistant/MQ-susceptible clones FCM2 (Cameroon), W2 (Vietnam), K1 (Thailand), and CQ-susceptible/MQ- resistant 3D7 strain (from NF54 African strain, MR4: Malaria Research and Reference Reagent Resource centre) were used in this study. Parasites were cultivated in type A^+^ human erythrocytes (2% haematocrit) suspended in RPMI 1640 medium (Invitrogen, Paisley, UK) supplemented with 10% human serum (Abcys SA, Paris, France) and buffered with 25 mM HEPES-25 mM NaHCO_3_ under controlled atmospheric conditions (10% O_2_, 5% CO_2_, and 85% N_2_) at 37°C with 95% humidity. Cultures were synchronized at the ring stage by two successive D-sorbitol 5% (m/v) (Sigma-Aldrich) treatments, which were applied with an interval of four hours; the first to old schizonts in the process of releasing the merozoites and the second to rings obtained from the merozoites release to kill mature schizonts still in the culture [15]. The *in vitro* assay has been performed once, in triplicate, on all strains simultaneously. Then the IC_50_ values obtained for positive controls (MQ and CQ) allow the validation of the results by comparison with bibliographic references.

Cepharanthine (>99%) was extracted from the *S. rotunda* tuber, according to a percolation method previously validated in the Laboratory of Pharmacognosy and Ethnopharmacology of Aix-Marseille University [16]. The culture of *P. falciparum* strains was performed with the method of Trager and Jensen [17]. Cepharanthine IC_50_ levels were determinate on the four plasmodial strains, according to Desjardins method [18], using tritied hypoxanthine with a specific activity of 5 mCi/5 mL (Perkin-Elmer, Courtaboeuf, France) to evaluate the parasitic growth. Stock solutions of the test substances were prepared in 5% DMSO and 95% methanol with a final concentration of 395.6 μM. CQ and MQ were used as references. Cepharanthine, CQ and MQ activities were evaluated as a ten serial two-fold dilutions of, respectively, 19.53-20,000, 3.9-5,000 and 0.39-400 nM. The dilutions of each compound were set in a 96-well plate, in triplicate, and dried overnight. Test solutions were then mixed with a suspension of infected human red blood cells to achieve a final haematocrit of 1.5% and parasitaemia of 0.8%, and incubated for 48 hr in microtitre plates (200 μL final volume). IC_50_ levels, i e, the drug concentration corresponding to 50% of the uptake of [^3^H] hypoxanthine by the parasites in drug-free control wells, was determined by non-linear regression analysis of log dose response curves (Riasmart, Packard, Meriden, NJ, USA).

### Morphologic characterization of cepharanthine effects

As described above, 45 hours after a tight synchronization, the parasites were used to study the effect of cepharanthine. The parasite morphology and parasitaemia were evaluated by microscopic observation (×100) of thin blood smears stained with RAL^®^ 555 (REACTIFS RAL, Martillac, France).

In order to design the transcriptional assay, three studies were performed on different *P. falciparum* cultures with the 3D7 strain. Parasites were incubated with cepharanthine at the concentration corresponding to the IC_50_ level. Blood smears were frequently prepared to monitor the development of parasite life cycle stages. In a first step, cepharanthine was incubated in a continuous fashion on each erythrocyte stage (ring, trophozoite and schizont) until the third merozoite invasion in the control group, for investigation of a dependent-stage effect. Then, cepharanthine was incubated on ring stage and during the equivalent of one *P. falciparum* life cycle (45 hr). After this treatment, cultures were washed three times and blood smears were prepared during a 90-hr period to follow-up a potential parasitic recrudescence. In a last experiment, cepharanthine was added continuously during 144 hr on rings aged of 4 hr, 10 hr or 16 hr. The aim of this assay was to ascertain a potential effect depending on the time of cepharanthine incubation after the merozoite invasion.

### Transcriptional analysis

#### Parasitic treatment and RNA extraction

**Figure 1:**
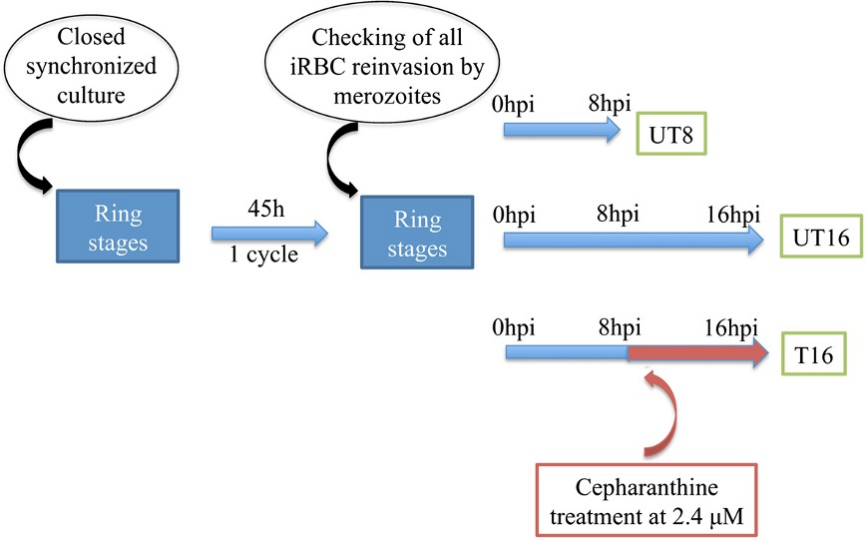
**Design of the transcriptional assay.** UT8 = untreated samples 8 hr post-invasion by merozoite (h pmi); UT16 = untreated samples 16 h pmi; T16 = samples treated by cepharanthine at 8 h pmi during eight hours; iRBC = infected red blood cells.

Cultures of *P. falciparum* 3D7 strain were closely synchronized in ring stages by two sequential D-sorbitol 5% treatments. One life cycle later, re-invasion and synchronization were checked on thin blood smears. Cultures were pooled and divided into 12 samples to constitute four biological replicates for each the three analytic groups: UT8 and UT16 were the controls 8 hr and 16 hr post-merozoite invasion (h pmi), respectively; T16 was the group exposed to a 8-hr period of cepharanthine incubation started at 8 hr pmi (Figure 1). Cepharanthine was used at the concentration corresponding to the IC_90_ estimated graphically from the curve modeled for IC_50_ calculation on the 3D7 strain. As the duration of the ring stage period was 20 hr pmi, this time-window prevented invasion events and enabled a selective assay. RNA was extracted from erythrocyte pellets blocked by TRIZOL™ reagent according to manufacturer’s recommendations (Invitrogen, Carlsbad, CA, USA), and treated with DNase (DNAfree™, Ambion, Foster City, CA, USA). RNA samples were checked for integrity using the NanoDrop ND-1000 (Labtech, Palaiseau, France) and qualified using a 2100 Bioanalyzer RNA Nano Chip (Agilent Technologies, Santa Clara, CA, USA) according to the manufacturer’s protocol. No trace of DNA contamination was detected by Bioanalyzer analysis.

### Microarray assay and analysis

A reference RNA was obtained pooling an equal amount of RNA of each sample (UT8, UT16 and T16) to normalize the data arising from the microarray experiment [19]. Microarrays were performed using the Agilent protocol version 6.5 (Low Input Quick Amp Labeling). Cyanine-3 CTP (Cy-3) fluorescent dye was used to label the samples and Cy-5 fluorescent dye to label the reference (RNA pool). The labelled cRNA were hybridized on the *P. falciparum* 4x44k Agilent custom microarrays developed in the parasitology laboratory of Army Biomedical Research Institute (Marseille). On these microarrays, 9,722 probes were spotted, corresponding to 5,144 genes. Microarrays were scanned using an Agilent Scanner (G2505B). Data were extracted and normalized from the scanned images using the Agilent Feature Extraction software (ver 9.5.3.1). The annotation of the microarray was updated using the PlasmoDB 9.0 annotations [20] and the Malaria Parasite Metabolic Pathways [21]. The version 9.0 of PlasmoDB introduced a new name code for each *P. falciparum* gene and updated the Gene Ontology (GO) annotation. Two-way ANOVA analyses (time-treatment) were performed with a FDR correction and a p-value of 0.05 on normalized data using the Genespring GX software (ver 12.0). The significant, differentially expressed probes were filtered using a fold change (FC) above two between UT8, UT16 and T16. Genespring allowed the computation of a hierarchical clustering (Euclidian metric, centroid method) and GO analyses. The significantly over-represented GOs were input in QuickGO [22] in order to produce an Ancestral Chart, representing these GOs with their relationships.

The pathway analyses were performed using the statistical software R [23] and the Malaria Parasite Metabolic Pathways database [21] as reference. The over-representations of pathways were determined by Fisher exact t-tests with a p-value threshold of 0.05. Then, the significant pathways are grouped by functions according to the Malaria Parasite Metabolic Pathways database.

### Microarray validation by real-time quantitative polymerase chain reaction

The real-time quantitative polymerase chain reaction (qRT-PCR) was performed on an Applied Biosystems 7900 Fast Real-Time PCR system (Carlsbad, California, USA). Primers were designed using the Applied Biosystems software Primer Express (ver 2.0.0). In order to avoid genomic DNA contamination, the following rules have been used for the primers. The primers were designed close to the 3' end of the genes to take into account the reverse transcriptase step. If possible, we chose primers spanning intron. Due to the particular *P. falciparum* nucleotide composition, the size of the primers were selected between 18 and 35 nt, with a Tm between 56 and 62°C. The primers were previously tested at two different concentrations (0.5 and 0.9 μM) to select the most efficient one. For each primer, the used concentration was different (see Additional file 1). Efficiency was calculated by the following formula:



Efficiency must be high (superior to 0.95) and constant among samples.

One microgram of the DNase-treated total RNA was reverse-transcribed with the High-Capacity cDNA Archive kit (Applied Biosystems, Carlsbad, CA, USA). The expression of 12 genes was evaluated on each cDNA sample (see Additional file 1). PCR amplifications were carried out using 12.5 μL SYBR^®^Green PCR Master Mix (2X) (Power SYBR^®^Green, Applied Biosystems, Carlsbad, CA, USA), 2.5 μL of each primer and 5 μL of template DNA in a final volume of 25 μL. The thermal cycling conditions were 95°C for 10 min, and 40 cycles of 95°C for 15 sec, then 60°C for 60 sec. For each gene, a no template control was used (water). The fluorescence acquisition was performed at the end of each extension step. The measurements were performed in triplicate for all the samples and genes. The 2^-ΔΔCt^ formula was applied to normalize the detected fluorescent signal with endogenous reference *Plasmodium* ribosomal small subunit 18 s rRNA and to compare each sample with the controls.


## Results

### Drug sensitivity assay

The IC_50_ values of cepharanthine, CQ and MQ were respectively between 927 and 3,059 nM, 21.2 and 738 nM, 14.3 and 67.0 nM depending on the *P. falciparum* strain used (Table 1). The levels of activity obtained for CQ and MQ were in accordance with the results found in the literature [24, 25]. The levels of relative sensitivity of cepharanthine *vs* CQ and MQ were different regarding strains. IC_50_ ratios of cepharanthine/CQ and cepharanthine/MQ were between (1.6–107), and (34.0-126), respectively.

**Table 1:** ***IC***_***50***_ **of cepharanthine against four** ***Plasmodium falciparum*** **strains**

Compound/strain	FCM29	W2	3D7	K1
Cepharanthine	3059 (12.3%)	927 (6.5%)	2276 (15.8%)	1803 (17.5%)
Chloroquine (CQ)	738 (7.2%)	572 (19.5%)	21.2 (13.7%)	164 (35.1%)
Mefloquine (MQ)	24.5 (10.1%)	26.5 (9.2%)	67.0 (5.9%)	14.3 (28.0%)
IC_50_ ratio (Cepharanthine/CQ)	4.1	1.6	107	11.0
IC_50_ ratio (Cepharanthine/MQ)	125	35.0	34.0	126
IC_50_ ratio (CQ/MQ)	30.1	21.6	0.3	11.5

The first three rows are IC_50_ (arithmetic mean in nM, RSD%) of three drugs on four *Plasmodium* falciparum strains. These values have been computed using triplicates (n = 3). The last three rows are the IC_50_ ratio between every pair of the tested drugs.

### Morphologic characterization of cepharanthine effects

Morphological assays were performed on close synchronized cultures to highlight a potential effect of cepharanthine depending of the *Plasmodium* stage. Indeed, such referrals were required beforehand to design a relevant transcriptional assay. Parasites were incubated with cepharanthine at the concentration corresponding to the IC_50_ level on the 3D7 stain.

In a first step, cepharanthine was incubated in a continuous fashion on each erythrocyte stage (ring, trophozoite and schizont). Ring stages could not differentiate into trophozoites. While at trophozoite and schizont stages a decrease in parasitaemia was observed. The surviving parasites progressed in their life cycle until the next ring stage, and then differentiation was definitively blocked.

After incubation on ring stages during a 45-hr period, parasites resumed their growth according a normal life cycle about 48 hr after removal of the drug pressure. Thus, cepharanthine could have a parasitostatic rather than a parasitocidal effect.

Effect of cepharanthine inside the ring stage was investigated by incubating the drug at 4, 10 and 16 hr pmi during a 144-hr period. As described above, parasitical growth was blocked whatever the delay for incubation. The parasitaemia was not positive at the end of the observation period.

Based on all these morphological observations, a microarray approach targeting effect of cepharanthine on *Plasmodium* ring stage has been designed.

### Transcriptional assay

**Figure 2:**
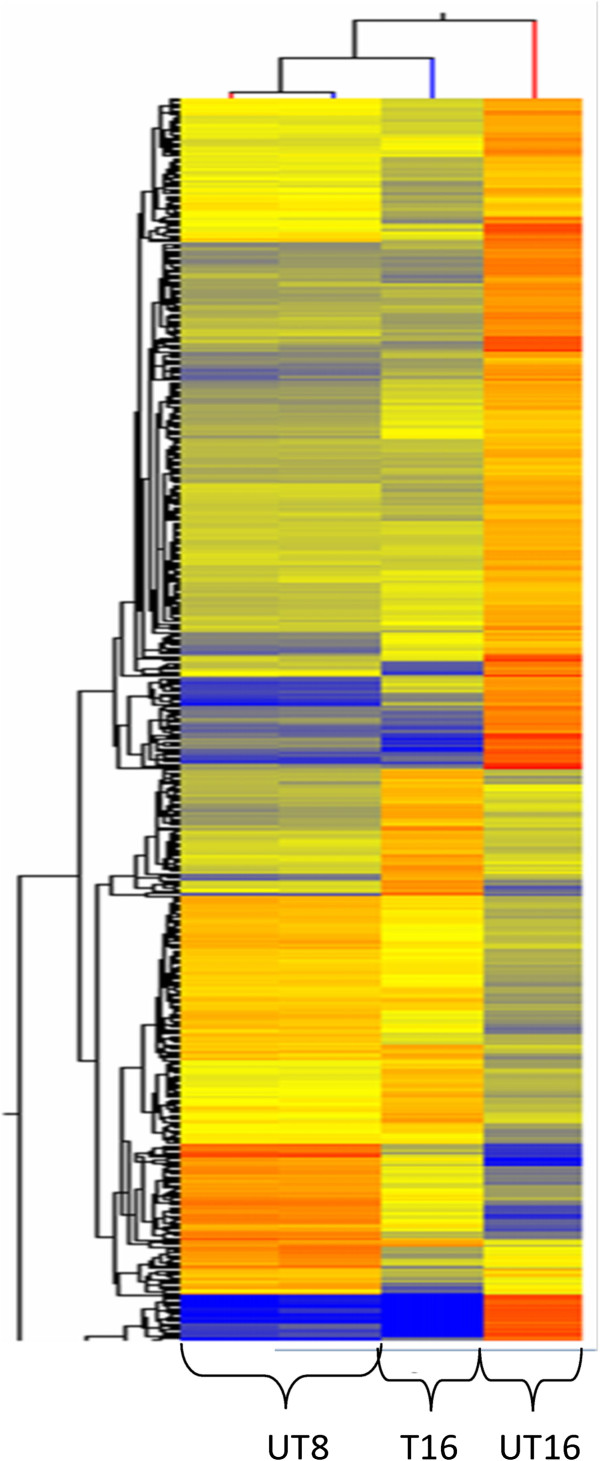
**Hierarchical clustering showing gene expression before (UT8), after (T16) and without treatment with cepharanthine (UT16).** The genes in blue are under-expressed, the genes in red are over-expressed, and the genes in yellow represent genes not affected by cepharanthine.

The use of microarrays allowed the study of cepharanthine impact on the entire *Plasmodium* transcriptome simultaneously and underlined the probable pathways impacted by this compound. The sampling times for the design of transcriptional analysis were determined as 8 and 16 h pmi and the concentration of cepharanthine was 5 μM. Applying a two-way ANOVA analysis and a FC threshold of two, 1,141 probes corresponding to 781 genes showed a significant variation of expression (see Additional file 2). In PlasmoDB 9.0, 44.7% of these probes have been annotated as unknown function. A hierarchical clustering showed a wide proximity between UT8 and T16 (Figure 2). The probes possessing the similar expression profiles were grouped in the same cluster. Two groups of probes were differentially expressed between UT8 and T16: probes under- (in blue) or over-expressed (in red) by cepharanthine. The comparison of probes expression between UT16 and T16 showed no similarity, indicating that the entire transcriptome was affected by the cepharanthine treatment.

The most important variations of expression were observed for six genes families (see Additional file 2). The FCs obtained from surface-associated, interspersed gene (*SURFIN*) were between -5 and +9 (UT16 *vs* T16 for PF13_0074 and UT8 *vs* UT16 for PFA0655w, respectively). For *STEVOR*, these values were ranged between -17 and +62 (UT8 *vs* UT16 and UT16 *vs* T16, respectively for PF10_0395). The *RIFIN* FCs were contained between -13 and +27 (UT8 *vs* UT16 and UT16 *vs* T16, respectively for PFD0055w). The FCs of protein kinases were between -4.5 and +5.5 (UT8 *vs* UT16 for, respectively MAL13P1.185 and PF14_0264). The FC values of *Plasmodium*-exported proteins (PHIST and hyp) were ranged between -19.5 and +11 (UT8 *vs* UT16 for, respectively PFA0700c and MAL8P1.160). The *P. falciparum* two-transmembrane Maurer’s cleft protein (*Pf*mc-2TM) FCs were between -16 and +24.6 (UT8 *vs* UT16 for PFA0065w and UT16 *vs* T16 for PF11_0014, respectively).

### Gene Ontology and pathways analysis

The GO analysis has been performed on the differentially expressed genes. Sixty GOs terms are significantly over-represented (Fisher exact t-test, p-value < 0. 05). These GOs involved particularly ‘cellular components’ with Maurer’s clefts and apicoplast; ‘biological process’ with antigenic variation, glyconeogenesis and mitochondria. Only three significant GOs, involving amine and lipid binding, were found in the ‘molecular function’ group (see Additional file 3).

**Figure 3:**
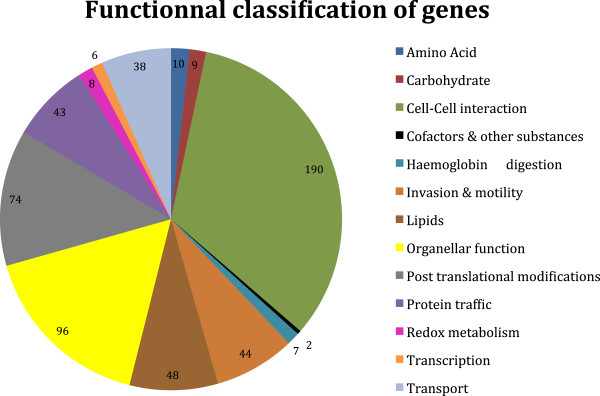
**Functional classification of genes with expression modified by cepharanthine.** The number of genes in each category is indicated in the outer circle (e.g. 21. Malaria Parasite Metabolic Pathways [http://priweb.cc.huji.ac.il/malaria/]).

Pathways analysis showed few differences between UT8 and T16. The most impacted function between these two experimental conditions seemed to be invasion and motility, containing two pathways under-expressed by cepharanthine (the functional annotation of merozoite invasion-related proteins and the subcellular localization of proteins involved in invasion) (see Additional file 4). A Fisher’s exact test right showed that 20 pathways were significantly modified by cepharanthine (p-value <0.1) (see Additional file 5). According to the functional classification of genes obtained (Figure 3), cyto-adherence and rosetting, included in cell-cell interaction group, are the most significant pathways (p-value = 2.49 × 10^-13^ and 1.54 × 10^-11^, respectively). Cyto-adherence corresponds to the capacity of parasitized erythrocytes to adhere to endothelial cells, whereas rosetting corresponds to the ability of parasitized erythrocytes to adhere to uninfected erythrocytes. These two properties are responsible for the sequestration of infected red blood cells, mainly in capillaries of the deep microvasculature. The organellar functions, including mitochondrion and apicoplast, are significant pathways in this analysis. These two organelles were also cited by the GO analysis and are important for the parasite [26]. The histograms confirmed that a treatment with cepharanthine decreases cytoadherence, Maurer’s clefts, apicoplast and S-glutathionylated protein pathways compared to initial and final controls (see Additional file 4).

### Microarray validation by qRT-PCR

**Figure 4:**
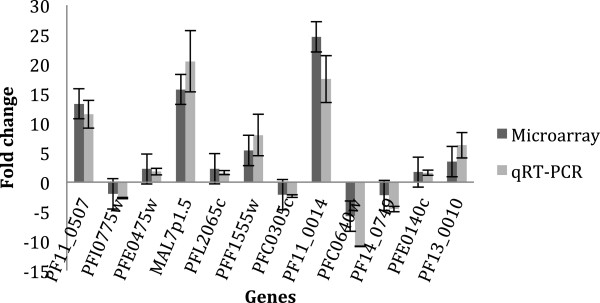
**Correlation of microarray and qRT-PCR results.** The relative expression of 12 genes is represented in three histograms by FC (Figures 4, 5 and 6). The qRT-PCR results are in light grey and the microarray results are in dark grey. The positives FC are over the horizontal line and the negative ones are under the horizontal line. The names of genes tested are indicated in abscissa. The histogram compares UT16 and T16; the correlation assessed by a linear model allows obtaining *p*-value of 8.67 × 10^-6^.

**Figure 5:**
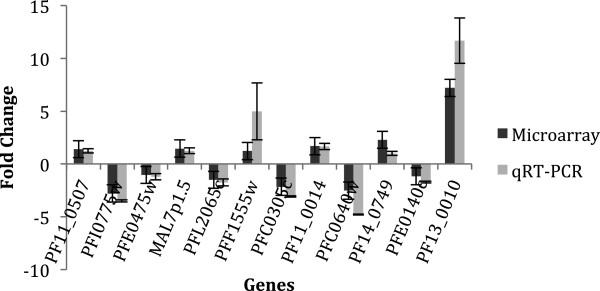
**Correlation of microarray and qRT-PCR results.** The relative expression of 12 genes is represented in these three histograms by FC (Figures 4, 5 and 6). The qRT-PCR results are in light grey and the microarray results are in dark grey. The positives FC are over the horizontal line and the negative ones are under the horizontal line. The names of genes tested are indicated in abscissa. The histogram compares UT8 and T16; the correlation assessed by a linear model allows obtaining *p*-value of 2.04 × 10^-6^

**Figure 6:**
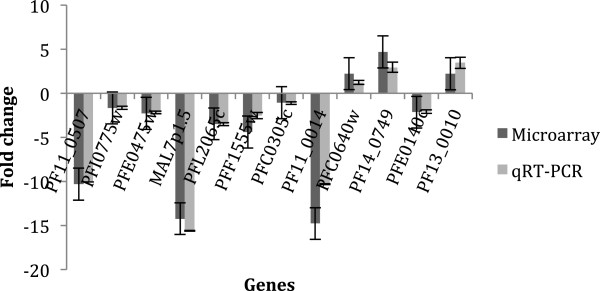
**Correlation of microarray and qRT-PCR results.** The relative expression of 12 genes is represented in these three histograms by FC (Figures 4, 5 and 6). The qRT-PCR results are in light grey and the microarray results are in dark grey. The positives FC are over the horizontal line and the negative ones are under the horizontal line. The names of genes tested are indicated in abscissa. The histogram compares UT8 and UT16; the correlation assessed by a linear model allows obtaining *p*-value of 3.13 × 10^-7^.

The differentially expressed transcripts were validated by qRT-PCR. The variation of expression of 12 transcripts was evaluated and compared with the microarray results. A good concordance was observed for all transcripts and conditions, as shown in Figure 4 (comparing UT16 and T16), Figure 5 (comparing UT8 and T16); and Figure 6 (comparing UT8 and UT16). The Spearman rank correlation method gives p-value of 8.67 × 10^-6^, 2.04 × 10^-6^ and 3.13 × 10^-7^ for the comparisons in Figures 4, 5 and 6, respectively.

## Discussion

A previous study had suggested that cepharanthine could have an antiplasmodial activity differing from those of available anti-malarial drugs [14]. This assumption was not contradicted by the evaluation of IC_50_ levels on strains that have various sensitivities to anti-malarial drugs. Indeed, assays performed on 3D7 strain (CQ-sensitive/MQ-resistant) and on the Asian W2, FCM29 and K1 strains (CQ-resistant/MQ-sensitive) showed that the IC_50_ ratios of cepharanthine on CQ and MQ moved depending on the stains. For example, the susceptibility of W2 to cepharanthine was higher than K1 whereas the opposite was observed with both CQ and MQ. Furthermore, morphologic characterization of cepharanthine impact on the life cycle of *Plasmodium* showed a particular effect depending on the parasite stage. Indeed, at any time of the parasite life cycle, when cepharanthine was incubated with a constant drug pressure, *Plasmodium* was blocked at the ring stages and parasitaemia followed down to 0% after a 96- to 144-hr period. When the drug was removed, the life cycle of the parasite returned to normal. This stage effect of cepharanthine is highlighted for the first time. This specificity has enabled the design of a microarray assay at the ring stage and on closely synchronized cultures.

At the transcriptional level, a tight synchronization of parasites was performed within a four-hour time-window, allowing good accuracy of microarray results and reflecting the quality of the study. The transcriptional assay was performed during a short time of eight hours and on a relatively stable parasitic stage. Indeed, the ring stage corresponded to the beginning of the *Plasmodium* life cycle in which gene expression is slightly modified, few metabolic pathways being established. Despite this, high variations of gene expression were observed between the three conditions studied. This transcriptional study highlights the set of genes whose expression is directly and indirectly disrupted by cepharanthine. Transcriptional analysis performed on ring stage confirmed that the parasitic blockage, microscopically observed, might be related to the metabolism pathways.

The limitations of this study are mainly due to *Plasmodium* model. The quickness of *Plasmodium* cycle and the constancy of the parasitic evolution explain the choice of working on short time. The design of the transcriptional experiment performed on ring stages was not easy, involving a large amount of synchronized ring stages and a sufficient incubation time of cepharanthine. So, time points have been chosen to be far from the invasion by merozoites and the transformation into trophozoite stages.

Asahi *et al.* studied factors controlling intra-erythrocyte development of *P. falciparum* [27]. They used various chemically defined mediums and after transcriptome profiling, they found 26 transcripts predicted to be associated with the schizogony stunting. Two of their over-expressed transcripts were also significantly upregulated in the study presented here (CSP-TRAP PFC0640w and MYND finger protein PFF0350w). The upregulation of these two genes could be responsible of the blockage of parasites into ring stage by cepharanthine.

A parasitostatic effect on ring stage was also observed after a treatment with a natural triterpene, named limonene [28]. The freezing of parasite progression from the ring to the trophozoite stage by the limonene could involve a decrease of isoprenylation of proteins as well as rhoptry-associated proteins (RAP). In the study described here, the isoprenylation metabolic pathway was significantly decreased by cepharanthine. This biochemical pathway localized in the apicoplast of the parasite is an interesting target because of its absence in the human host. Moreover, the isoprenoid precursor synthesis is essential for the parasite survival [28, 29].

At the genomic level, four enzymes of the glycolysis (glucose-6-phosphate isomerase PF14_0341, triose phosphate isomerase PF14_0378, phosphoglycerate mutase PF11_0208 and enolase PF10_0155) and one enzyme of the gluconeogenesis (phosphoenolpyruvate carboxykinase PF13_0234) were down-regulated by cepharanthine and could be responsible for this parasitic blockage. For example, the glucose-6-phosphate isomerase interferes with the second step of the glycolysis corresponding to the conversion of glucose 6-phosphate (G6P) in fructose 6-phosphate (F6P) [30]. In the GO analysis, the gluconeogenesis was enriched and in the metabolic pathway, the glycolysis was significantly impacted by cepharanthine. According to Bozdech *et al.,* genes of the glycolysis are induced during the ring and early trophozoite stages [31]. A downregulation of genes of this metabolic pathway could be responsible for inhibiting the passage of the ring stage to trophozoite stage.

Among the gene families whose expression is downregulated by cepharanthine, some were quoted as essential for parasite survival. The carbon catabolite repressor protein 4-associated factor 1 (CAF-1) exerts a regulation mainly on the red blood cell invasion by merozoite [32]. The cyclin-dependent kinase cdc2 possesses a regulatory function on the cell cycle evolution in *Plasmodium* [33, 34]. The caseinolytic proteases (ClpB) are chaperones located in the apicoplast and involved in the cellular homeostasis [35]. The heat shock proteins (HSP) 40, 70, 90 are among chaperones playing an important role in the cellular processes of the parasite survival and pathogenicity [26, 36, 37]. The SURFIN corresponds to an antigen transported to the red blood cell surface by Maurer’s clefts and located at the merozoite apex. This antigen seems fundamental for the merozoite invasion and parasite survival [38–41]. Others were proposed as potential anti-malarial targets. The acyl-CoA binding proteins (ACBP1 and ACBP2) are involved in the *de novo* apicoplast fatty acid biosynthesis [42]. The aquaglyceroporins are responsible of the urea and glycerol transport [43]. The 4-(cytidine 5’-diphospho)-2-C-methyl-D-erythritol kinases (CMK) play a catalytic role in the biosynthesis of isopentenyl pyrophosphate [44]. Two antigens were proposed as malaria vaccine candidates. The glycosylphosphatidylinositol (GPI)-anchored proteins are responsible of the membranous protein binding and merozoite invasion [45]. The rhoptry-associated proteins (RAP) are immunogenic and involved in the merozoite invasion too [46, 47]. As both protein targets are well characterized, a Western blot experiment could be performed to confirm the results obtained with the transcriptional assay.

The pathways analysis showed that few pathways are upregulated by cepharanthine. Thus, for a better knowledge of the cepharanthine mechanism and its possible targets, the study focused on genes involved in parasite survival and virulence, downregulated by cepharanthine. The genes’ coding for proteins exported to the host cell by Maurer’s clefts, named the exportome [26], are generally downregulated by anti-malarials. These clefts are membranous structures involved in the export of parasitic proteins to the erythrocyte membrane [38] and are widely affected by cepharanthine treatment (GO over-represented with a p-value of 10^-25^). This expression modulation is observed for structural proteins of Maurer clefts (*Pf*mc-2TM [26, 48–50] and antigen 332) and proteins exported by Maurer’s clefts (ring-infected surface antigen (RESA)-like with PHIST and Dna J domain [26], *Plasmodium* helical interspersed subtelomeric (PHIST) a, b and c [26], kinase named FIKK [26, 51–54]). Currently, there are no available anti-malarial drugs acting directly on the transport mediated by Maurer’s clefts. It has been shown that the Maurer’s clefts decreased in number but not in function, when treated with artesunate, quinine and piperaquine [55]. However, this observation does not represent the main mechanism of action of these three drugs. It will be necessary to study the functional modifications of these structures before and after treatment by cepharanthine, with additional methods as imaging techniques, to explain the relationship between cepharanthine and Maurer’s clefts.

The GOs of host cell plasma membrane and antigenic variations, containing mainly genes coding for *Pf*EMP1, *RIFIN* and *STEVOR*, were significantly under-represented in this study (10^-30^ < p < 10^-4^). *Pf*EMP1 is addressed to the host erythrocytes by Maurer’s clefts and is responsible of cyto-adherence inducing cerebral malaria [56]. Rifin and stevor contribute to the antigenic variation of *Plasmodium* conferring its adaptability towards all the antiplasmodial treatments. The downregulation of these three kinds of genes by cepharanthine seems interesting for the inhibition of *Plasmodium* virulence. Furthermore, cepharanthine seems to inhibit genes related to pathways involving mitochondrion (p = 1.5 × 10^-4^) and apicoplast (p = 8.4 × 10^-5^). Proteomic studies showed that these organelles seem to be targeted by doxycycline [57]. Moreover, targeting mitochondrion electron transport, atovaquone induced a static state on the ring stages [58]. The parasitostatic effect observed with cepharanthine treatment could be due to its activity on mitochondrion but also on apicoplast. Indeed, a “delay death” has been observed with drugs inhibiting apicoplast as tetracycline and fosmidomycin [59]. The mechanism involved in this phenomenon is not yet elucidated but it is traduced by a blockage of parasitic growth after the reinvasion of erythrocytes [29]. This property has also been observed with cepharanthine that induced a blockage of ring stages during the second parasitic cycle. Moreover, being absent in humans, the apicoplast is a specific target. The compounds acting on this organelle could induce good safety in humans. So, it would be interesting to study the effect of cepharanthine on this organelle with further complementary and specific experiments to a better understanding and characterization of this inhibition of isoprenoid precursor biosynthesis.

In the goal to identify the real targets of cepharanthine, the potential targets underlined at the transcriptomic level in this work have to be confirmed at the proteomic level. Moreover the use of imagery technics could be interesting to check the activity of cepharanthine on some targets as Maurer clefts and mitochondrion. Elsewhere the activity of cepharanthine on cytoadherence could be evaluated *in vitro* and *in vivo* using the Palo-Alto (FUP)1 *P. falciparum* strain [60].

Previous pharmacokinetic studies performed in mouse [61], beagle dog and human [62] showed a quite long elimination half-life for cepharanthine that could be a potential candidate in combination with faster-acting anti-malarials in the treatment of multidrug-resistant *falciparum* malaria in seriously ill patients (ACT combination for example) [11].

## Conclusions

In this work, the bisbenzylisoquinoline, called cepharanthine, exerted an antiplasmodial activity against four strains of *P. falciparum*. There is no bisbenzylisoquinoline currently used in the treatment of malaria, so, in light of the development of resistance against standard anti-malarials and the search for new drugs with a novel mechanism of action, cepharanthine could be a potential drug lead. Phenotypic and transcriptional assays showed for the first time a blocking effect of cepharanthine into ring stage. This parasitostatic effect seems to involve the isoprenoid and glycolysis metabolic pathways probably by the inhibition of various enzymes of these pathways. The gene expression profiling by microarray showed that cepharanthine could interfere with several important functions for *Plasmodium* survival and virulence as the mitochondrion, apicoplast, cytoadherence antigenic variation and Maurer’s clefts. Further studies directly targeting the genes of interest are needed to confirm the potential targets. These original potential targets could allow for the use of cepharanthine in a new drug combination. The implementation of complementary approaches as proteomic or metabolomics studies for example, is necessary to confirm results obtained at the genomic level.

## Electronic supplementary material

Additional file 1: **Efficiencies and sequences of the forward (F) and reverse (R) primers.** For each probe, the new and old accession numbers, the name and acid nucleic sequences are given. R^2^, slope and efficiency were calculated to choose the best concentration to use for the qRT-PCR experiment. (PDF 71 KB)

Additional file 2: **Fold-change of probes comparing the three conditions UT8, UT16 and T16.** (PDF 833 KB)

Additional file 3: **Hierarchical classification of Gene Ontology terms enriched during the transcriptomic experiment.** (PDF 943 KB)

Additional file 4: **Differences of expression in pathways due to cepharanthine.** These three histograms compare the expression of pathways by pairs of experimental conditions. Horizontal bars lengths correspond to the number of genes up-regulated (red) and down-regulated (green) from each metabolic pathway. FC = fold change, UT = untreated, T = treated. (PDF 92 KB)

Additional file 5: **Metabolic pathways modified by cepharanthine.** Over-represented pathways were grouped into functions according to the Malaria Parasite Metabolic Pathways database. P-values were obtained by Fisher exact t-tests. The threshold of significance is 0.05. (PDF 102 KB)

## Abbreviations

ACBP: Acyl-CoA binding protein
ANOVA: Analysis of variance
CAF-1: Carbon catabolite repressor protein 4-associated factor 1
ClpB: Caseinolytic protease
CQ: Chloroquine
FC: Fold change
F6P: Fructose 6-phosphate
G6P: Glucose 6-phosphate
GO: Gene ontology
GPI: Glycosylphosphatidylinositol
HSP: Heat shock protein
IC_50_: Concentration inhibiting 50% of parasitic growth
MQ: Mefloquine
pmi: Merozoite post invasion
PfEMP1: Plasmodium falciparum erythrocyte membrane protein-1
Pfmc-2TM: Plasmodium falciparum Maurer’s clefts two-transmembrane protein
PHIST: *Plasmodium* helical interspersed subtelomeric protein
qRT-PCR: Quantitative real time polymerase chain reaction
RAP: Rhoptry-associated proteins
RESA: Ring-infected surface antigen
RSD: Relative standard deviation
SURFIN: Surface associated interspersed gene
T16: Samples treated by cepharanthine
UT8: Untreated controls arrested 8 hr pmi by merozoites
UT16: Untreated controls arrested 16 hr pmi by merozoites.
