# Supplementary material for: In vitro antiplasmodial activity of cepharanthine
Source: Malar J. 2014 Aug 22;13:327. doi: 10.1186/1475-2875-13-327 (PMC4152577; doi:10.1186/1475-2875-13-327)
Supplement: Supplementary file 1 — Additional file 1: Efficiencies and sequences of the forward (F) and reverse (R) primers. For each probe, the new and old accession numbers, the name and acid nucleic sequences are given. R2, slope and efficiency were calculated to choose the best concentration to use for the qRT-PCR experiment. (PDF 71 KB) [file 12936_2014_3366_MOESM1_ESM.pdf]

Additional file 1: Efficiencies and sequences of the forward (F) and reverse (R) primers.

Accession number	New accession number	Name	Forward (F) and reverse ® Primers	R^2	Slope	Efficiency	Concentration
PF11_0507	PF3D7_1149000	Antigen 332. DBL-like protein (Pf332)	F-GGGTCTTGGTATATAAGCATTCTTTGA<br>R-TCCCCGTACCATGAACACG	0.99	-3.44	95.12	0.5 µM
PFI0775w	PF3D7_0915800	Glycolipid transfer protein	F-TGTGCACAAGAAGCATATAATGAAGTATTA<br>R-AAGTACAGTGGAAGCTTGTTGATGAG	0.98	-3.33	99.57	
PFE0475w	PF3D7_0509600	Asparagine-tRNA ligase	F-ATCAATTACCCAACTCATATAAAACCATT<br>R-CTCTTTCCGACCCTCCTACAACT	0.99	-3.40	96.80	
MAL7P1.5	PF3D7_0701600	Pfmc-2TM Maurer's cleft two transmembrane protein (MC-2TM)	F-CAACCTTATTACATCTTTTACTCTGCATTTA<br>R-AAACCTATTTTCATGTCAGGAAAATAACT	0.99	-3.42	95.84	
PFL2065c	PF3D7_1242900	Mitochondrial import inner membrane translocase subunit	F-AAATTGGACCAAAATTAAGTTCAAGTG<br>R-TCTGTTCCAAGCGCTGATTTAAG	0.99	-3.32	100.15	
PFF1555w	PF3D7_0632000	Rifin (RIF)	F-TATTGGAATCGGTTTTTACTGCAC<br>R-AATGGCGAACAAATCACAGGT	0.99	-3.44	95.37	
PFC0305w	PF3D7_0307300	EB1 homolog	F-AAGATGATGAATCTATGGAACCTAACAAT<br>R-CAGTAGGTGGCGTAGTCTATCGAAC	0.98	-3.29	101.45	0.9 µM
PF11_0014	PF3D7_1100800	Pfmc-2TM Maurer's cleft two transmembrane protein (MC-2TM)	F-TTGTGGGTTTAATTGAAGCATCTTCT<br>R-ACATAAAAACTGGAAAAATACATAGAGGATAAG	1.00	-3.38	97.77	
PFC0640w	PF3D7_0315200	Circumsporozoite- and TRAP-related protein (CTRP)	F-GGAATTATGCGTTTCGCCAAAT<br>R-GATGGGTAGCACCACCATATCC	0.99	-3.36	98.38	
PF14_0749	PF3D7_1477800	Acyl-CoA binding protein (ACBP)	F-AGAACTATAAATTTGCCCAATGAACTTAA<br>R-TTATGTGCGCTTGGTTCCTTT	1.00	-3.34	99.12	
PFE0140c	PF3D7_0502900	Mitochondrial inner membrane TIM10 associated protein	F-AGAAGTTTTCTGAACAAATGGGAAA<br>R-TGTGGACGCCTTGGTTACAA	0.99	-3.40	96.71	
PF13_0010	PF3D7_1301200	Glycophorin binding protein (GBPH2)	F-GAGACAAATACCAACAAGATGCAGATT<br>R-TGCGCACATTTCCTTAATGAAG	0.98	-3.41	96.45	

For each probe, the new and old accession numbers, the name and acid nucleic sequences are given.
R^2, slope and efficiency were calulated to chose the best concentation to use for the qRT-PCR experiment.
