# Supplementary material for: In vitro antiplasmodial activity of cepharanthine
Source: Malar J. 2014 Aug 22;13:327. doi: 10.1186/1475-2875-13-327 (PMC4152577; doi:10.1186/1475-2875-13-327)
Supplement: Supplementary file 2 — Additional file 2: Fold-change of probes comparing the three conditions UT8, UT16 and T16. (PDF 833 KB) [file 12936_2014_3366_MOESM2_ESM.pdf]

Additional file 2 : Fold-change of probes comparing the three conditions UT8, UT16 and T16.

	[UT8] vs [UT16]		[UT16] vs [T16]		[UT8] vs [T16]	
**Accession number**	**Fold-change**	**Regulation**	**Fold-change**	**Regulation**	**Fold-change**	**Regulation**
Pfa3D7;chr12;PFL0740c;Pf_66_66	-4.8484178	Down	3.7848868	Up	-1.2220087	Down
Pfa3D7;chr8;PF08_0131;Pf_539_539	-3.18109	Down	5.7941246	Up	1.8084611	Up
Pfa3D7;chr8;PF08_0131;Pf_499_499	-3.368678	Down	6.106556	Up	1.991527	Up
Pfa3D7;chr12;PFL1940w;Pf_1437_1437	2.5659366	Up	-1.7457757	Down	1.3457848	Up
Pfa3D7;chr12;PFL1940w;Pf_1506_1506	2.1103203	Up	-1.7760226	Down	1.090887	Up
Pfa3D7;chr13;PF13_0316;Pf_283_283	1.2377503	Up	1.6738536	Up	2.0536761	Up
Pfa3D7;chr14;PF14_0205;Pf_230_230	-1.2139212	Down	2.085605	Up	1.7269375	Up
Pfa3D7;pfal_chr5;PFE0150c;Pf_1388_1388	-2.0534332	Down	2.1036472	Up	1.0280988	Up
Pfa3D7;pfal_chr5;PFE0150c;Pf_1543_1543	-2.2090895	Down	2.1601088	Up	-1.0318563	Down
Pfa3D7;chr6;PFF1335c;Pf_460_460	-1.1448604	Down	2.4626384	Up	2.2310085	Up
Pfa3D7;chr6;PFF1335c;Pf_500_500	-1.131614	Down	2.375103	Up	2.1251514	Up
Pfa3D7;chr6;PFF0700c;Pf_400_400	-1.409991	Down	2.8050072	Up	1.8976911	Up
Pfa3D7;chr11;PF11_0437;Pf_230_230	-1.2535751	Down	2.6016674	Up	2.0436296	Up
Pfa3D7;pfal_chr3;PFC0200w;Pf_85_85	1.0178117	Up	2.1894875	Up	2.208473	Up
Pfa3D7;chr7;PF07_0047;Pf_3388_3388	-2.4469323	Down	2.5648253	Up	1.0915327	Up
Pfa3D7;chr7;PF07_0047;Pf_3219_3219	-2.3056035	Down	2.7850225	Up	1.1916834	Up
Pfa3D7;chr13;PF13_0218;Pf_2719_2719	-1.933365	Down	2.0417395	Up	-1.0062978	Down
Pfa3D7;pfal_chr1;PFA0590w;Pf_5295_5295	-3.920893	Down	2.056374	Up	-2.005876	Down
Pfa3D7;pfal_chr1;PFA0590w;Pf_5335_5335	-3.599664	Down	1.971297	Up	-1.7950262	Down
Pfa3D7;chr12;PFL0495c;Pf_1781_1781	-1.7718472	Down	2.1842325	Up	1.1043102	Up
Pfa3D7;chr8;PF08_0078;Pf_3993_3993	-2.805452	Down	2.6171887	Up	-1.0965753	Down
Pfa3D7;chr14;PF14_0282;Pf_7816_7816	2.7604315	Up	-1.7508823	Down	1.5259942	Up
Pfa3D7;pfal_chr3;PFC0360w;Pf_81_81	-2.9500182	Down	4.058666	Up	1.5572621	Up
Pfa3D7;chr14;PF14_0749;Pf_62_62	4.6855683	Up	-2.2340953	Down	2.2834814	Up
Pfa3D7;chr10;PF10_0015;Pf_31_31	9.930037	Up	-2.690503	Down	3.9468307	Up
Pfa3D7;chr10;PF10_0014;Pf_353_353	7.4545307	Up	-2.4111788	Down	3.2411196	Up
Pfa3D7;chr10;PF10_0016;Pf_61_61	-5.237448	Down	12.704781	Up	2.4922802	Up
Pfa3D7;chr14;PF14_0761;Pf_1648_1648	5.676916	Up	-2.4295566	Down	2.4415002	Up
Pfa3D7;chr14;PF14_0761;Pf_1608_1608	6.5484943	Up	-2.7429876	Down	2.2023218	Up
Pfa3D7;chr7;PF07_0129;Pf_2308_2308	-2.0054097	Down	2.9375286	Up	1.5628653	Up
Pfa3D7;chr7;PF07_0129;Pf_2070_2070	-1.9193254	Down	3.2325606	Up	1.6786796	Up
Pfa3D7;pfal_chr4;PFD0085c;Pf_2380_2380	5.235368	Up	-2.1871154	Down	2.3028584	Up
Pfa3D7;chr12;PFL0035c;Pf_2565_2565	5.714501	Up	-1.5920793	Down	3.5397215	Up
Pfa3D7;chr12;PFL0035c;Pf_2653_2653	4.8016515	Up	-1.576022	Down	3.0131967	Up
Pfa3D7;pfal_chr2;PFB0695c;Pf_2239_2239	2.343231	Up	-1.8930925	Down	1.1916353	Up
Pfa3D7;chr7;MAL7P1.164;Pf_2406_2406	3.0901732	Up	-2.8014386	Down	-1.0263555	Down
Pfa3D7;chr14;PF14_0386;Pf_2125_2125	-4.310942	Down	2.8274415	Up	-1.5860776	Down
Pfa3D7;chr14;PF14_0386;Pf_2044_2044	-4.9181843	Down	3.0024047	Up	-1.5981996	Down
Pfa3D7;pfal_chr4;PFD0755c;Pf_589_589	-3.0512896	Down	3.3973582	Up	1.1141076	Up
Pfa3D7;chr8;PF08_0062;Pf_757_757	1.1123145	Up	-2.5447924	Down	-2.619558	Down
Pfa3D7;chr10;PF10_0051;Pf_788_788	-2.4825528	Down	2.2802505	Up	-1.0539793	Down
Pfa3D7;chr10;PF10_0051;Pf_924_924	-2.2413282	Down	2.108382	Up	-1.0974892	Down
Pfa3D7;chr13;MAL13P1.297;Pf_430_430	1.5265839	Up	-2.3325293	Down	-1.4439714	Down
Pfa3D7;chr14;PF14_0399;Pf_429_429	-2.5344396	Down	1.8953149	Up	-1.3657614	Down
Pfa3D7;chr7;MAL7P1.178;Pf_1757_1757	2.3703985	Up	-1.8916202	Down	1.1940534	Up
Pfa3D7;chr12;PFL0420w;Pf_4624_4624	-3.369896	Down	1.6191291	Up	-2.0025115	Down
Pfa3D7;pfal_chr5;PFE0775c;Pf_2768_2768	-1.2761834	Down	-1.8091094	Down	-2.223313	Down
Pfa3D7;chr14;PF14_0015;Pf_1342_1342	-4.0356464	Down	-1.8003854	Down	-6.711135	Down
Pfa3D7;chr11;PF11_0197;Pf_963_963	-2.2123315	Down	2.6209455	Up	1.190923	Up
Pfa3D7;chr14;PF14_0222;Pf_1598_1598	1.3782362	Up	-2.3361578	Down	-1.6413275	Down
Pfa3D7;chr14;PF14_0106;Pf_436_436	1.4683472	Up	-2.3654342	Down	-1.5139441	Down
Pfa3D7;chr10;PF10_0102;Pf_382_382	-1.3739172	Down	-2.4523323	Down	-3.1449745	Down
Pfa3D7;chr11;PF11_0506;Pf_1149_1149	-8.159957	Down	13.63064	Up	1.9020733	Up
Pfa3D7;chr11;PF11_0507;Pf_16267_16267	-9.219649	Down	18.883167	Up	1.994017	Up

Accession number	[UT8] vs [UT16] Fold-change	Regulation	[UT16] vs [T16] Fold-change	Regulation	[UT8] vs [T16] Fold-change	Regulation
Pfa3D7;chr11;PF11_0506;Pf_1612_1612	-9.552233	Down	13.888245	Up	1.4566249	Up
Pfa3D7;chr11;PF11_0507;Pf_16307_16307	-10.332231	Down	13.265642	Up	1.4026742	Up
Pfa3D7;chr14;PF14_0270;Pf_1100_1100	-2.4729767	Down	2.2484734	Up	-1.0965289	Down
Pfa3D7;chr14;PF14_0270;Pf_1497_1497	-2.430713	Down	2.0940263	Up	-1.0768716	Down
Pfa3D7;chr10;PF10_0332;Pf_405_405	-1.2505171	Down	-1.6975592	Down	-2.069141	Down
Pfa3D7;chr11;PF11_0106;Pf_156_156	1.2180333	Up	1.8744457	Up	2.21173	Up
Pfa3D7;pfal_chr2;PFB0855c;Pf_3385_3385	-2.0376177	Down	2.0633554	Up	-1.0602692	Down
Pfa3D7;chr11;PF11_0338;Pf_548_548	-3.7283804	Down	2.9891593	Up	-1.0893573	Down
Pfa3D7;chr11;PF11_0338;Pf_350_350	-4.0643654	Down	3.3092272	Up	-1.1296593	Down
Pfa3D7;chr8;PF08_0060;Pf_6522_6522	-9.667169	Down	1.6204638	Up	-4.969221	Down
Pfa3D7;chr8;PF08_0060;Pf_6582_6582	-9.026187	Down	1.5992646	Up	-5.244675	Down
Pfa3D7;chr8;PF08_0060;Pf_6622_6622	-9.176753	Down	1.5697919	Up	-5.380583	Down
Pfa3D7;pfal_chr9;PFI1520w;Pf_781_781	1.2106135	Up	-7.1329007	Down	-6.141268	Down
Pfa3D7;pfal_chr5;PFE0475w;Pf_2093_2093	-2.2880776	Down	2.2255926	Up	-1.0407593	Down
Pfa3D7;pfal_chr5;PFE0475w;Pf_1434_1434	-2.2870765	Down	2.5448418	Up	1.0802143	Up
Pfa3D7;chr13;MAL13P1.221;Pf_481_481	-2.2544932	Down	2.2969882	Up	-1.0349759	Down
Pfa3D7;chr13;MAL13P1.221;Pf_969_969	-2.2511053	Down	2.0951915	Up	-1.0347803	Down
Pfa3D7;pfal_chr5;PFE0715w;Pf_3089_3089	-2.3607826	Down	2.203268	Up	-1.1342673	Down
Pfa3D7;pfal_chr5;PFE0715w;Pf_3278_3278	-2.9340024	Down	2.253394	Up	-1.3439522	Down
Pfa3D7;chr8;PF08_0042;Pf_2495_2495	2.027157	Up	-1.6456604	Down	1.2172412	Up
Pfa3D7;chr12;PFL1490w;Pf_2648_2648	2.1061456	Up	-1.8013475	Down	1.1275156	Up
Pfa3D7;pfal_chr3;PFC0800w;Pf_1010_1010	-3.6815503	Down	4.362862	Up	1.4162997	Up
Pfa3D7;pfal_chr3;PFC0800w;Pf_970_970	-3.7346303	Down	4.325012	Up	1.2115644	Up
Pfa3D7;chr14;PF14_0241;Pf_252_252	-1.4184198	Down	2.120492	Up	1.4446173	Up
Pfa3D7;pfal_chr1;PFA0400c;Pf_367_367	-1.6726172	Down	2.4000995	Up	1.4753561	Up
Pfa3D7;pfal_chr1;PFA0400c;Pf_508_508	-1.6067547	Down	2.1722896	Up	1.3905778	Up
Pfa3D7;pfal_chr2;PFB0505c;Pf_544_544	1.2886252	Up	-2.2049518	Down	-1.758629	Down
Pfa3D7;pfal_chr2;PFB0505c;Pf_959_959	1.0997043	Up	-2.060984	Down	-1.7587901	Down
Pfa3D7;chr14;PF14_0413;Pf_2410_2410	-3.3524265	Down	3.36279	Up	-1.0049971	Down
Pfa3D7;chr11;PF11_0239;Pf_4548_4548	1.7725319	Up	-2.3325188	Down	-1.355773	Down
Pfa3D7;chr11;PF11_0239;Pf_4616_4616	1.775415	Up	-2.422201	Down	-1.3991723	Down
Pfa3D7;chr12;PFL1845c;Pf_581_581	-1.5329071	Down	2.4329114	Up	1.4986154	Up
Pfa3D7;chr12;PFL1845c;Pf_514_514	-1.7502875	Down	2.5791748	Up	1.4281327	Up
Pfa3D7;chr14;PF14_0181;Pf_259_259	-2.4171858	Down	1.9200631	Up	-1.4591249	Down
Pfa3D7;pfal_chr5;PFE0980c;Pf_7371_7371	-2.3933723	Down	1.8939664	Up	-1.221793	Down
Pfa3D7;pfal_chr1;PFA0355w;Pf_811_811	-2.142609	Down	2.0217683	Up	-1.0855371	Down
Pfa3D7;pfal_chr5;PFE0195w;Pf_7083_7083	-4.636446	Down	5.8780956	Up	1.2577496	Up
Pfa3D7;pfal_chr5;PFE0195w;Pf_7123_7123	-4.5288863	Down	6.3536763	Up	1.4415079	Up
Pfa3D7;chr14;PF14_0374;Pf_3114_3114	5.4755526	Up	-2.5069032	Down	2.1539216	Up
Pfa3D7;pfal_chr4;PFD0865c;Pf_2027_2027	-2.9015431	Down	1.9781057	Up	-1.3546076	Down
Pfa3D7;pfal_chr4;PFD0865c;Pf_1924_1924	-2.9798071	Down	1.8940889	Up	-1.542864	Down
Pfa3D7;chr12;PFL0800c;Pf_458_458	-1.0260671	Down	-5.437529	Down	-4.860877	Down
Pfa3D7;chr12;PFL0800c;Pf_427_427	-1.1652251	Down	-5.426187	Down	-5.0002947	Down
Pfa3D7;chr7;PF07_0034;Pf_674_674	1.8309605	Up	-2.3671343	Down	-1.2602533	Down
Pfa3D7;chr7;PF07_0034;Pf_842_842	1.5514245	Up	-2.4119093	Down	-1.4790652	Down
Pfa3D7;chr14;PF14_0346;Pf_2498_2498	2.015397	Up	-1.6689092	Down	1.2935961	Up
Pfa3D7;chr12;PFL1545c;Pf_1749_1749	-2.994795	Down	4.1974277	Up	1.3115172	Up
Pfa3D7;chr12;PFL2510w;Pf_1041_1041	1.643015	Up	-2.6208484	Down	-1.5289403	Down
Pfa3D7;chr14;PF14_0020;Pf_1059_1059	-2.2037373	Down	1.4462495	Up	-1.5240387	Down
Pfa3D7;chr14;PF14_0020;Pf_1149_1149	-2.162159	Down	1.3797529	Up	-1.5096707	Down
Pfa3D7;chr13;MAL13P1.86;Pf_2550_2550	-2.2287762	Down	2.117759	Up	-1.0260941	Down
Pfa3D7;chr13;MAL13P1.86;Pf_2630_2630	-3.03728	Down	2.644034	Up	-1.1053423	Down
Pfa3D7;chr6;PFF1105c;Pf_1441_1441	-1.7644337	Down	2.2830737	Up	1.2790003	Up
Pfa3D7;pfal_chr3;PFC0210c;Pf_856_856	-1.1478685	Down	-1.7996672	Down	-2.091482	Down
Pfa3D7;pfal_chr3;PFC0210c;Pf_1002_1002	-1.0098943	Down	-1.9801376	Down	-2.0796504	Down
Pfa3D7;chr8;PF08_0063;Pf_2528_2528	-2.1890388	Down	2.397671	Up	1.0168694	Up

Accession number	[UT8] vs [UT16] Fold-change	Regulation	[UT16] vs [T16] Fold-change	Regulation	[UT8] vs [T16] Fold-change	Regulation
Pfa3D7;chr8;PF08_0063;Pf_3068_3068	-2.1271167	Down	2.1125293	Up	1.0580487	Up
Pfa3D7;chr11;PF11_0258;Pf_813_813	-2.3289323	Down	2.105642	Up	-1.0649086	Down
Pfa3D7;chr13;PF13_0180;Pf_709_709	-3.0022783	Down	3.0282195	Up	-1.020611	Down
Pfa3D7;chr13;PF13_0180;Pf_493_493	-2.4538662	Down	2.8499217	Up	1.1165445	Up
Pfa3D7;pfal_chr4;PFD0075w;Pf_692_692	-12.194834	Down	26.870354	Up	2.7173004	Up
Pfa3D7;pfal_chr4;PFD0075w;Pf_861_861	-13.820117	Down	24.788359	Up	1.9616632	Up
Pfa3D7;chr8;MAL8P1.130;Pf_1138_1138	-1.459589	Down	-1.4661175	Down	-2.1609893	Down
Pfa3D7;pfal_chr5;PFE0725c;Pf_373_373	-1.5207398	Down	-1.9410533	Down	-2.5798047	Down
Pfa3D7;chr8;PF08_0097;Pf_1508_1508	-3.5459006	Down	1.9207126	Up	-1.9037209	Down
Pfa3D7;chr14;PF14_0325;Pf_833_833	2.0248637	Up	-2.1063783	Down	1.0327139	Up
Pfa3D7;chr6;PFF0540c;Pf_463_463	1.0842373	Up	-2.4763675	Down	-2.4143996	Down
Pfa3D7;pfal_chr4;PFD0225w;Pf_12244_1224	-2.71353	Down	3.7513847	Up	1.3670329	Up
Pfa3D7;chr13;MAL13P1.298;Pf_6312_6312	2.487792	Up	-1.8428885	Down	1.3233564	Up
Pfa3D7;pfal_chr5;PFE1205c;Pf_224_224	1.5800968	Up	-2.3219914	Down	-1.5303856	Down
Pfa3D7;chr13;PF13_0172;Pf_1482_1482	1.7941933	Up	-3.0485024	Down	-1.7412454	Down
Pfa3D7;chr10;PF10_0211;Pf_13202_13202	-3.1294944	Down	2.5206063	Up	-1.1402872	Down
Pfa3D7;chr7;MAL7P1.31;Pf_363_363	-2.0858319	Down	2.370894	Up	1.2015501	Up
Pfa3D7;chr12;PFL0220c;Pf_497_497	-1.1497895	Down	-1.9433736	Down	-2.0543227	Down
Pfa3D7;chr14;PF14_0355;Pf_749_749	2.75447	Up	-1.9851663	Down	1.2492992	Up
Pfa3D7;pfal_chr9;PFI0390c;Pf_934_934	1.6097925	Up	-2.1065776	Down	-1.4188228	Down
Pfa3D7;chr11;PF11_0333;Pf_4283_4283	-2.4219465	Down	1.4449693	Up	-1.677392	Down
Pfa3D7;chr7;PF07_0075;Pf_1294_1294	-2.1014776	Down	2.4763978	Up	1.1606915	Up
Pfa3D7;pfal_chr4;PFD0225w;Pf_12121_1212	-1.638824	Down	2.8897648	Up	1.6759803	Up
Pfa3D7;pfal_chr3;PFC0240c;Pf_2799_2799	2.4496427	Up	-2.0021162	Down	1.1808063	Up
Pfa3D7;chr11;PF11_0167;Pf_175_175	2.5296443	Up	-2.6649942	Down	-1.0368993	Down
Pfa3D7;chr13;PF13_0230;Pf_2792_2792	-2.6162026	Down	2.3919704	Up	1.0234826	Up
Pfa3D7;pfal_chr1;PFA0205w;Pf_2257_2257	1.2301638	Up	-2.0724106	Down	-1.6779047	Down
Pfa3D7;chr14;PF14_0607;Pf_2934_2934	2.0901983	Up	-1.5287286	Down	1.4769276	Up
Pfa3D7;pfal_chr9;PFI1560c;Pf_3626_3626	2.7053678	Up	-2.7407746	Down	-1.0654181	Down
Pfa3D7;chr14;PF14_0315;Pf_16307_16307	-2.1841793	Down	1.9681735	Up	-1.1469578	Down
Pfa3D7;pfal_chr9;PFI1560c;Pf_3666_3666	3.3414366	Up	-2.7689488	Down	1.0250536	Up
Pfa3D7;pfal_chr2;PFB0675w;Pf_4010_4010	2.1733637	Up	-2.0378308	Down	-1.0396227	Down
Pfa3D7;chr8;MAL8P1.139;Pf_17515_17515	-1.0493094	Down	2.148863	Up	1.8133233	Up
Pfa3D7;chr14;PF14_0355;Pf_709_709	2.698312	Up	-1.998153	Down	1.2824385	Up
Pfa3D7;chr10;PF10_0212;Pf_13219_13219	-2.720801	Down	2.3445463	Up	-1.1358635	Down
Pfa3D7;pfal_chr3;PFC0240c;Pf_2719_2719	2.5393207	Up	-1.9222726	Down	1.24701	Up
Pfa3D7;chr7;MAL7P1.31;Pf_121_121	-1.9573852	Down	2.6235075	Up	1.3174237	Up
Pfa3D7;chr8;MAL8P1.139;Pf_17662_17662	-1.5390962	Down	2.5128462	Up	1.527843	Up
Pfa3D7;chr6;PFF0870w;Pf_2321_2321	2.2781224	Up	-1.7701235	Down	1.4422482	Up
Pfa3D7;chr11;PF11_0347;Pf_2750_2750	2.5089602	Up	-1.8434294	Down	1.3524292	Up
Pfa3D7;chr14;PF14_0251;Pf_280_280	2.8934305	Up	-1.9729391	Down	1.3963441	Up
Pfa3D7;pfal_chr5;PFE1245w;Pf_1554_1554	-3.096372	Down	2.228078	Up	-1.323914	Down
Pfa3D7;pfal_chr1;PFA0570w;Pf_1773_1773	-2.7393305	Down	2.0090425	Up	-1.434231	Down
Pfa3D7;chr14;PF14_0567;Pf_924_924	2.1231096	Up	-1.5971124	Down	1.2201458	Up
Pfa3D7;chr10;PF10_0233;Pf_1394_1394	-3.457879	Down	2.7720244	Up	-1.0197128	Down
Pfa3D7;chr13;MAL13P1.321;Pf_2367_2367	2.2188902	Up	-1.7250057	Down	1.2022133	Up
Pfa3D7;chr12;PFL1300c;Pf_1301_1301	-2.3619897	Down	2.3405237	Up	1.1062921	Up
Pfa3D7;chr12;PFL1865w;Pf_5342_5342	-2.3375115	Down	1.7336705	Up	-1.4572958	Down
Pfa3D7;chr12;PFL1505c;Pf_1015_1015	1.5265219	Up	-2.3088913	Down	-1.2831457	Down
Pfa3D7;chr14;PF14_0123;Pf_9560_9560	1.9153384	Up	-2.1919758	Down	-1.2892187	Down
Pfa3D7;chr8;PF08_0073;Pf_1120_1120	1.5687605	Up	-3.0573735	Down	-1.7617892	Down
Pfa3D7;chr14;PF14_0604;Pf_639_639	3.2176135	Up	-1.6307794	Down	1.9108385	Up
Pfa3D7;chr13;MAL13P1.245;Pf_2041_2041	-1.453476	Down	-1.6293346	Down	-2.2409256	Down
Pfa3D7;chr10;PF10_0290;Pf_1987_1987	1.6226395	Up	-4.7676497	Down	-2.9700522	Down
Pfa3D7;pfal_chr2;PFB0870w;Pf_6943_6943	-3.2526383	Down	3.2288299	Up	1.0244713	Up
Pfa3D7;chr13;MAL13P1.295;Pf_5922_5922	-3.1209555	Down	3.5928621	Up	1.1611558	Up

	[UT8] vs [UT16]		[UT16] vs [T16]		[UT8] vs [T16]	
**Accession number**	**Fold-change**	**Regulation**	**Fold-change**	**Regulation**	**Fold-change**	**Regulation**
Pfa3D7;chr6;PFF0470w;Pf_3051_3051	-1.1339121	Down	-2.3466141	Down	-3.172841	Down
Pfa3D7;pfal_chr5;PFE0510c;Pf_461_461	1.0197475	Up	-2.038583	Down	-1.7200577	Down
Pfa3D7;pfal_chr5;PFE0360c;Pf_2225_2225	1.8123494	Up	-2.308158	Down	-1.2351495	Down
Pfa3D7;chr6;PFF1075w;Pf_2360_2360	1.3064096	Up	-2.3468223	Down	-2.05641	Down
Pfa3D7;pfal_chr1;PFA0395c;Pf_536_536	-2.125153	Down	1.4563439	Up	-1.5392894	Down
Pfa3D7;chr14;PF14_0480;Pf_4788_4788	1.3239088	Up	-2.165703	Down	-1.7515129	Down
Pfa3D7;chr13;MAL13P1.150;Pf_1401_1401	-1.9512608	Down	2.128485	Up	1.0743003	Up
Pfa3D7;chr6;PFF1045w;Pf_4679_4679	-2.0493627	Down	1.9200045	Up	-1.0515796	Down
Pfa3D7;pfal_chr5;PFE0950c;Pf_441_441	1.2725631	Up	-2.5564165	Down	-1.979042	Down
Pfa3D7;chr12;PFL2505c;Pf_6559_6559	2.0831475	Up	1.2483258	Up	2.712758	Up
Pfa3D7;chr14;PF14_0204;Pf_3702_3702	2.5193393	Up	-1.5930934	Down	1.6027039	Up
Pfa3D7;chr14;PF14_0705;Pf_93_93	-2.235142	Down	3.057183	Up	1.3151298	Up
Pfa3D7;pfal_chr3;PFC0335c;Pf_10822_10822	-2.2458634	Down	1.8171453	Up	-1.1738143	Down
Pfa3D7;pfal_chr2;PFB0485c;Pf_1095_1095	2.3034587	Up	-1.7295959	Down	1.316137	Up
Pfa3D7;pfal_chr2;PFB0475c;Pf_1135_1135	1.7133087	Up	-2.02823	Down	1.0010424	Up
Pfa3D7;pfal_chr5;PFE0360c;Pf_2305_2305	1.7565185	Up	-2.2246747	Down	-1.1336026	Down
Pfa3D7;chr12;PFL1750c;Pf_4236_4236	1.2022638	Up	-2.3425746	Down	-1.934091	Down
Pfa3D7;chr10;PF10_0065;Pf_765_765	-2.3950076	Down	1.8266475	Up	-1.4181448	Down
Pfa3D7;chr11;PF11_0413;Pf_859_859	-3.3845735	Down	3.016375	Up	1.0345641	Up
Pfa3D7;pfal_chr9;PFI0915w;Pf_2107_2107	5.414765	Up	-2.8087332	Down	2.0203655	Up
Pfa3D7;pfal_chr4;PFD0345c;Pf_1083_1083	2.2047071	Up	-1.459738	Down	1.3567246	Up
Pfa3D7;chr8;PF08_0058;Pf_2748_2748	1.5788448	Up	-2.2434826	Down	-1.264152	Down
Pfa3D7;chr13;PF13_0281;Pf_320_320	-3.487465	Down	3.510305	Up	-1.0009038	Down
Pfa3D7;chr13;MAL13P1.201;Pf_1661_1661	-2.2315667	Down	3.2112892	Up	1.2284651	Up
Pfa3D7;pfal_chr9;PFI0445c;Pf_1337_1337	1.361759	Up	-2.3442857	Down	-1.8419223	Down
Pfa3D7;chr13;MAL13P1.24;Pf_546_546	1.4412998	Up	-2.3808703	Down	-1.7222435	Down
Pfa3D7;chr13;MAL13P1.342;Pf_1666_1666	-1.0174838	Down	-1.8935769	Down	-2.3009746	Down
Pfa3D7;chr13;PF13_0060;Pf_1411_1411	2.5170603	Up	-3.0885544	Down	-1.388806	Down
Pfa3D7;pfal_chr9;PFI0795w;Pf_3237_3237	-2.883798	Down	1.9637502	Up	-1.5416449	Down
Pfa3D7;chr12;PFL0260c;Pf_835_835	1.3307353	Up	-2.0683992	Down	-1.394638	Down
Pfa3D7;pfal_chr2;PFB0235w;Pf_1737_1737	1.2580305	Up	-2.5833943	Down	-2.088197	Down
Pfa3D7;chr10;PF10_0292;Pf_4760_4760	1.4009894	Up	-2.06996	Down	-1.3364611	Down
Pfa3D7;chr10;PF10_0060;Pf_1826_1826	1.3254702	Up	-2.072438	Down	-1.5459478	Down
Pfa3D7;chr8;MAL8P1.45;Pf_2463_2463	1.4922093	Up	-2.2455125	Down	-1.5461406	Down
Pfa3D7;pfal_chr5;PFE1000c;Pf_1518_1518	-2.359844	Down	1.5486403	Up	-1.6016287	Down
Pfa3D7;chr14;PF14_0367;Pf_1939_1939	1.1308471	Up	-2.3092177	Down	-2.091067	Down
Pfa3D7;chr12;PFL2120w;Pf_3795_3795	-2.0678117	Down	1.5464563	Up	-1.325372	Down
Pfa3D7;pfal_chr4;PFD0375w;Pf_3538_3538	-1.1810561	Down	-2.3587506	Down	-2.542639	Down
Pfa3D7;chr13;PF13_0020;Pf_1777_1777	1.4695975	Up	-2.0860112	Down	-1.501123	Down
Pfa3D7;pfal_chr9;PFI0670w;Pf_1773_1773	-2.1578004	Down	1.6976862	Up	-1.3098756	Down
Pfa3D7;chr11;PF11_0379;Pf_1202_1202	2.5271354	Up	-2.293515	Down	1.1053368	Up
Pfa3D7;chr7;PF07_0114;Pf_2551_2551	-2.167884	Down	1.8309121	Up	-1.2489637	Down
Pfa3D7;pfal_chr5;PFE0575c;Pf_407_407	1.5508038	Up	1.4893085	Up	2.6212122	Up
Pfa3D7;chr14;PF14_0338;Pf_2643_2643	-2.0338137	Down	1.6385677	Up	-1.1664828	Down
Pfa3D7;chr13;MAL13P1.180;Pf_771_771	-2.2920616	Down	2.1656096	Up	-1.124952	Down
Pfa3D7;pfal_chr9;PFI1410c;Pf_4104_4104	-2.145148	Down	2.1933796	Up	-1.0501908	Down
Pfa3D7;chr14;PF14_0385;Pf_978_978	1.0757954	Up	-2.0481124	Down	-1.850647	Down
Pfa3D7;chr14;PF14_0674;Pf_1681_1681	-2.488227	Down	2.0402384	Up	-1.142923	Down
Pfa3D7;chr14;PF14_0574;Pf_893_893	2.075257	Up	-2.1005125	Down	1.0242848	Up
Pfa3D7;chr12;PFL1300c;Pf_1221_1221	-2.651945	Down	2.3902016	Up	1.0158886	Up
Pfa3D7;chr13;PF13_0162;Pf_5282_5282	2.7846828	Up	-2.1253338	Down	1.3414156	Up
Pfa3D7;chr12;PFL1460c;Pf_632_632	-1.1123654	Down	-2.3119462	Down	-2.2737331	Down
Pfa3D7;chr13;PF13_0220;Pf_1098_1098	-1.6392303	Down	-2.0629098	Down	-3.2390532	Down
Pfa3D7;chr14;PF14_0556;Pf_3939_3939	2.003999	Up	-1.3465433	Down	1.4798154	Up
Pfa3D7;chr14;PF14_0567;Pf_964_964	2.013246	Up	-1.7011122	Down	1.1048726	Up
Pfa3D7;chr13;MAL13P1.138;Pf_1904_1904	-1.4913418	Down	2.0064967	Up	1.5075927	Up

	[UT8] vs [UT16]		[UT16] vs [T16]		[UT8] vs [T16]	
**Accession number**	**Fold-change**	**Regulation**	**Fold-change**	**Regulation**	**Fold-change**	**Regulation**
Pfa3D7;chr12;PFL2450c;Pf_184_184	-1.7357284	Down	-2.5045135	Down	-3.5661173	Down
Pfa3D7;pfal_chr2;PFB0205c;Pf_3348_3348	-2.6033764	Down	2.5140395	Up	-1.0815765	Down
Pfa3D7;pfal_chr3;PFC0370w;Pf_1310_1310	1.4458978	Up	-2.1024404	Down	-1.3287549	Down
Pfa3D7;pfal_chr9;PFI1035w;Pf_519_519	-2.5109115	Down	1.9791793	Up	-1.3624438	Down
Pfa3D7;chr10;PF10_0233;Pf_1580_1580	-3.6503048	Down	3.7899323	Up	1.126109	Up
Pfa3D7;chr14;PF14_0668;Pf_6927_6927	-1.9150593	Down	2.4585607	Up	1.2727687	Up
Pfa3D7;chr13;PF13_0307;Pf_3269_3269	-1.8448275	Down	2.0592709	Up	1.0456463	Up
Pfa3D7;chr12;PFL2220w;Pf_2602_2602	2.0525985	Up	-2.2338526	Down	-1.1344739	Down
Pfa3D7;pfal_chr3;PFC0620w;Pf_1617_1617	1.4129668	Up	-3.4813683	Down	-2.6225712	Down
Pfa3D7;pfal_chr1;PFA0210c;Pf_1200_1200	-1.78602	Down	2.573638	Up	1.6900313	Up
Pfa3D7;pfal_chr5;PFE1200w;Pf_3623_3623	2.1799974	Up	-1.4882673	Down	1.2938886	Up
Pfa3D7;chr11;PF11_0231;Pf_781_781	1.7283945	Up	-2.0769548	Down	-1.1657658	Down
Pfa3D7;chr14;PF14_0574;Pf_853_853	2.180883	Up	-2.0946236	Down	1.0643551	Up
Pfa3D7;chr6;PFF0280w;Pf_697_697	-1.1094933	Down	-2.7515697	Down	-3.4417837	Down
Pfa3D7;chr13;PF13_0192;Pf_1839_1839	-2.3078833	Down	4.931361	Up	1.9490895	Up
Pfa3D7;chr11;PF11_0347;Pf_2935_2935	3.0686858	Up	-1.9175947	Down	1.5745515	Up
Pfa3D7;pfal_chr2;PFB0745w;Pf_3960_3960	-1.0875136	Down	2.2082977	Up	2.0336215	Up
Pfa3D7;pfal_chr5;PFE1025c;Pf_845_845	-1.466461	Down	2.146791	Up	1.3575158	Up
Pfa3D7;pfal_chr2;PFB0745w;Pf_3872_3872	-1.0480311	Down	2.0971239	Up	1.9286996	Up
Pfa3D7;chr6;PFF0405c;Pf_495_495	-2.1488671	Down	3.4136817	Up	1.6104767	Up
Pfa3D7;pfal_chr9;PFI1265w;Pf_4973_4973	-2.4035482	Down	2.0670967	Up	-1.1228312	Down
Pfa3D7;chr11;PF11_0073;Pf_1878_1878	-2.2720525	Down	2.4630928	Up	1.0941311	Up
Pfa3D7;pfal_chr3;PFC0370w;Pf_1247_1247	1.365811	Up	-2.1892424	Down	-1.411643	Down
Pfa3D7;chr12;PFL1195w;Pf_901_901	3.324977	Up	-2.241224	Down	1.3746378	Up
Pfa3D7;chr13;MAL13P1.268;Pf_1558_1558	8.198259	Up	-2.8800545	Down	2.7292273	Up
Pfa3D7;chr6;PFF0130c;Pf_1262_1262	2.0122857	Up	-2.205031	Down	-1.1270123	Down
Pfa3D7;pfal_chr4;PFD0695w;Pf_724_724	1.1781875	Up	-2.6530912	Down	-2.8068068	Down
Pfa3D7;chr8;MAL8P1.95;Pf_781_781	-1.4678305	Down	2.5537794	Up	1.7525499	Up
Pfa3D7;chr14;PF14_0204;Pf_3499_3499	2.9899666	Up	-1.7385557	Down	1.7279221	Up
Pfa3D7;pfal_chr5;PFE1375c;Pf_1210_1210	-1.5440308	Down	-1.4967217	Down	-2.1718602	Down
Pfa3D7;chr13;MAL13P1.150;Pf_1361_1361	-2.075837	Down	2.2474072	Up	-1.0118394	Down
Pfa3D7;chr14;PF14_0074;Pf_1094_1094	2.119543	Up	-3.7081258	Down	-1.6840477	Down
Pfa3D7;chr14;PF14_0319;Pf_7767_7767	-2.012649	Down	2.1778588	Up	1.0327502	Up
Pfa3D7;chr12;PFL0895c;Pf_2915_2915	2.2395382	Up	-1.9199349	Down	1.1687027	Up
Pfa3D7;chr7;MAL7P1.77;Pf_1491_1491	-2.2289839	Down	3.901374	Up	1.6700565	Up
Pfa3D7;pfal_chr3;PFC0910w;Pf_1143_1143	-5.8945646	Down	2.4085102	Up	-2.3159046	Down
Pfa3D7;chr14;PF14_0318;Pf_5876_5876	-2.1444314	Down	2.6628892	Up	1.2026505	Up
Pfa3D7;pfal_chr9;PFI0270w;Pf_633_633	1.9054562	Up	-2.0105422	Down	1.0191371	Up
Pfa3D7;chr10;PF10_0338;Pf_318_318	-2.6767695	Down	2.179858	Up	-1.2207316	Down
Pfa3D7;chr14;PF14_0693;Pf_444_444	-1.0904939	Down	-1.8939296	Down	-2.0015638	Down
Pfa3D7;chr8;PF08_0082;Pf_1796_1796	1.5226419	Up	-2.0834534	Down	-1.377953	Down
Pfa3D7;chr13;MAL13P1.114;Pf_10267_10267	2.239639	Up	-1.6781163	Down	1.3560454	Up
Pfa3D7;pfal_chr3;PFC0910w;Pf_1185_1185	-6.155904	Down	2.376356	Up	-2.4269462	Down
Pfa3D7;chr10;PF10_0152;Pf_1681_1681	-3.8390927	Down	2.5692847	Up	-1.3826755	Down
Pfa3D7;chr6;PFF0130c;Pf_1208_1208	2.2339737	Up	-2.224762	Down	-1.1100858	Down
Pfa3D7;pfal_chr2;PFB0256w;Pf_43_43	2.479601	Up	-1.4361415	Down	1.6117946	Up
Pfa3D7;chr13;PF13_0020;Pf_1737_1737	1.1254805	Up	-2.04868	Down	-1.8393741	Down
Pfa3D7;chr12;PFL0280c;Pf_824_824	-2.1764357	Down	2.1568553	Up	-1.0107249	Down
Pfa3D7;chr10;PF10_0307;Pf_2524_2524	-3.024696	Down	1.3854867	Up	-1.9720818	Down
Pfa3D7;pfal_chr2;PFB0115w;Pf_3476_3476	-15.588168	Down	4.8041644	Up	-2.7407916	Down
Pfa3D7;chr14;PF14_0152;Pf_4148_4148	-2.86094	Down	1.91302	Up	-1.324698	Down
Pfa3D7;pfal_chr2;PFB0485c;Pf_1055_1055	2.4100804	Up	-1.8190596	Down	1.2275	Up
Pfa3D7;chr8;MAL8P1.62;Pf_513_513	-2.179053	Down	1.8871645	Up	-1.0343457	Down
Pfa3D7;chr11;PF11_0346;Pf_1726_1726	2.4946024	Up	-2.1557558	Down	1.1227518	Up
Pfa3D7;chr10;PF10_0265;Pf_5330_5330	1.1378089	Up	-2.1352613	Down	-1.9801679	Down
Pfa3D7;chr6;PFF1045w;Pf_4565_4565	-2.3128693	Down	1.9654828	Up	-1.1687198	Down

	[UT8] vs [UT16]		[UT16] vs [T16]		[UT8] vs [T16]	
**Accession number**	**Fold-change**	**Regulation**	**Fold-change**	**Regulation**	**Fold-change**	**Regulation**
Pfa3D7;chr6;PFF0480w;Pf_5605_5605	2.1094427	Up	-2.1983774	Down	-1.1068486	Down
Pfa3D7;chr13;MAL13P1.138;Pf_1944_1944	-1.6344	Down	2.9162056	Up	1.561242	Up
Pfa3D7;pfal_chr5;PFE0875c;Pf_2388_2388	-1.1583172	Down	-1.9861207	Down	-2.1932018	Down
Pfa3D7;pfal_chr5;PFE0190c;Pf_1903_1903	-2.3047104	Down	2.5632048	Up	1.0356302	Up
Pfa3D7;chr14;PF14_0310;Pf_2112_2112	-2.325782	Down	1.7025963	Up	-1.3588988	Down
Pfa3D7;pfal_chr4;PFD0550c;Pf_402_402	-2.2344782	Down	2.029834	Up	-1.1775949	Down
Pfa3D7;chr8;PF08_0083;Pf_1620_1620	2.055147	Up	-1.8739142	Down	1.1189641	Up
Pfa3D7;chr11;PF11_0056;Pf_1842_1842	-1.2411649	Down	-1.8102573	Down	-2.185566	Down
Pfa3D7;chr14;PF14_0037;Pf_366_366	-2.502586	Down	2.274246	Up	-1.0257112	Down
Pfa3D7;chr12;PFL0825c;Pf_659_659	1.1333438	Up	-2.0592103	Down	-1.819696	Down
Pfa3D7;chr13;MAL13P1.15;Pf_177_177	-2.3781395	Down	2.501529	Up	1.1382478	Up
Pfa3D7;chr11;PF11_0193;Pf_687_687	1.0857314	Up	-2.065223	Down	-1.6326104	Down
Pfa3D7;pfal_chr4;PFD0555c;Pf_3637_3637	-2.9490793	Down	2.0055792	Up	-1.4333793	Down
Pfa3D7;chr11;PF11_0460;Pf_1712_1712	-1.1300702	Down	-2.4441464	Down	-2.4093862	Down
Pfa3D7;chr13;PF13_0266;Pf_582_582	-1.70597	Down	-1.7940912	Down	-2.6438239	Down
Pfa3D7;chr8;MAL8P1.62;Pf_473_473	-2.3662481	Down	1.8157268	Up	-1.1082267	Down
Pfa3D7;chr13;MAL13P1.306;Pf_890_890	2.031788	Up	-1.4571385	Down	1.5413362	Up
Pfa3D7;pfal_chr5;PFE1025c;Pf_805_805	-1.5769109	Down	2.1905832	Up	1.3012533	Up
Pfa3D7;chr8;MAL8P1.107;Pf_327_327	-4.861114	Down	3.0664113	Up	-1.4047272	Down
Pfa3D7;chr13;MAL13P1.268;Pf_1458_1458	7.9734416	Up	-2.8630388	Down	2.6858702	Up
Pfa3D7;chr13;PF13_0164;Pf_2067_2067	1.0280595	Up	-2.0330067	Down	-2.0276618	Down
Pfa3D7;chr13;MAL13P1.295;Pf_5862_5862	-3.541323	Down	3.6774232	Up	1.0797532	Up
Pfa3D7;chr8;MAL8P1.100;Pf_1362_1362	1.2826602	Up	-2.2623885	Down	-1.9587573	Down
Pfa3D7;chr14;PF14_0168;Pf_982_982	-1.2650903	Down	-2.5769913	Down	-3.0317621	Down
Pfa3D7;chr11;PF11_0413;Pf_900_900	-3.4788527	Down	2.9707983	Up	-1.1292884	Down
Pfa3D7;chr14;PF14_0728;Pf_30_30	2.063737	Up	-1.2725619	Down	1.5449829	Up
Pfa3D7;chr13;PF13_0210;Pf_9546_9546	1.2167625	Up	1.8727623	Up	2.1834102	Up
Pfa3D7;pfal_chr5;PFE1480c;Pf_2849_2849	-2.3270223	Down	1.7882396	Up	-1.330194	Down
Pfa3D7;chr13;PF13_0267;Pf_1431_1431	-1.4975029	Down	-2.2365658	Down	-3.5841815	Down
Pfa3D7;chr7;MAL7P1.76;Pf_2238_2238	1.7508675	Up	-2.529043	Down	-1.4171363	Down
Pfa3D7;pfal_chr5;PFE0575c;Pf_492_492	1.8462582	Up	1.3760643	Up	2.630593	Up
Pfa3D7;pfal_chr4;PFD0435c;Pf_622_622	-1.0306196	Down	-6.5201354	Down	-7.231573	Down
Pfa3D7;pfal_chr3;PFC1025w;Pf_53_53	-1.6852086	Down	2.1137938	Up	1.3088828	Up
Pfa3D7;pfal_chr1;PFA0570w;Pf_1733_1733	-2.8696988	Down	1.9350348	Up	-1.4285156	Down
Pfa3D7;chr14;PF14_0367;Pf_2019_2019	-1.0174985	Down	-2.3456755	Down	-2.2010825	Down
Pfa3D7;chr8;MAL8P1.77;Pf_327_327	-2.2951832	Down	1.8741295	Up	-1.2140396	Down
Pfa3D7;chr14;PF14_0319;Pf_7860_7860	-2.0813417	Down	2.168756	Up	1.1340901	Up
Pfa3D7;chr14;PF14_0544;Pf_1955_1955	-3.8385346	Down	1.956998	Up	-1.6201093	Down
Pfa3D7;chr6;PFF0705c;Pf_1247_1247	1.3008639	Up	-2.1062555	Down	-1.507767	Down
Pfa3D7;chr12;PFL0700w;Pf_440_440	-1.4903053	Down	-1.7503757	Down	-2.344701	Down
Pfa3D7;chr7;PF07_0055;Pf_1986_1986	1.0048364	Up	-2.0575166	Down	-2.0657897	Down
Pfa3D7;pfal_chr9;PFI0915w;Pf_2027_2027	6.0203905	Up	-2.7883196	Down	2.2352736	Up
Pfa3D7;chr14;PF14_0412;Pf_2341_2341	-2.0715125	Down	2.2688704	Up	1.0524262	Up
Pfa3D7;chr7;PF07_0087;Pf_437_437	-2.608415	Down	6.2871003	Up	2.4342906	Up
Pfa3D7;pfal_chr9;PFI0140w;Pf_187_187	5.027686	Up	-2.7643745	Down	1.838805	Up
Pfa3D7;pfal_chr9;PFI0800c;Pf_87_87	2.7102706	Up	-2.644934	Down	1.1585749	Up
Pfa3D7;pfal_chr9;PFI1265w;Pf_5013_5013	-2.1956697	Down	1.8960698	Up	-1.0039716	Down
Pfa3D7;pfal_chr3;PFC0215c;Pf_1254_1254	1.1147405	Up	-3.2480285	Down	-2.5391889	Down
Pfa3D7;chr12;PFL2220w;Pf_2504_2504	2.0803833	Up	-2.1871085	Down	-1.0937746	Down
Pfa3D7;pfal_chr9;PFI0795w;Pf_3337_3337	-2.7509863	Down	1.9969801	Up	-1.4524794	Down
Pfa3D7;chr8;PF08_0073;Pf_717_717	-1.5142998	Down	-3.0996578	Down	-4.0258875	Down
Pfa3D7;chr13;MAL13P1.201;Pf_1701_1701	-2.0983756	Down	2.2877488	Up	1.0063	Up
Pfa3D7;pfal_chr9;PFI0825w;Pf_747_747	2.3493624	Up	-1.6329323	Down	1.4024581	Up
Pfa3D7;chr12;PFL0485w;Pf_531_531	-2.193234	Down	2.128111	Up	-1.1022815	Down
Pfa3D7;pfal_chr5;PFE0320w;Pf_2672_2672	-2.2410514	Down	1.5399895	Up	-1.4094402	Down
Pfa3D7;chr12;PFL2130w;Pf_3929_3929	-1.9135183	Down	2.2054274	Up	1.2653035	Up

	[UT8] vs [UT16]		[UT16] vs [T16]		[UT8] vs [T16]	
**Accession number**	**Fold-change**	**Regulation**	**Fold-change**	**Regulation**	**Fold-change**	**Regulation**
Pfa3D7;pfal_chr5;PFE1245w;Pf_1442_1442	-3.222364	Down	2.327803	Up	-1.2770815	Down
Pfa3D7;chr11;PF11_0166;Pf_604_604	2.175965	Up	-1.6188684	Down	1.4096932	Up
Pfa3D7;chr8;MAL8P1.15;Pf_3034_3034	-3.7345593	Down	3.4003496	Up	1.0178066	Up
Pfa3D7;chr11;PF11_0135;Pf_279_279	1.0324565	Up	-2.1077554	Down	-1.8166064	Down
Pfa3D7;pfal_chr3;PFC0335c;Pf_11048_11048	-2.0174298	Down	1.6640418	Up	-1.2535344	Down
Pfa3D7;chr7;PF07_0041;Pf_1292_1292	2.2209904	Up	-1.9958684	Down	1.1155202	Up
Pfa3D7;chr12;PFL1195w;Pf_723_723	3.4434686	Up	-2.062609	Down	1.5078741	Up
Pfa3D7;chr7;MAL7P1.117;Pf_115_115	-1.2951043	Down	2.0388384	Up	1.4870093	Up
Pfa3D7;pfal_chr3;PFC0695w;Pf_701_701	2.9368665	Up	-3.5259972	Down	-1.1991571	Down
Pfa3D7;chr14;PF14_0674;Pf_1498_1498	-2.209476	Down	1.9824687	Up	-1.1829194	Down
Pfa3D7;chr6;PFF1195c;Pf_1791_1791	1.0043459	Up	-2.0370216	Down	-1.9277321	Down
Pfa3D7;chr12;PFL0280c;Pf_904_904	-1.9616483	Down	2.1163301	Up	1.1017238	Up
Pfa3D7;pfal_chr2;PFB0670c;Pf_745_745	1.4939066	Up	-2.3126504	Down	-1.3747518	Down
Pfa3D7;chr14;PF14_0623;Pf_613_613	1.2971467	Up	-2.0200427	Down	-1.6355338	Down
Pfa3D7;chr13;PF13_0162;Pf_5462_5462	2.6849582	Up	-2.153844	Down	1.2481122	Up
Pfa3D7;chr13;PF13_0217;Pf_71_71	-2.0654166	Down	1.6773208	Up	-1.1292648	Down
Pfa3D7;chr14;PF14_0402;Pf_5463_5463	-4.2810955	Down	2.663529	Up	-1.6615108	Down
Pfa3D7;pfal_chr1;PFA0255c;Pf_1517_1517	-3.2488947	Down	2.4893172	Up	-1.3755282	Down
Pfa3D7;chr8;MAL8P1.74;Pf_2018_2018	-2.410055	Down	1.4014097	Up	-1.6707544	Down
Pfa3D7;chr13;MAL13P1.180;Pf_820_820	-2.2032497	Down	2.0702062	Up	-1.1075369	Down
Pfa3D7;chr11;PF11_0166;Pf_775_775	3.4010103	Up	-1.9491068	Down	1.7606803	Up
Pfa3D7;chr13;MAL13P1.188;Pf_1726_1726	1.1792319	Up	-2.2170558	Down	-2.0085113	Down
Pfa3D7;chr14;PF14_0080;Pf_238_238	1.0416987	Up	-2.7134123	Down	-2.595463	Down
Pfa3D7;pfal_chr1;PFA0255c;Pf_1637_1637	-2.761186	Down	2.4533143	Up	-1.1685779	Down
Pfa3D7;pfal_chr4;PFD1130w;Pf_957_957	1.3985918	Up	1.3908315	Up	2.3919344	Up
Pfa3D7;chr6;PFF0490w;Pf_5992_5992	2.638169	Up	-1.8035185	Down	1.2809546	Up
Pfa3D7;chr10;PF10_0265;Pf_5370_5370	1.2987314	Up	-2.1977036	Down	-2.0479448	Down
Pfa3D7;chr14;PF14_0279;Pf_690_690	-2.605408	Down	2.3317027	Up	-1.1496888	Down
Pfa3D7;chr14;PF14_0604;Pf_691_691	2.5686777	Up	-1.7431523	Down	1.4472121	Up
Pfa3D7;chr11;PF11_0482;Pf_3956_3956	-1.4346126	Down	2.0522795	Up	1.385855	Up
Pfa3D7;chr14;PF14_0168;Pf_475_475	-1.2972084	Down	-2.600482	Down	-3.1694489	Down
Pfa3D7;pfal_chr9;PFI1230c;Pf_969_969	-1.7377114	Down	-1.7926673	Down	-2.9994354	Down
Pfa3D7;pfal_chr5;PFE1200w;Pf_3540_3540	2.294894	Up	-1.4959569	Down	1.3626685	Up
Pfa3D7;chr12;PFL1415w;Pf_2788_2788	-3.1323504	Down	1.6370821	Up	-1.7872833	Down
Pfa3D7;pfal_chr4;PFD0205c;Pf_416_416	-1.4367985	Down	-1.5266703	Down	-2.033776	Down
Pfa3D7;pfal_chr4;PFD0545w;Pf_4537_4537	-1.363121	Down	-1.5841752	Down	-2.2073922	Down
Pfa3D7;chr10;PF10_0258;Pf_428_428	-1.417901	Down	-1.7613934	Down	-2.2332287	Down
Pfa3D7;pfal_chr5;PFE0510c;Pf_625_625	1.0267158	Up	-2.069968	Down	-1.8241494	Down
Pfa3D7;chr8;PF08_0083;Pf_1741_1741	2.3719785	Up	-1.9893543	Down	1.2319506	Up
Pfa3D7;chr12;PFL0895c;Pf_2869_2869	2.1433175	Up	-1.885732	Down	1.1462016	Up
Pfa3D7;pfal_chr4;PFD0545w;Pf_4497_4497	-1.3505797	Down	-1.6027428	Down	-2.2505116	Down
Pfa3D7;chr7;MAL7P1.22;Pf_4541_4541	3.221092	Up	-1.6859324	Down	1.8862143	Up
Pfa3D7;chr13;PF13_0337;Pf_388_388	-1.4069293	Down	2.6926641	Up	1.9074416	Up
Pfa3D7;pfal_chr9;PFI1410c;Pf_4426_4426	-2.0966005	Down	1.9709505	Up	-1.0810627	Down
Pfa3D7;chr12;PFL0065w;Pf_61_61	2.1335182	Up	-1.5712672	Down	1.2849151	Up
Pfa3D7;chr10;PF10_0065;Pf_884_884	-2.331653	Down	1.6235957	Up	-1.4818165	Down
Pfa3D7;chr10;PF10_0101;Pf_208_208	-1.2055795	Down	-2.3139017	Down	-2.5174336	Down
Pfa3D7;chr14;PF14_0412;Pf_2385_2385	-2.4158878	Down	2.3605886	Up	-1.097064	Down
Pfa3D7;chr13;PF13_0267;Pf_1493_1493	-1.5632608	Down	-2.489122	Down	-3.9149334	Down
Pfa3D7;chr12;PFL1165w;Pf_2338_2338	-1.5550948	Down	-1.9877125	Down	-2.7380922	Down
Pfa3D7;chr6;PFF1075w;Pf_2293_2293	1.1157179	Up	-2.4217286	Down	-2.4284573	Down
Pfa3D7;pfal_chr4;PFD0495c;Pf_2442_2442	-1.1640774	Down	-1.7016495	Down	-2.0927522	Down
Pfa3D7;chr8;MAL8P1.52;Pf_296_296	2.3819745	Up	-1.9426471	Down	1.3425071	Up
Pfa3D7;chr12;PFL1460c;Pf_672_672	-1.0334198	Down	-2.336069	Down	-2.1685383	Down
Pfa3D7;chr11;PF11_0346;Pf_2052_2052	2.2077143	Up	-2.119319	Down	1.0638466	Up
Pfa3D7;chr7;MAL7P1.152;Pf_2865_2865	-2.0496278	Down	2.3233387	Up	1.2232989	Up

	[UT8] vs [UT16]		[UT16] vs [T16]		[UT8] vs [T16]	
**Accession number**	**Fold-change**	**Regulation**	**Fold-change**	**Regulation**	**Fold-change**	**Regulation**
Pfa3D7;chr8;MAL8P1.88;Pf_2214_2214	4.0434527	Up	-2.1902146	Down	1.9188588	Up
Pfa3D7;pfal_chr5;PFE1500c;Pf_121_121	2.6888483	Up	1.5649549	Up	3.9936745	Up
Pfa3D7;pfal_chr5;PFE0640w;Pf_732_732	-2.7461739	Down	2.1019063	Up	-1.4756112	Down
Pfa3D7;pfal_chr1;PFA0210c;Pf_1240_1240	-1.8484408	Down	2.5631778	Up	1.5581613	Up
Pfa3D7;chr14;PF14_0329;Pf_587_587	-5.834456	Down	6.6183753	Up	1.1560472	Up
Pfa3D7;chr14;PF14_0257;Pf_463_463	-1.541192	Down	2.016385	Up	1.274894	Up
Pfa3D7;chr13;PF13_0106;Pf_2315_2315	2.4223278	Up	-2.5743167	Down	-1.0386572	Down
Pfa3D7;chr14;PF14_0329;Pf_651_651	-5.4694853	Down	6.3797636	Up	1.1888813	Up
Pfa3D7;chr13;PF13_0106;Pf_2275_2275	2.5431354	Up	-2.7191415	Down	-1.060926	Down
Pfa3D7;pfal_chr9;PFI0145w;Pf_385_385	-2.9660938	Down	1.5981934	Up	-1.7887747	Down
Pfa3D7;chr14;PF14_0266;Pf_1126_1126	-2.2039232	Down	1.6729596	Up	-1.3543468	Down
Pfa3D7;chr8;MAL8P1.63;Pf_938_938	-4.318803	Down	3.1286163	Up	-1.4531368	Down
Pfa3D7;chr11;PF11_0045;Pf_484_484	-1.6848519	Down	-2.1416523	Down	-3.0562	Down
Pfa3D7;chr14;PF14_0272;Pf_346_346	-1.3918395	Down	-1.7980437	Down	-2.2422674	Down
Pfa3D7;pfal_chr3;PFC0640w;Pf_6182_6182	2.0621324	Up	-5.179657	Down	-2.4514685	Down
Pfa3D7;pfal_chr3;PFC0640w;Pf_6282_6282	2.2346437	Up	-5.784975	Down	-2.5282433	Down
Pfa3D7;pfal_chr3;PFC0640w;Pf_6222_6222	2.0749512	Up	-5.444317	Down	-2.5575488	Down
Pfa3D7;chr11;PF11_0165;Pf_1133_1133	-2.2298393	Down	2.8162205	Up	1.2385361	Up
Pfa3D7;chr11;PF11_0165;Pf_1383_1383	-2.3214276	Down	2.7440603	Up	1.1382866	Up
Pfa3D7;chr11;PF11_0161;Pf_1376_1376	-1.5826601	Down	2.0758932	Up	1.2373383	Up
Pfa3D7;chr11;PF11_0161;Pf_1133_1133	-1.9509599	Down	2.5976522	Up	1.3036641	Up
Pfa3D7;pfal_chr2;PFB0935w;Pf_4232_4232	-1.3256665	Down	2.2827911	Up	2.0030913	Up
Pfa3D7;chr12;PFL1185c;Pf_644_644	-2.4051533	Down	1.6649965	Up	-1.4938158	Down
Pfa3D7;chr14;PF14_0038;Pf_108_108	-2.1184213	Down	2.6415603	Up	1.3175099	Up
Pfa3D7;pfal_chr5;PFE1215c;Pf_1005_1005	1.5514197	Up	-2.0115106	Down	-1.2922666	Down
Pfa3D7;chr13;PF13_0077;Pf_2421_2421	-2.2454453	Down	2.1370697	Up	-1.0881188	Down
Pfa3D7;chr8;PF08_0095;Pf_2003_2003	-6.2010193	Down	5.191631	Up	1.0147704	Up
Pfa3D7;chr8;PF08_0095;Pf_1916_1916	-5.3048973	Down	4.812093	Up	-1.1040689	Down
Pfa3D7;chr13;MAL13P1.216;Pf_4272_4272	2.2720678	Up	-1.9615251	Down	1.127466	Up
Pfa3D7;chr13;MAL13P1.216;Pf_4192_4192	2.4578948	Up	-1.9349966	Down	1.1932123	Up
Pfa3D7;chr14;PF14_0051;Pf_4452_4452	-2.0883358	Down	2.4520335	Up	1.1458536	Up
Pfa3D7;chr14;PF14_0316;Pf_4286_4286	2.334916	Up	-1.9957529	Down	1.1462755	Up
Pfa3D7;chr11;PF11_0264;Pf_4344_4344	-2.4961867	Down	2.5939276	Up	-1.0169572	Down
Pfa3D7;chr11;PF11_0264;Pf_4141_4141	-2.3433352	Down	2.713921	Up	1.1901748	Up
Pfa3D7;chr13;MAL13P1.162;Pf_610_610	-1.967207	Down	2.0135102	Up	1.0324179	Up
Pfa3D7;pfal_chr2;PFB0085c;Pf_2591_2591	7.3279443	Up	-2.7194595	Down	2.5237875	Up
Pfa3D7;chr11;PF11_0513;Pf_1703_1703	2.3335238	Up	-1.5327783	Down	1.4170831	Up
Pfa3D7;chr12;PFL2550w;Pf_1353_1353	2.7382383	Up	-1.7997097	Down	1.4819663	Up
Pfa3D7;chr12;PFL2550w;Pf_1313_1313	2.789557	Up	-1.6831471	Down	1.6118995	Up
Pfa3D7;chr13;PF13_0336;Pf_2365_2365	-2.109816	Down	2.1797888	Up	-1.0404266	Down
Pfa3D7;chr13;PF13_0336;Pf_2405_2405	-2.2674	Down	2.124654	Up	-1.0266579	Down
Pfa3D7;chr12;PFL0960w;Pf_101_101	-1.9725038	Down	2.0677042	Up	1.0620286	Up
Pfa3D7;chr12;PFL0960w;Pf_529_529	-2.2174408	Down	1.8889225	Up	-1.1169364	Down
Pfa3D7;chr7;MAL7P1.162;Pf_14839_14839	3.213346	Up	-1.7572929	Down	1.637285	Up
Pfa3D7;chr7;MAL7P1.162;Pf_14703_14703	2.893372	Up	-1.6285889	Down	1.6140785	Up
Pfa3D7;chr12;PFL0115w;Pf_16866_16866	2.4430606	Up	-2.6221876	Down	-1.2066195	Down
Pfa3D7;chr7;MAL7P1.162;Pf_14743_14743	3.5176308	Up	-1.7477363	Down	1.6115867	Up
Pfa3D7;pfal_chr9;PFI1745c;Pf_446_446	-4.362375	Down	3.3389587	Up	-1.2716469	Down
Pfa3D7;chr10;PF10_0323;Pf_868_868	-2.59599	Down	1.4045733	Up	-1.855686	Down
Pfa3D7;chr12;PFL1945c;Pf_244_244	3.8784382	Up	-2.046163	Down	2.1405642	Up
Pfa3D7;chr8;MAL8P1.6;Pf_447_447	-1.2302786	Down	-1.7679882	Down	-2.013089	Down
Pfa3D7;pfal_chr3;PFC0305w;Pf_1467_1467	-1.0760006	Down	-2.1150222	Down	-2.1728995	Down
Pfa3D7;pfal_chr3;PFC0305w;Pf_1334_1334	-1.0675884	Down	-2.2932794	Down	-2.426778	Down
Pfa3D7;pfal_chr3;PFC0190c;Pf_1524_1524	1.456409	Up	-2.377899	Down	-1.6131833	Down
Pfa3D7;pfal_chr3;PFC0190c;Pf_1439_1439	1.3614023	Up	-2.1584685	Down	-1.5483562	Down
Pfa3D7;chr6;PFF0115c;Pf_2529_2529	-2.6839106	Down	3.2975821	Up	1.1754402	Up

	[UT8] vs [UT16]		[UT16] vs [T16]		[UT8] vs [T16]	
**Accession number**	**Fold-change**	**Regulation**	**Fold-change**	**Regulation**	**Fold-change**	**Regulation**
Pfa3D7;chr6;PFF0115c;Pf_2696_2696	-2.3485854	Down	3.1221585	Up	1.2122309	Up
Pfa3D7;chr6;PFF0715c;Pf_1099_1099	-2.6013732	Down	2.0277607	Up	-1.3484454	Down
Pfa3D7;chr6;PFF0715c;Pf_987_987	-2.8300743	Down	2.2365386	Up	-1.2692605	Down
Pfa3D7;chr13;MAL13P1.184;Pf_2795_2795	-3.1687078	Down	1.870337	Up	-1.8552924	Down
Pfa3D7;chr11;PF11_0098;Pf_293_293	-1.8621873	Down	2.4937134	Up	1.2840852	Up
Pfa3D7;chr11;PF11_0098;Pf_914_914	-1.7439514	Down	2.1868882	Up	1.2092475	Up
Pfa3D7;chr10;PF10_0155;Pf_1206_1206	-1.1060162	Down	3.0428264	Up	2.7478118	Up
Pfa3D7;chr10;PF10_0155;Pf_955_955	-1.1469488	Down	3.1755643	Up	2.7670572	Up
Pfa3D7;chr13;MAL13P1.60;Pf_3547_3547	2.369339	Up	1.8364629	Up	5.0063615	Up
Pfa3D7;chr13;MAL13P1.60;Pf_3200_3200	1.7954707	Up	2.1727617	Up	4.3819366	Up
Pfa3D7;pfal_chr4;PFD1155w;Pf_3459_3459	1.4645423	Up	2.2206564	Up	3.6178446	Up
Pfa3D7;chr7;PF07_0128;Pf_4252_4252	1.79797	Up	1.607259	Up	3.4613187	Up
Pfa3D7;pfal_chr1;PFA0125c;Pf_4438_4438	2.1486464	Up	1.5651716	Up	3.703746	Up
Pfa3D7;pfal_chr1;PFA0075w;Pf_933_933	-1.1156304	Down	2.2345183	Up	1.804963	Up
Pfa3D7;pfal_chr1;PFA0075w;Pf_558_558	-1.1563463	Down	2.069975	Up	1.7292386	Up
Pfa3D7;pfal_chr3;PFC0115c;Pf_3956_3956	1.8238199	Up	1.6647773	Up	2.8506098	Up
Pfa3D7;pfal_chr3;PFC0115c;Pf_4016_4016	1.6624634	Up	1.4522094	Up	2.290467	Up
Pfa3D7;pfal_chr5;PFE1640w;Pf_9222_9222	-1.0644897	Down	2.3340185	Up	1.9341693	Up
Pfa3D7;chr6;PFF0020c;Pf_3541_3541	2.0108361	Up	-1.4828721	Down	1.2834039	Up
Pfa3D7;pfal_chr2;PFB0010w;Pf_4994_4994	2.6815495	Up	-1.2718066	Down	1.9183333	Up
Pfa3D7;pfal_chr4;PFD0005w;Pf_7534_7534	2.4144099	Up	-1.2927706	Down	1.8630095	Up
Pfa3D7;pfal_chr4;PFD1245c;Pf_5871_5871	2.2163954	Up	-1.2010826	Down	1.8247327	Up
Pfa3D7;pfal_chr4;PFD1045c;Pf_12533_12533	-1.6222169	Down	2.5457757	Up	1.4489433	Up
Pfa3D7;pfal_chr4;PFD1045c;Pf_12699_12699	-1.5734673	Down	2.7440734	Up	1.6366116	Up
Pfa3D7;chr14;PF14_0423;Pf_4588_4588	2.4873092	Up	-2.3021703	Down	1.0233346	Up
Pfa3D7;chr11;PF11_0074;Pf_1818_1818	-2.3109093	Down	2.043737	Up	-1.1043706	Down
Pfa3D7;chr11;PF11_0074;Pf_2025_2025	-2.0641205	Down	1.9968199	Up	-1.0922766	Down
Pfa3D7;chr14;PF14_0530;Pf_5641_5641	2.4363816	Up	-1.6928264	Down	1.270513	Up
Pfa3D7;chr7;PF07_0085;Pf_1507_1507	-2.083428	Down	1.7569149	Up	-1.1373003	Down
Pfa3D7;chr12;PFL2275c;Pf_147_147	-3.033827	Down	3.6964948	Up	1.2481804	Up
Pfa3D7;chr12;PFL2275c;Pf_284_284	-2.87787	Down	3.5413632	Up	1.1962295	Up
Pfa3D7;pfal_chr4;PFD0420c;Pf_1587_1587	1.7181275	Up	-2.3347225	Down	-1.3304368	Down
Pfa3D7;pfal_chr4;PFD0420c;Pf_1734_1734	1.8070444	Up	-2.1932428	Down	-1.2448503	Down
Pfa3D7;chr11;PF11_0172;Pf_832_832	-2.1782582	Down	2.1455226	Up	1.0729681	Up
Pfa3D7;chr11;PF11_0172;Pf_516_516	-1.9150234	Down	2.0712724	Up	1.1148348	Up
Pfa3D7;chr10;PF10_0244;Pf_2667_2667	2.0625799	Up	-2.0396419	Down	1.0040864	Up
Pfa3D7;chr10;PF10_0244;Pf_2627_2627	2.2859776	Up	-1.9261738	Down	1.1332705	Up
Pfa3D7;chr14;PF14_0425;Pf_1213_1213	-2.2568364	Down	2.1968832	Up	1.0366657	Up
Pfa3D7;pfal_chr9;PFI1720w;Pf_1377_1377	3.380774	Up	-2.2191548	Down	1.5721244	Up
Pfa3D7;pfal_chr9;PFI1720w;Pf_1475_1475	3.5215569	Up	-2.6151934	Down	1.3742577	Up
Pfa3D7;chr6;PFF0120w;Pf_1065_1065	-2.0787024	Down	2.413769	Up	1.2486818	Up
Pfa3D7;chr6;PFF0120w;Pf_646_646	-2.448662	Down	2.9878502	Up	1.2146144	Up
Pfa3D7;chr10;PF10_0245;Pf_2158_2158	2.0055416	Up	-1.7715167	Down	1.1053897	Up
Pfa3D7;chr10;PF10_0245;Pf_2407_2407	2.1478782	Up	-1.7106487	Down	1.2186126	Up
Pfa3D7;chr14;PF14_0341;Pf_1586_1586	-2.2448442	Down	1.3401817	Up	-1.632759	Down
Pfa3D7;chr14;PF14_0341;Pf_1468_1468	-2.4840925	Down	1.8388393	Up	-1.3140577	Down
Pfa3D7;chr8;PF08_0132;Pf_4083_4083	-4.011878	Down	1.8460755	Up	-1.8876904	Down
Pfa3D7;chr8;PF08_0132;Pf_3487_3487	-4.954689	Down	2.431755	Up	-1.973668	Down
Pfa3D7;pfal_chr1;PFA0620c;Pf_1509_1509	-4.574273	Down	3.3883135	Up	-1.2795551	Down
Pfa3D7;pfal_chr1;PFA0620c;Pf_1427_1427	-4.791629	Down	3.658231	Up	-1.0366222	Down
Pfa3D7;pfal_chr9;PFI1110w;Pf_891_891	2.096548	Up	-2.6994312	Down	-1.2891341	Down
Pfa3D7;pfal_chr9;PFI1110w;Pf_1075_1075	2.071345	Up	-2.7003255	Down	-1.3038867	Down
Pfa3D7;pfal_chr3;PFC0271c;Pf_219_219	-1.8654232	Down	2.8350813	Up	1.5572535	Up
Pfa3D7;chr14;PF14_0192;Pf_1372_1372	-2.0059118	Down	1.812157	Up	-1.060608	Down
Pfa3D7;chr14;PF14_0187;Pf_485_485	1.2260382	Up	1.9804765	Up	2.2901328	Up
Pfa3D7;chr12;PFL0780w;Pf_803_803	-2.316131	Down	2.6371226	Up	1.1834137	Up

	[UT8] vs [UT16]		[UT16] vs [T16]		[UT8] vs [T16]	
**Accession number**	**Fold-change**	**Regulation**	**Fold-change**	**Regulation**	**Fold-change**	**Regulation**
Pfa3D7;chr12;PFL0780w;Pf_588_588	-2.3554862	Down	3.0922844	Up	1.2822382	Up
Pfa3D7;chr13;PF13_0345;Pf_1135_1135	-2.3178017	Down	1.6038543	Up	-1.3933269	Down
Pfa3D7;chr13;PF13_0345;Pf_1055_1055	-2.8278756	Down	1.574522	Up	-1.6575084	Down
Pfa3D7;pfal_chr9;PFI0775w;Pf_545_545	-1.6627856	Down	-2.0027843	Down	-2.7991831	Down
Pfa3D7;pfal_chr9;PFI0775w;Pf_586_586	-1.5756129	Down	-2.018266	Down	-2.8176231	Down
Pfa3D7;chr14;PF14_0010;Pf_124_124	2.2791908	Up	-3.0290282	Down	-1.3757657	Down
Pfa3D7;chr13;PF13_0010;Pf_282_282	2.2003994	Up	3.4736898	Up	7.1998897	Up
Pfa3D7;chr13;PF13_0010;Pf_356_356	1.5696815	Up	3.1178296	Up	5.0508933	Up
Pfa3D7;chr10;PF10_0159;Pf_2271_2271	-1.5115083	Down	2.1394846	Up	1.4378117	Up
Pfa3D7;chr10;PF10_0159;Pf_1850_1850	-1.5137604	Down	2.222334	Up	1.3365281	Up
Pfa3D7;chr13;MAL13P1.348;Pf_2336_2336	6.907977	Up	-2.1124341	Down	3.5582504	Up
Pfa3D7;pfal_chr9;PFI0570w;Pf_3105_3105	-2.9507275	Down	2.146568	Up	-1.361481	Down
Pfa3D7;pfal_chr9;PFI0570w;Pf_3165_3165	-2.7479703	Down	2.093734	Up	-1.3939263	Down
Pfa3D7;chr13;MAL13P1.241;Pf_613_613	1.368465	Up	-2.322775	Down	-1.5698245	Down
Pfa3D7;chr13;MAL13P1.241;Pf_434_434	1.3634138	Up	-2.3343422	Down	-1.603777	Down
Pfa3D7;chr10;PF10_0325;Pf_760_760	-1.6537926	Down	2.3033001	Up	1.3486557	Up
Pfa3D7;chr10;PF10_0325;Pf_804_804	-1.6953782	Down	2.2454681	Up	1.272112	Up
Pfa3D7;chr12;PFL2415w;Pf_661_661	-2.0118923	Down	1.4886668	Up	-1.3387064	Down
Pfa3D7;chr12;PFL2415w;Pf_741_741	-2.0236585	Down	1.3459797	Up	-1.4390975	Down
Pfa3D7;pfal_chr1;PFA0660w;Pf_813_813	1.5588231	Up	-2.1570694	Down	-1.4653244	Down
Pfa3D7;chr10;PF10_0153;Pf_1598_1598	-3.729636	Down	4.2220283	Up	1.1966863	Up
Pfa3D7;chr10;PF10_0153;Pf_1419_1419	-4.158158	Down	4.7660694	Up	1.1594951	Up
Pfa3D7;chr11;PF11_0351;Pf_1440_1440	-2.5509644	Down	2.5206795	Up	-1.0335039	Down
Pfa3D7;chr11;PF11_0351;Pf_1875_1875	-2.4017444	Down	2.2514486	Up	-1.0389842	Down
Pfa3D7;chr14;PF14_0417;Pf_2062_2062	-1.9336282	Down	2.9316459	Up	1.4506017	Up
Pfa3D7;chr11;PF11_0188;Pf_2729_2729	-3.2134414	Down	2.2826262	Up	-1.4638896	Down
Pfa3D7;chr11;PF11_0188;Pf_2302_2302	-3.3492172	Down	2.3005803	Up	-1.3868072	Down
Pfa3D7;chr14;PF14_0417;Pf_2311_2311	-2.1377237	Down	2.9748085	Up	1.3790623	Up
Pfa3D7;chr14;PF14_0528;Pf_641_641	2.273212	Up	-1.3942965	Down	1.613713	Up
Pfa3D7;pfal_chr9;PFI1645c;Pf_1863_1863	-1.6287498	Down	2.433012	Up	1.3314984	Up
Pfa3D7;pfal_chr4;PFD0795w;Pf_3673_3673	-2.0465078	Down	2.2317996	Up	1.1456561	Up
Pfa3D7;pfal_chr4;PFD0795w;Pf_3461_3461	-2.095366	Down	2.2098353	Up	1.1167506	Up
Pfa3D7;chr13;PF13_0293;Pf_1452_1452	2.3117533	Up	-2.2591827	Down	1.0167496	Up
Pfa3D7;pfal_chr3;PFC0730w;Pf_297_297	-2.2577813	Down	1.6334109	Up	-1.3087275	Down
Pfa3D7;pfal_chr3;PFC0730w;Pf_578_578	-2.0575364	Down	1.5362804	Up	-1.2891778	Down
Pfa3D7;chr12;PFL1920c;Pf_191_191	-2.6116123	Down	3.3026404	Up	1.2236903	Up
Pfa3D7;chr12;PFL1920c;Pf_106_106	-2.8986092	Down	3.2770238	Up	1.1357622	Up
Pfa3D7;chr10;PF10_0044;Pf_3402_3402	-1.0047433	Down	-2.2157676	Down	-2.2877798	Down
Pfa3D7;pfal_chr1;PFA0360c;Pf_1103_1103	-1.0568347	Down	-2.1277905	Down	-2.2364416	Down
Pfa3D7;pfal_chr1;PFA0360c;Pf_1023_1023	-1.0271205	Down	-2.2283812	Down	-2.2873046	Down
Pfa3D7;chr10;PF10_0385;Pf_182_182	-1.2000461	Down	2.3001761	Up	1.7549427	Up
Pfa3D7;chr10;PF10_0121;Pf_530_530	-2.4681435	Down	2.9453511	Up	1.2420167	Up
Pfa3D7;chr10;PF10_0121;Pf_323_323	-2.6203308	Down	3.5355518	Up	1.4313328	Up
Pfa3D7;chr12;PFL1140w;Pf_586_586	-2.7420084	Down	3.614216	Up	1.4190435	Up
Pfa3D7;chr12;PFL1140w;Pf_721_721	-2.4284217	Down	3.1782482	Up	1.3824651	Up
Pfa3D7;pfal_chr5;PFE0070w;Pf_5093_5093	3.3386922	Up	-2.3440464	Down	1.268876	Up
Pfa3D7;pfal_chr5;PFE0070w;Pf_4919_4919	2.5233681	Up	-1.8148127	Down	1.2766688	Up
Pfa3D7;pfal_chr5;PFE0070w;Pf_4445_4445	3.1866825	Up	-2.3111258	Down	1.26157	Up
Pfa3D7;chr11;PF11_0044;Pf_4090_4090	-2.170796	Down	2.0218992	Up	-1.1192102	Down
Pfa3D7;chr11;PF11_0044;Pf_4140_4140	-1.9836476	Down	2.0969017	Up	-1.008889	Down
Pfa3D7;chr13;PF13_0242;Pf_1164_1164	-2.0069263	Down	1.8057792	Up	-1.1523931	Down
Pfa3D7;chr12;PFL1210w;Pf_4991_4991	-2.5377922	Down	2.269603	Up	-1.1426278	Down
Pfa3D7;chr13;PF13_0238;Pf_2109_2109	1.6573387	Up	-2.2351403	Down	-1.24716	Down
Pfa3D7;chr12;PFL0545w;Pf_5178_5178	-1.5456474	Down	-1.9655374	Down	-2.789628	Down
Pfa3D7;pfal_chr9;PFI1470c;Pf_5051_5051	-3.174177	Down	3.348782	Up	1.187265	Up
Pfa3D7;pfal_chr9;PFI1470c;Pf_5252_5252	-1.788967	Down	2.0469394	Up	1.1704957	Up

Accession number	[UT8] vs [UT16] Fold-change	Regulation	[UT16] vs [T16] Fold-change	Regulation	[UT8] vs [T16] Fold-change	Regulation
Pfa3D7;chr8;PF08_0011;Pf_4292_4292	-2.9346282	Down	3.3114848	Up	1.1493561	Up
Pfa3D7;chr8;PF08_0011;Pf_4177_4177	-3.2193735	Down	3.6744487	Up	1.0954319	Up
Pfa3D7;pfal_chr2;PFB0915w;Pf_4451_4451	3.1959255	Up	-1.8210655	Down	1.7575203	Up
Pfa3D7;pfal_chr2;PFB0915w;Pf_4311_4311	2.5379899	Up	-1.9809078	Down	1.2894931	Up
Pfa3D7;pfal_chr9;PFI0980w;Pf_1737_1737	-3.1831224	Down	1.9370401	Up	-1.6293901	Down
Pfa3D7;chr6;PFF0290w;Pf_647_647	3.3340068	Up	-1.8933997	Down	1.9423981	Up
Pfa3D7;chr6;PFF0290w;Pf_727_727	3.416312	Up	-2.017248	Down	1.8328837	Up
Pfa3D7;chr6;PFF0580w;Pf_410_410	-2.6080365	Down	1.2507668	Up	-1.9994663	Down
Pfa3D7;pfal_chr4;PFD0285c;Pf_7055_7055	-2.68498	Down	2.0482092	Up	-1.3802407	Down
Pfa3D7;chr14;PF14_0166;Pf_1882_1882	-2.435866	Down	1.6046386	Up	-1.4765134	Down
Pfa3D7;chr14;PF14_0017;Pf_1046_1046	-4.259287	Down	-1.968134	Down	-8.365022	Down
Pfa3D7;pfal_chr9;PFI1775w;Pf_1070_1070	2.1164207	Up	-2.047437	Down	1.036466	Up
Pfa3D7;chr12;PFL2530w;Pf_1235_1235	4.233776	Up	-3.2896192	Down	1.2238855	Up
Pfa3D7;chr12;PFL2530w;Pf_1295_1295	4.9564056	Up	-3.37952	Down	1.4176484	Up
Pfa3D7;chr14;PF14_0439;Pf_1462_1462	-2.0985928	Down	1.9171544	Up	-1.0727118	Down
Pfa3D7;pfal_chr9;PFI1570c;Pf_1587_1587	-2.684471	Down	2.6216226	Up	1.0205669	Up
Pfa3D7;pfal_chr9;PFI1570c;Pf_1527_1527	-2.638795	Down	2.5879521	Up	1.0252516	Up
Pfa3D7;chr12;PFL1420w;Pf_208_208	1.6400124	Up	2.4726036	Up	3.9502313	Up
Pfa3D7;chr14;PF14_0387;Pf_1556_1556	-4.1731877	Down	4.578139	Up	1.1211553	Up
Pfa3D7;chr6;PFF0815w;Pf_1434_1434	-3.2594771	Down	1.5938632	Up	-1.9709706	Down
Pfa3D7;chr6;PFF0815w;Pf_1011_1011	-3.5209892	Down	1.6550932	Up	-1.9033242	Down
Pfa3D7;chr10;PF10_0139;Pf_2463_2463	1.1817696	Up	-2.0557406	Down	-1.5338317	Down
Pfa3D7;pfal_chr5;PFE0040c;Pf_4179_4179	-6.7448196	Down	4.3962507	Up	-1.6123685	Down
Pfa3D7;chr8;PF08_0033;Pf_1372_1372	1.2885947	Up	-2.111792	Down	-1.6996337	Down
Pfa3D7;chr8;PF08_0033;Pf_617_617	1.5590131	Up	-2.1714683	Down	-1.4966362	Down
Pfa3D7;chr11;PF11_0486;Pf_6027_6027	-1.1444573	Down	2.071097	Up	1.664181	Up
Pfa3D7;pfal_chr9;PFI1475w;Pf_5057_5057	1.7368797	Up	1.3359046	Up	2.6031783	Up
Pfa3D7;pfal_chr9;PFI1475w;Pf_4979_4979	1.8507824	Up	1.280495	Up	2.6069367	Up
Pfa3D7;chr6;PFF0995c;Pf_843_843	2.2806127	Up	-1.4993047	Down	1.6156334	Up
Pfa3D7;pfal_chr2;PFB0305c;Pf_330_330	1.7075002	Up	1.6204253	Up	3.6086395	Up
Pfa3D7;chr10;PF10_0346;Pf_926_926	1.6248573	Up	2.0312784	Up	3.8229024	Up
Pfa3D7;chr10;PF10_0346;Pf_1050_1050	1.9658417	Up	1.7594466	Up	4.005187	Up
Pfa3D7;pfal_chr1;PFA0135w;Pf_753_753	4.2746415	Up	-1.8320267	Down	2.4414256	Up
Pfa3D7;pfal_chr9;PFI0700c;Pf_1952_1952	2.0400152	Up	-1.4620714	Down	1.3152833	Up
Pfa3D7;chr11;PF11_0059;Pf_871_871	-2.1742945	Down	1.8904266	Up	-1.1357636	Down
Pfa3D7;chr11;PF11_0059;Pf_1194_1194	-2.0021303	Down	1.9275781	Up	-1.0142312	Down
Pfa3D7;pfal_chr9;PFI0815c;Pf_581_581	2.2953532	Up	-1.740292	Down	1.2480799	Up
Pfa3D7;chr13;PF13_0016;Pf_401_401	-3.011065	Down	2.6941245	Up	-1.0976217	Down
Pfa3D7;chr7;MAL7P1.145;Pf_3662_3662	2.475019	Up	-1.363391	Down	1.7208931	Up
Pfa3D7;chr12;PFL0415w;Pf_308_308	-3.9488878	Down	5.773306	Up	1.5169097	Up
Pfa3D7;chr13;PF13_0359;Pf_1652_1652	-1.8269167	Down	2.0954602	Up	1.1064929	Up
Pfa3D7;chr14;PF14_0328;Pf_386_386	-4.060302	Down	3.713285	Up	-1.029584	Down
Pfa3D7;chr12;PFL2065c;Pf_208_208	-3.46846	Down	2.2591891	Up	-1.4954112	Down
Pfa3D7;chr13;PF13_0358;Pf_108_108	-2.6511471	Down	2.4885461	Up	-1.0667714	Down
Pfa3D7;pfal_chr5;PFE0140c;Pf_159_159	-2.1482377	Down	1.6986786	Up	-1.1678681	Down
Pfa3D7;chr13;PF13_0300;Pf_292_292	-2.5058818	Down	2.6074305	Up	-1.0411448	Down
Pfa3D7;chr12;PFL0500w;Pf_936_936	-2.0532875	Down	2.16308	Up	-1.0267041	Down
Pfa3D7;pfal_chr2;PFB0645c;Pf_521_521	-2.1126616	Down	1.4901347	Up	-1.3179022	Down
Pfa3D7;chr13;MAL13P1.200;Pf_243_243	-1.6714851	Down	2.4677284	Up	1.5179757	Up
Pfa3D7;chr7;MAL7P1.93;Pf_267_267	-2.5428848	Down	1.7360773	Up	-1.3935113	Down
Pfa3D7;pfal_chr2;PFB0465c;Pf_1184_1184	-2.4266772	Down	2.5830522	Up	1.0783855	Up
Pfa3D7;pfal_chr2;PFB0465c;Pf_1301_1301	-2.075149	Down	2.3899643	Up	1.1015898	Up
Pfa3D7;chr13;PF13_0138;Pf_449_449	2.2998688	Up	-1.8185545	Down	1.2755212	Up
Pfa3D7;chr13;PF13_0138;Pf_348_348	2.0076053	Up	-1.8187157	Down	1.1146982	Up
Pfa3D7;chr13;PF13_0193;Pf_792_792	2.3318777	Up	-1.3323479	Down	1.7335961	Up
Pfa3D7;chr13;PF13_0196;Pf_509_509	1.2469194	Up	1.4360006	Up	2.0655704	Up

	[UT8] vs [UT16]		[UT16] vs [T16]		[UT8] vs [T16]	
Accession number	Fold-change	Regulation	Fold-change	Regulation	Fold-change	Regulation
Pfa3D7;chr13;PF13_0191;Pf_1320_1320	-4.3304715	Down	4.248985	Up	-1.0690558	Down
Pfa3D7;chr14;PF14_0455;Pf_2874_2874	-4.0010147	Down	4.3318553	Up	1.0499617	Up
Pfa3D7;chr14;PF14_0455;Pf_2780_2780	-3.170778	Down	4.2589836	Up	1.1904628	Up
Pfa3D7;chr6;PFF0350w;Pf_1343_1343	1.0724431	Up	-2.6524918	Down	-2.43189	Down
Pfa3D7;chr6;PFF0350w;Pf_1275_1275	1.1164124	Up	-2.7484376	Down	-2.5067782	Down
Pfa3D7;chr13;MAL13P1.148;Pf_6290_6290	-3.876722	Down	1.4590592	Up	-2.4872212	Down
Pfa3D7;chr13;MAL13P1.148;Pf_6245_6245	-3.9817088	Down	1.4842865	Up	-2.686139	Down
Pfa3D7;chr13;MAL13P1.148;Pf_6419_6419	-3.8741357	Down	1.4307317	Up	-2.7341955	Down
Pfa3D7;chr12;PFL1435c;Pf_5925_5925	2.0925016	Up	-1.6790187	Down	1.2996938	Up
Pfa3D7;chr13;MAL13P1.255;Pf_1607_1607	-2.0781894	Down	1.7484595	Up	-1.203437	Down
Pfa3D7;chr14;PF14_0164;Pf_1345_1345	4.5073743	Up	-2.5420752	Down	2.0120366	Up
Pfa3D7;chr14;PF14_0164;Pf_1198_1198	4.3859563	Up	-2.6233857	Down	1.8717513	Up
Pfa3D7;chr6;PFF1410c;Pf_1913_1913	2.1038942	Up	-1.4090602	Down	1.4651673	Up
Pfa3D7;chr6;PFF1410c;Pf_1873_1873	2.1223073	Up	-1.3506178	Down	1.510888	Up
Pfa3D7;chr13;PF13_0158;Pf_691_691	2.062277	Up	-1.5659132	Down	1.262949	Up
Pfa3D7;pfal_chr4;PFD0110w;Pf_8553_8553	2.5216913	Up	1.7804286	Up	5.273059	Up
Pfa3D7;chr8;MAL8P1.32;Pf_1368_1368	-2.7198527	Down	3.5369048	Up	1.281342	Up
Pfa3D7;chr8;MAL8P1.32;Pf_1646_1646	-2.7532587	Down	3.2782366	Up	1.1621319	Up
Pfa3D7;chr14;PF14_0662;Pf_1229_1229	-3.9451745	Down	1.8183213	Up	-2.2228746	Down
Pfa3D7;chr12;PFL1585c;Pf_1302_1302	-3.867727	Down	2.514934	Up	-1.4781426	Down
Pfa3D7;chr6;PFF0435w;Pf_1105_1105	-5.806377	Down	3.1709652	Up	-1.6294267	Down
Pfa3D7;chr6;PFF0435w;Pf_848_848	-5.7716966	Down	3.4072568	Up	-1.5683552	Down
Pfa3D7;pfal_chr5;PFE0630c;Pf_767_767	-2.1245313	Down	2.155274	Up	1.0353087	Up
Pfa3D7;pfal_chr5;PFE0630c;Pf_675_675	-2.0229194	Down	2.1912372	Up	1.1434407	Up
Pfa3D7;chr6;PFF1265w;Pf_1028_1028	-2.1133695	Down	1.6467478	Up	-1.2730407	Down
Pfa3D7;chr8;PF08_0031;Pf_855_855	-2.4429276	Down	2.5547714	Up	1.0773838	Up
Pfa3D7;chr8;PF08_0031;Pf_397_397	-2.1958513	Down	2.068691	Up	-1.0184282	Down
Pfa3D7;chr11;PF11_0327;Pf_4453_4453	-4.9204473	Down	3.5190117	Up	-1.2058978	Down
Pfa3D7;chr11;PF11_0327;Pf_4666_4666	-3.7631395	Down	3.0444822	Up	-1.2177485	Down
Pfa3D7;pfal_chr9;PFI0385c;Pf_780_780	-1.688456	Down	-1.4961414	Down	-2.5168774	Down
Pfa3D7;pfal_chr9;PFI0385c;Pf_920_920	-1.2721361	Down	-1.7012541	Down	-2.1301525	Down
Pfa3D7;chr7;MAL7P1.114;Pf_468_468	-2.0042086	Down	2.7159472	Up	1.3940545	Up
Pfa3D7;chr7;MAL7P1.114;Pf_548_548	-1.8231763	Down	2.6554916	Up	1.3906224	Up
Pfa3D7;pfal_chr5;PFE0060w;Pf_518_518	-5.3281054	Down	2.0561848	Up	-2.4857922	Down
Pfa3D7;pfal_chr5;PFE0060w;Pf_814_814	-5.7557535	Down	2.0252545	Up	-2.4844356	Down
Pfa3D7;pfal_chr3;PFC0950c;Pf_3040_3040	-2.232961	Down	1.821259	Up	-1.2085155	Down
Pfa3D7;pfal_chr3;PFC0950c;Pf_3143_3143	-2.2954097	Down	1.7504041	Up	-1.3027165	Down
Pfa3D7;pfal_chr9;PFI0380c;Pf_201_201	-2.6261756	Down	2.1799781	Up	-1.1045243	Down
Pfa3D7;pfal_chr9;PFI0380c;Pf_665_665	-2.458711	Down	2.2906163	Up	-1.0638692	Down
Pfa3D7;pfal_chr4;PFD0430c;Pf_2308_2308	-1.095171	Down	-3.1989157	Down	-3.2974033	Down
Pfa3D7;pfal_chr4;PFD0430c;Pf_2231_2231	-1.0225754	Down	-3.6031296	Down	-3.7321703	Down
Pfa3D7;chr8;PF08_0050;Pf_1697_1697	-1.2199521	Down	-1.9708215	Down	-2.3357413	Down
Pfa3D7;chr8;PF08_0052;Pf_1970_1970	1.7805324	Up	-2.1994262	Down	-1.2757422	Down
Pfa3D7;chr8;PF08_0052;Pf_1847_1847	1.5691911	Up	-2.1891856	Down	-1.4546167	Down
Pfa3D7;chr10;PF10_0025;Pf_1773_1773	4.8344526	Up	-1.480542	Down	3.1795444	Up
Pfa3D7;chr10;PF10_0025;Pf_1693_1693	4.645553	Up	-1.4767908	Down	3.0494657	Up
Pfa3D7;pfal_chr3;PFC1080c;Pf_611_611	-1.6866461	Down	2.5910015	Up	1.5483301	Up
Pfa3D7;pfal_chr1;PFA0680c;Pf_415_415	-12.172911	Down	16.184881	Up	1.771123	Up
Pfa3D7;pfal_chr2;PFB0985c;Pf_496_496	-14.235644	Down	17.96075	Up	1.4098294	Up
Pfa3D7;chr7;MAL7P1.5;Pf_536_536	-14.227176	Down	15.705601	Up	1.447013	Up
Pfa3D7;pfal_chr2;PFB0985c;Pf_536_536	-14.156301	Down	17.587616	Up	1.596347	Up
Pfa3D7;chr11;PF11_0025;Pf_516_516	-14.747547	Down	20.582361	Up	1.6910819	Up
Pfa3D7;chr7;MAL7P1.58;Pf_607_607	-1.9902558	Down	3.6861	Up	2.139585	Up
Pfa3D7;pfal_chr3;PFC1080c;Pf_531_531	-14.621341	Down	18.965921	Up	1.2556581	Up
Pfa3D7;chr6;PFF0060w;Pf_531_531	-12.844174	Down	10.033199	Up	-1.1665459	Down
Pfa3D7;chr7;MAL7P1.5;Pf_225_225	-14.369769	Down	15.330968	Up	1.2063392	Up

	[UT8] vs [UT16]		[UT16] vs [T16]		[UT8] vs [T16]	
**Accession number**	**Fold-change**	**Regulation**	**Fold-change**	**Regulation**	**Fold-change**	**Regulation**
Pfa3D7;chr10;PF10_0390;Pf_387_387	-11.082863	Down	16.343023	Up	1.5041364	Up
Pfa3D7;chr10;PF10_0390;Pf_307_307	-4.445926	Down	5.1851993	Up	1.2318038	Up
Pfa3D7;pfal_chr1;PFA0680c;Pf_375_375	-11.742825	Down	16.41868	Up	1.4356511	Up
Pfa3D7;pfal_chr1;PFA0065w;Pf_378_378	-16.741892	Down	16.271128	Up	1.1283872	Up
Pfa3D7;chr6;PFF1525c;Pf_519_519	-13.75505	Down	23.788952	Up	1.9603627	Up
Pfa3D7;chr6;PFF0060w;Pf_491_491	-15.346157	Down	11.841101	Up	-1.193001	Down
Pfa3D7;chr14;PF14_0036;Pf_768_768	-2.2164028	Down	2.1351469	Up	1.0214821	Up
Pfa3D7;chr13;PF13_0234;Pf_1406_1406	-3.198376	Down	2.7947705	Up	-1.1232942	Down
Pfa3D7;chr13;PF13_0234;Pf_1573_1573	-3.496845	Down	2.8058496	Up	-1.2512175	Down
Pfa3D7;chr11;PF11_0208;Pf_275_275	-1.2232155	Down	2.0564742	Up	1.6733954	Up
Pfa3D7;chr11;PF11_0208;Pf_429_429	-1.2767444	Down	2.179852	Up	1.6852239	Up
Pfa3D7;chr14;PF14_0280;Pf_527_527	-2.100241	Down	3.4192016	Up	1.5607582	Up
Pfa3D7;chr14;PF14_0076;Pf_993_993	2.642956	Up	-2.1001332	Down	1.2083521	Up
Pfa3D7;chr14;PF14_0076;Pf_1121_1121	2.5060782	Up	-1.8126519	Down	1.3491427	Up
Pfa3D7;pfal_chr1;PFA0635c;Pf_1085_1085	3.119798	Up	-1.6738799	Down	1.7950197	Up
Pfa3D7;pfal_chr1;PFA0635c;Pf_1045_1045	3.0200553	Up	-1.6550243	Down	1.7150286	Up
Pfa3D7;pfal_chr1;PFA0700c;Pf_146_146	-19.528961	Down	6.667987	Up	-2.8395112	Down
Pfa3D7;pfal_chr2;PFB0110w;Pf_595_595	-3.0793016	Down	5.4588065	Up	1.7382826	Up
Pfa3D7;pfal_chr9;PFI1750c;Pf_493_493	-7.437633	Down	3.9933305	Up	-1.7457918	Down
Pfa3D7;chr11;PF11_0504;Pf_813_813	6.4077682	Up	-2.5528185	Down	2.29016	Up
Pfa3D7;pfal_chr1;PFA0610c;Pf_467_467	3.1165645	Up	-1.4155864	Down	2.1328273	Up
Pfa3D7;pfal_chr2;PFB0110w;Pf_506_506	-3.0007675	Down	5.597504	Up	1.8202013	Up
Pfa3D7;chr11;PF11_0041;Pf_433_433	10.484813	Up	-1.7749676	Down	5.774433	Up
Pfa3D7;pfal_chr9;PFI1750c;Pf_448_448	-6.5403886	Down	4.294951	Up	-1.4006205	Down
Pfa3D7;chr7;MAL7P1.6;Pf_606_606	4.831429	Up	-2.059099	Down	2.3167846	Up
Pfa3D7;chr10;PF10_0013;Pf_442_442	-12.01702	Down	1.9517655	Up	-5.3965783	Down
Pfa3D7;chr7;MAL7P1.6;Pf_465_465	5.409175	Up	-1.9600607	Down	2.6152167	Up
Pfa3D7;chr14;PF14_0753;Pf_593_593	4.5129123	Up	-2.049653	Down	2.0845304	Up
Pfa3D7;chr14;PF14_0753;Pf_673_673	5.0494676	Up	-2.0336802	Down	2.3384826	Up
Pfa3D7;chr14;PF14_0743;Pf_516_516	-18.477362	Down	6.870784	Up	-2.1226041	Down
Pfa3D7;pfal_chr4;PFD1205w;Pf_524_524	2.441628	Up	-2.2008326	Down	1.0806454	Up
Pfa3D7;chr14;PF14_0743;Pf_435_435	-16.033415	Down	6.78397	Up	-1.7768573	Down
Pfa3D7;pfal_chr2;PFB0953w;Pf_418_418	-5.8325295	Down	3.4809098	Up	-1.7035755	Down
Pfa3D7;pfal_chr2;PFB0953w;Pf_458_458	-5.770143	Down	3.3962226	Up	-1.6120422	Down
Pfa3D7;pfal_chr4;PFD1205w;Pf_307_307	3.2355912	Up	-2.4126735	Down	1.3059194	Up
Pfa3D7;chr10;PF10_0024;Pf_1245_1245	9.731666	Up	-2.1307514	Down	4.7681956	Up
Pfa3D7;pfal_chr9;PFI0085c;Pf_466_466	-4.3040543	Down	6.7959857	Up	1.8294202	Up
Pfa3D7;pfal_chr4;PFD1200c;Pf_504_504	2.8947558	Up	-1.5951806	Down	1.900066	Up
Pfa3D7;chr14;PF14_0742;Pf_408_408	-8.379513	Down	5.786194	Up	-1.577944	Down
Pfa3D7;chr14;PF14_0742;Pf_451_451	-13.151387	Down	7.759161	Up	-1.2627734	Down
Pfa3D7;chr8;MAL8P1.161;Pf_563_563	-5.7168074	Down	2.8263109	Up	-1.7906607	Down
Pfa3D7;chr8;MAL8P1.160;Pf_462_462	9.714537	Up	-2.5875812	Down	3.696914	Up
Pfa3D7;chr8;MAL8P1.161;Pf_503_503	-4.970775	Down	2.770459	Up	-1.6824257	Down
Pfa3D7;chr8;MAL8P1.160;Pf_80_80	10.756849	Up	-2.6552267	Down	4.2037883	Up
Pfa3D7;pfal_chr1;PFA0670c;Pf_448_448	3.5089397	Up	-1.6207849	Down	2.2298	Up
Pfa3D7;pfal_chr1;PFA0670c;Pf_488_488	3.8055284	Up	-1.9384575	Down	1.9721947	Up
Pfa3D7;pfal_chr2;PFB0932w;Pf_653_653	2.8328364	Up	-2.057993	Down	1.4239669	Up
Pfa3D7;pfal_chr2;PFB0932w;Pf_693_693	3.2944913	Up	-1.9529811	Down	1.65885	Up
Pfa3D7;chr8;MAL8P1.163;Pf_668_668	-1.3230604	Down	-1.8344754	Down	-2.278646	Down
Pfa3D7;pfal_chr4;PFD0090c;Pf_644_644	-9.765196	Down	4.604674	Up	-2.0381238	Down
Pfa3D7;chr13;MAL13P1.470;Pf_948_948	-1.1379123	Down	-2.2227345	Down	-2.7602162	Down
Pfa3D7;pfal_chr4;PFD0090c;Pf_684_684	-8.072611	Down	4.7576704	Up	-1.5444572	Down
Pfa3D7;chr12;PFL2565w;Pf_340_340	2.4052715	Up	-1.3024622	Down	1.8671572	Up
Pfa3D7;chr12;PFL2565w;Pf_292_292	2.538234	Up	-1.2785465	Down	1.999503	Up
Pfa3D7;pfal_chr4;PFD1185w;Pf_617_617	2.1813838	Up	-1.2404678	Down	1.6891365	Up

	[UT8] vs [UT16]		[UT16] vs [T16]		[UT8] vs [T16]	
Accession number	Fold-change	Regulation	Fold-change	Regulation	Fold-change	Regulation
Pfa3D7;chr6;PFF0085w;Pf_626_626	-2.7358804	Down	1.7423583	Up	-1.6947526	Down
Pfa3D7;chr13;MAL13P1.58;Pf_306_306	-1.3610357	Down	-2.0420663	Down	-2.7970781	Down
Pfa3D7;pfal_chr4;PFD0080c;Pf_1522_1522	-4.8862348	Down	3.215397	Up	-1.5407302	Down
Pfa3D7;pfal_chr2;PFB0080c;Pf_1299_1299	-2.1814597	Down	1.4946382	Up	-1.460648	Down
Pfa3D7;pfal_chr5;PFE1600w;Pf_1261_1261	-2.168702	Down	1.6859891	Up	-1.2720453	Down
Pfa3D7;pfal_chr9;PFI1785w;Pf_846_846	-4.29208	Down	2.7016685	Up	-1.7864549	Down
Pfa3D7;chr14;PF14_0731;Pf_855_855	-7.0361114	Down	2.6133022	Up	-2.5398655	Down
Pfa3D7;chr6;PFF0075c;Pf_518_518	-1.7773234	Down	2.1330051	Up	1.0917058	Up
Pfa3D7;pfal_chr2;PFB0080c;Pf_828_828	-2.2935495	Down	1.6367139	Up	-1.4476416	Down
Pfa3D7;pfal_chr5;PFE1605w;Pf_964_964	-5.3554654	Down	2.481924	Up	-2.0800295	Down
Pfa3D7;chr12;PFL0050c;Pf_1776_1776	-12.3981	Down	11.368954	Up	-1.005499	Down
Pfa3D7;chr7;MAL7P1.7;Pf_1685_1685	5.5064926	Up	-2.4892573	Down	2.1504314	Up
Pfa3D7;pfal_chr9;PFI0130c;Pf_971_971	-3.363166	Down	3.7384942	Up	1.1448344	Up
Pfa3D7;chr7;MAL7P1.7;Pf_1483_1483	6.1524944	Up	-2.6940439	Down	2.2373412	Up
Pfa3D7;pfal_chr5;PFE1605w;Pf_1103_1103	-5.4951067	Down	2.4667523	Up	-2.251261	Down
Pfa3D7;pfal_chr9;PFI0130c;Pf_916_916	-1.468465	Down	2.0313892	Up	1.3944753	Up
Pfa3D7;pfal_chr4;PFD0095c;Pf_1608_1608	2.0294538	Up	-1.3281013	Down	1.4322768	Up
Pfa3D7;chr14;PF14_0731;Pf_774_774	-7.4003673	Down	2.5689197	Up	-2.669752	Down
Pfa3D7;pfal_chr4;PFD0080c;Pf_1422_1422	-5.63188	Down	3.123916	Up	-1.7883849	Down
Pfa3D7;chr14;PF14_0746;Pf_1295_1295	-2.8210497	Down	1.4256351	Up	-2.0661604	Down
Pfa3D7;pfal_chr4;PFD1170c;Pf_597_597	2.057674	Up	-1.3426508	Down	1.6134546	Up
Pfa3D7;chr6;PFF1510w;Pf_554_554	-1.7261778	Down	2.3750174	Up	1.2346528	Up
Pfa3D7;chr12;PFL0050c;Pf_1876_1876	-12.921554	Down	10.256395	Up	-1.1335324	Down
Pfa3D7;pfal_chr2;PFB0105c;Pf_653_653	6.385202	Up	-2.7109177	Down	2.3682828	Up
Pfa3D7;chr8;MAL8P1.4;Pf_1281_1281	3.0231526	Up	-1.7348796	Down	1.6919069	Up
Pfa3D7;chr8;MAL8P1.4;Pf_1160_1160	3.5550175	Up	-1.7903395	Down	1.7643588	Up
Pfa3D7;pfal_chr4;PFD1140w;Pf_678_678	-14.361793	Down	9.397167	Up	-1.3178575	Down
Pfa3D7;chr10;PF10_0163;Pf_823_823	7.158105	Up	-1.9911414	Down	2.8014169	Up
Pfa3D7;chr7;MAL7P1.172;Pf_2606_2606	2.2989807	Up	-1.7884779	Down	1.2829	Up
Pfa3D7;chr10;PF10_0021;Pf_625_625	2.3914838	Up	-2.2293043	Down	1.0616517	Up
Pfa3D7;chr7;MAL7P1.172;Pf_2504_2504	2.217823	Up	-1.9119121	Down	1.1601238	Up
Pfa3D7;chr10;PF10_0163;Pf_723_723	5.0231357	Up	-2.0496848	Down	2.3391232	Up
Pfa3D7;pfal_chr4;PFD1140w;Pf_816_816	-13.87778	Down	7.7067475	Up	-1.6392642	Down
Pfa3D7;pfal_chr2;PFB0105c;Pf_778_778	5.9514804	Up	-2.5926416	Down	2.3320415	Up
Pfa3D7;pfal_chr2;PFB0900c;Pf_674_674	8.32104	Up	-2.353114	Down	3.110402	Up
Pfa3D7;chr10;PF10_0022;Pf_301_301	5.384609	Up	-2.7640636	Down	2.029007	Up
Pfa3D7;chr10;PF10_0162;Pf_2326_2326	2.1793416	Up	-1.429345	Down	1.4532726	Up
Pfa3D7;chr8;PF08_0001;Pf_399_399	-10.29007	Down	1.8342011	Up	-5.189215	Down
Pfa3D7;chr7;PF07_0107;Pf_2324_2324	3.9584477	Up	-1.7132331	Down	2.2206244	Up
Pfa3D7;chr11;PF11_0511;Pf_500_500	-11.523944	Down	5.4561467	Up	-1.7327704	Down
Pfa3D7;pfal_chr3;PFC0090w;Pf_708_708	7.5489163	Up	-1.9580027	Down	3.8958936	Up
Pfa3D7;pfal_chr2;PFB0910w;Pf_597_597	2.4714823	Up	-1.7718282	Down	1.3098428	Up
Pfa3D7;chr10;PF10_0375;Pf_1060_1060	-2.4583144	Down	1.9690932	Up	-1.1554798	Down
Pfa3D7;chr13;MAL13P1.465;Pf_284_284	-5.731646	Down	5.480512	Up	-1.109035	Down
Pfa3D7;pfal_chr5;PFE1615c;Pf_377_377	-13.223868	Down	9.428351	Up	-1.1663752	Down
Pfa3D7;chr11;PF11_0508;Pf_768_768	-2.7459157	Down	2.4803853	Up	-1.033287	Down
Pfa3D7;pfal_chr1;PFA0630c;Pf_1339_1339	-4.582593	Down	3.749702	Up	-1.2042104	Down
Pfa3D7;chr7;PF07_0004;Pf_2236_2236	3.838431	Up	-2.4564934	Down	1.6005038	Up
Pfa3D7;pfal_chr2;PFB0106c;Pf_663_663	3.8329258	Up	-2.1253107	Down	1.7700922	Up
Pfa3D7;chr12;PFL2545c;Pf_21_21	8.192614	Up	-1.9778045	Down	4.171328	Up
Pfa3D7;chr10;PF10_0376;Pf_1454_1454	-3.0622528	Down	2.9513013	Up	-1.1566837	Down
Pfa3D7;chr11;PF11_0508;Pf_1075_1075	-2.8213005	Down	2.7966344	Up	1.1513498	Up
Pfa3D7;chr14;PF14_0744;Pf_449_449	-3.3754396	Down	2.8985603	Up	-1.1447259	Down
Pfa3D7;pfal_chr2;PFB0106c;Pf_725_725	3.9601994	Up	-1.9562894	Down	1.9563502	Up
Pfa3D7;pfal_chr9;PFI1795c;Pf_681_681	1.0734723	Up	2.619901	Up	2.866913	Up
Pfa3D7;pfal_chr1;PFA0630c;Pf_1458_1458	-4.557683	Down	3.5666807	Up	-1.3110712	Down

	[UT8] vs [UT16]		[UT16] vs [T16]		[UT8] vs [T16]	
**Accession number**	**Fold-change**	**Regulation**	**Fold-change**	**Regulation**	**Fold-change**	**Regulation**
Pfa3D7;chr14;PF14_0736;Pf_2555_2555	-1.3149757	Down	2.282948	Up	1.5854093	Up
Pfa3D7;pfal_chr3;PFC0090w;Pf_668_668	8.42267	Up	-1.9247242	Down	4.2875767	Up
Pfa3D7;chr8;PF08_0001;Pf_479_479	-9.737394	Down	1.8253688	Up	-4.7764935	Down
Pfa3D7;chr14;PF14_0014;Pf_734_734	-8.508517	Down	12.137223	Up	1.3940241	Up
Pfa3D7;pfal_chr5;PFE0050w;Pf_635_635	-2.267577	Down	1.8383789	Up	-1.2518946	Down
Pfa3D7;chr14;PF14_0014;Pf_673_673	-4.109481	Down	9.5568495	Up	2.0378098	Up
Pfa3D7;pfal_chr2;PFB0910w;Pf_557_557	2.5065773	Up	-1.7768822	Down	1.3078961	Up
Pfa3D7;chr7;PF07_0004;Pf_2826_2826	5.211747	Up	-2.760482	Down	1.9026057	Up
Pfa3D7;chr7;MAL7P1.173;Pf_848_848	5.697322	Up	-2.6090024	Down	1.8825495	Up
Pfa3D7;chr14;PF14_0744;Pf_321_321	-3.1495187	Down	2.7769747	Up	-1.3117546	Down
Pfa3D7;chr12;PFL0070c;Pf_3270_3270	1.7136033	Up	-2.9978857	Down	-1.7079077	Down
Pfa3D7;chr13;MAL13P1.62;Pf_115_115	1.0281209	Up	-2.1107218	Down	-1.9101074	Down
Pfa3D7;pfal_chr2;PFB0921c;Pf_291_291	-2.2390137	Down	2.1289802	Up	-1.020977	Down
Pfa3D7;pfal_chr2;PFB0921c;Pf_427_427	-2.0168843	Down	1.5127556	Up	-1.2527575	Down
Pfa3D7;pfal_chr5;PFE0050w;Pf_715_715	-2.1565275	Down	1.9589945	Up	-1.1480938	Down
Pfa3D7;chr7;PF07_0107;Pf_1932_1932	4.037921	Up	-1.5748223	Down	2.4966946	Up
Pfa3D7;chr11;PF11_0014;Pf_532_532	-11.779254	Down	23.82255	Up	2.028637	Up
Pfa3D7;chr11;PF11_0014;Pf_452_452	-14.781583	Down	24.622532	Up	1.6763619	
Pfa3D7;chr14;PF14_0167;Pf_351_351	-2.4564393	Down	2.1897945	Up	-1.0611019	Down
Pfa3D7;pfal_chr4;PFD1135c;Pf_341_341	2.3691008	Up	-1.7354866	Down	1.427211	Up
Pfa3D7;chr14;PF14_0708;Pf_6246_6246	1.7390051	Up	-3.736057	Down	-2.3759363	Down
Pfa3D7;chr13;PF13_0194;Pf_528_528	3.332227	Up	-1.955954	Down	1.6094962	Up
Pfa3D7;chr14;PF14_0263;Pf_5426_5426	1.045192	Up	-2.1869154	Down	-2.0339787	Down
Pfa3D7;chr11;PF11_0505;Pf_56_56	2.1738598	Up	-1.3860486	Down	1.5965413	Up
Pfa3D7;pfal_chr9;PFI0090c;Pf_977_977	2.7168112	Up	-1.6681049	Down	1.5012215	Up
Pfa3D7;chr13;PF13_0194;Pf_481_481	3.8782394	Up	-1.832638	Down	2.0153966	Up
Pfa3D7;chr10;PF10_0350;Pf_1705_1705	-8.536722	Down	4.369498	Up	-1.6627616	Down
Pfa3D7;chr10;PF10_0350;Pf_948_948	-6.4853215	Down	4.048899	Up	-1.4363441	Down
SSU_236-1662_1228_1228	2.7973826	Up	-2.2819302	Down	1.349374	Up
Pfa3D7;chr12;PFL1965w;Pf_616_616	-2.8421714	Down	2.8767483	Up	1.0126941	Up
LSU_2335-5116_2377_2377	2.5093346	Up	-2.5093255	Down	1.0042742	Up
Pfa3D7;chr10;PF10_0144;Pf_745_745	-4.1488457	Down	3.4995437	Up	-1.0707809	Down
Pfa3D7;chr8;PF08_0006;Pf_306_306	-3.4181998	Down	2.8114352	Up	-1.1249486	Down
Pfa3D7;chr10;PF10_0144;Pf_644_644	-4.0015273	Down	3.50876	Up	1.010981	Up
Pfa3D7;chr8;PF08_0006;Pf_627_627	-3.235712	Down	2.6261597	Up	-1.1658174	Down
Pfa3D7;pfal_chr3;PFC0745c;Pf_687_687	-1.8020915	Down	2.3864014	Up	1.3596641	Up
Pfa3D7;chr13;MAL13P1.190;Pf_1444_1444	-2.143223	Down	1.6686764	Up	-1.3007025	Down
Pfa3D7;chr13;MAL13P1.190;Pf_1201_1201	-2.0468576	Down	1.5931171	Up	-1.2713996	Down
Pfa3D7;chr6;PFF0420c;Pf_375_375	-1.5837079	Down	2.074155	Up	1.2860857	Up
Pfa3D7;pfal_chr5;PFE0915c;Pf_512_512	-1.6273109	Down	2.036897	Up	1.2653822	Up
Pfa3D7;chr11;PF11_0286;Pf_1172_1172	1.4734807	Up	-2.1611001	Down	-1.689687	Down
Pfa3D7;chr8;PF08_0044;Pf_2173_2173	2.0579975	Up	-1.6217034	Down	1.2382164	Up
Pfa3D7;chr8;PF08_0044;Pf_2233_2233	2.1079915	Up	-1.5765045	Down	1.279243	Up
Pfa3D7;chr13;MAL13P1.185;Pf_833_833	-4.126595	Down	3.3529632	Up	-1.2056074	Down
Pfa3D7;chr13;MAL13P1.185;Pf_503_503	-4.471524	Down	4.0361457	Up	1.0026437	Up
Pfa3D7;chr14;PF14_0264;Pf_6759_6759	4.5641456	Up	-3.2123468	Down	1.371323	Up
Pfa3D7;chr14;PF14_0264;Pf_6819_6819	5.471887	Up	-3.162354	Down	1.6185819	Up
Pfa3D7;pfal_chr5;PFE0795c;Pf_734_734	-2.3001156	Down	2.1104722	Up	-1.0429752	Down
Pfa3D7;chr10;PF10_0093;Pf_1272_1272	-2.2298915	Down	2.18862	Up	-1.0488069	Down
Pfa3D7;chr7;PF07_0110;Pf_1370_1370	-2.2044966	Down	1.7065662	Up	-1.376114	Down
Pfa3D7;pfal_chr5;PFE0795c;Pf_878_878	-2.304825	Down	1.9461181	Up	-1.0754445	Down
Pfa3D7;chr10;PF10_0124;Pf_4182_4182	2.419353	Up	-1.4833008	Down	1.5377187	Up
Pfa3D7;chr10;PF10_0093;Pf_1141_1141	-2.492689	Down	2.169578	Up	-1.1772945	Down
Pfa3D7;pfal_chr5;PFE0660c;Pf_667_667	1.1181313	Up	2.6342566	Up	3.0269396	Up
Pfa3D7;pfal_chr5;PFE0660c;Pf_540_540	1.090057	Up	2.5088723	Up	2.813126	Up

	[UT8] vs [UT16]		[UT16] vs [T16]		[UT8] vs [T16]	
Accession number	Fold-change	Regulation	Fold-change	Regulation	Fold-change	Regulation
Pfa3D7;chr13;MAL13P1.284;Pf_723_723	-3.419544	Down	3.174273	Up	-1.0893168	Down
Pfa3D7;chr13;MAL13P1.284;Pf_494_494	-3.032876	Down	2.9307797	Up	1.032873	Up
Pfa3D7;chr10;PF10_0363;Pf_1391_1391	-3.1516364	Down	3.2621412	Up	-1.0882354	Down
Pfa3D7;chr10;PF10_0363;Pf_1574_1574	-3.6408064	Down	3.3990989	Up	-1.169273	Down
Pfa3D7;pfal_chr2;PFB0500c;Pf_508_508	-1.8681011	Down	2.0175374	Up	1.0947587	Up
Pfa3D7;pfal_chr9;PFI0155c;Pf_401_401	-2.9998982	Down	3.620843	Up	1.1583738	Up
Pfa3D7;chr12;PFL0955c;Pf_145_145	1.1559472	Up	-2.3955426	Down	-1.9336014	Down
Pfa3D7;chr12;PFL0955c;Pf_225_225	1.2143227	Up	-2.2900424	Down	-1.9030513	Down
Pfa3D7;pfal_chr5;PFE1295c;Pf_2359_2359	-3.4225738	Down	2.3683147	Up	-1.5628374	Down
Pfa3D7;pfal_chr5;PFE0800w;Pf_3084_3084	-2.6031313	Down	2.3892555	Up	-1.1313316	Down
Pfa3D7;pfal_chr5;PFE0800w;Pf_3124_3124	-2.8399572	Down	2.4013197	Up	-1.1277173	Down
Pfa3D7;pfal_chr5;PFE0905w;Pf_4004_4004	-2.9518356	Down	2.7964365	Up	-1.1018062	Down
Pfa3D7;pfal_chr5;PFE1295c;Pf_2279_2279	-2.5773022	Down	2.3438842	Up	-1.0459466	Down
Pfa3D7;chr7;MAL7P1.23;Pf_3414_3414	-1.5665973	Down	2.0912817	Up	1.2901577	Up
Pfa3D7;pfal_chr5;PFE0905w;Pf_3938_3938	-2.9139564	Down	2.7509663	Up	-1.0884496	Down
Pfa3D7;chr7;MAL7P1.23;Pf_3474_3474	-2.2758439	Down	2.3989806	Up	1.0520893	Up
Pfa3D7;chr10;PF10_0057;Pf_4568_4568	-2.4223557	Down	2.4572306	Up	1.0078048	Up
Pfa3D7;chr10;PF10_0057;Pf_4448_4448	-2.05557	Down	1.8898593	Up	-1.1441993	Down
Pfa3D7;pfal_chr1;PFA0545c;Pf_3402_3402	-2.0527635	Down	2.0507321	Up	1.1504886	Up
Pfa3D7;pfal_chr4;PFD0475c;Pf_3371_3371	-2.6275418	Down	2.3650987	Up	-1.1742272	Down
Pfa3D7;chr10;PF10_0378;Pf_2499_2499	2.2419875	Up	-1.4539278	Down	1.4174424	Up
Pfa3D7;chr13;PF13_0198;Pf_9304_9304	1.9959669	Up	1.4904946	Up	3.451243	Up
Pfa3D7;chr13;PF13_0198;Pf_9162_9162	2.1702416	Up	1.4848945	Up	3.6009023	Up
Pfa3D7;chr13;MAL13P1.176;Pf_8782_8782	2.1452272	Up	1.6220624	Up	3.9825583	Up
Pfa3D7;chr13;MAL13P1.176;Pf_9179_9179	2.0013604	Up	1.7119403	Up	3.9941835	Up
Pfa3D7;chr13;MAL13P1.176;Pf_8877_8877	2.0948572	Up	1.7362312	Up	4.099695	Up
Pfa3D7;chr13;PF13_0241;Pf_1495_1495	-2.254179	Down	2.2022681	Up	-1.0579233	Down
Pfa3D7;chr10;PF10_0402;Pf_719_719	-3.1513875	Down	4.3122673	Up	1.4934231	Up
Pfa3D7;chr14;PF14_0772;Pf_552_552	-2.6490343	Down	3.8646092	Up	1.4528358	Up
Pfa3D7;chr6;PFF1555w;Pf_890_890	-4.3974285	Down	5.3483086	Up	1.2215213	Up
Pfa3D7;chr6;PFF1555w;Pf_930_930	-6.43931	Down	7.4498806	Up	1.0204152	Up
Pfa3D7;chr10;PF10_0403;Pf_848_848	1.1285112	Up	3.26981	Up	3.7442696	Up
Pfa3D7;chr13;MAL13P1.515;Pf_851_851	-6.3150325	Down	7.445009	Up	1.0166062	Up
Pfa3D7;chr10;PF10_0006;Pf_810_810	-4.621216	Down	5.004864	Up	1.0744042	Up
Pfa3D7;chr14;PF14_0006;Pf_469_469	-5.669228	Down	6.2233095	Up	1.2369928	Up
Pfa3D7;chr11;PF11_0529;Pf_700_700	-3.6328063	Down	3.6260712	Up	1.1270868	Up
Pfa3D7;pfal_chr2;PFB1035w;Pf_754_754	-13.558687	Down	25.357733	Up	1.9446347	Up
Pfa3D7;chr11;PF11_0021;Pf_632_632	-9.055094	Down	16.27476	Up	1.9820544	Up
Pfa3D7;chr8;PF08_0104;Pf_532_532	-7.784257	Down	5.7991905	Up	-1.3569458	Down
Pfa3D7;pfal_chr4;PFD0060w;Pf_761_761	-5.7659855	Down	4.481329	Up	-1.1199347	Down
Pfa3D7;chr10;PF10_0398;Pf_680_680	-7.38251	Down	10.753578	Up	1.6821753	Up
Pfa3D7;pfal_chr1;PFA0760w;Pf_704_704	-3.758719	Down	4.1440854	Up	1.1292542	Up
Pfa3D7;pfal_chr2;PFB1050w;Pf_722_722	-1.9888104	Down	2.2239938	Up	1.1122444	Up
Pfa3D7;pfal_chr9;PFI0075w;Pf_926_926	-4.739805	Down	7.3953633	Up	1.4682221	Up
Pfa3D7;chr12;PFL2645c;Pf_539_539	-4.089321	Down	4.7961297	Up	1.2292308	Up
Pfa3D7;chr13;MAL13P1.495;Pf_629_629	-7.960636	Down	6.330164	Up	-1.125597	Down
Pfa3D7;chr12;PFL2645c;Pf_737_737	-4.385279	Down	6.3489823	Up	1.3220865	Up
Pfa3D7;pfal_chr3;PFC0010c;Pf_529_529	-4.1370587	Down	4.291068	Up	1.1068542	Up
Pfa3D7;chr7;PF07_0132;Pf_824_824	-8.002911	Down	14.885843	Up	2.068332	Up
Pfa3D7;chr14;PF14_0769;Pf_801_801	-3.212447	Down	6.0739875	Up	1.9131521	Up
Pfa3D7;pfal_chr9;PFI0070w;Pf_732_732	-4.156341	Down	5.308419	Up	1.4617572	Up
Pfa3D7;pfal_chr4;PFD0040c;Pf_682_682	-4.683546	Down	7.4308267	Up	1.5191332	Up
Pfa3D7;pfal_chr1;PFA0020w;Pf_886_886	-4.3049874	Down	4.80571	Up	1.1426879	Up
Pfa3D7;chr10;PF10_0006;Pf_460_460	-5.00775	Down	5.0125933	Up	1.0818236	Up
Pfa3D7;chr11;PF11_0010;Pf_526_526	-4.7730026	Down	8.302673	Up	1.7659532	Up
Pfa3D7;pfal_chr2;PFB1035w;Pf_435_435	-5.6803527	Down	6.2299547	Up	1.1564608	Up

	[UT8] vs [UT16]		[UT16] vs [T16]		[UT8] vs [T16]	
Accession number	Fold-change	Regulation	Fold-change	Regulation	Fold-change	Regulation
Pfa3D7;chr14;PF14_0006;Pf_679_679	-8.041793	Down	8.240611	Up	-1.1545343	Down
Pfa3D7;chr13;MAL13P1.515;Pf_782_782	-3.8305485	Down	6.302259	Up	1.3637283	Up
Pfa3D7;pfal_chr4;PFD0040c;Pf_762_762	-2.538831	Down	7.2192564	Up	2.3873074	Up
Pfa3D7;chr14;PF14_0002;Pf_762_762	-4.155622	Down	4.6709647	Up	1.0910562	Up
Pfa3D7;chr6;PFF1575w;Pf_934_934	-4.3867745	Down	4.10894	Up	-1.1858706	Down
Pfa3D7;chr11;PF11_0529;Pf_531_531	-4.6434484	Down	4.222048	Up	1.0508763	Up
Pfa3D7;pfal_chr4;PFD0055w;Pf_883_883	-14.1592455	Down	15.565309	Up	1.3161201	Up
Pfa3D7;chr12;PFL2615w;Pf_801_801	-5.1068406	Down	6.8752136	Up	1.6030923	Up
Pfa3D7;chr12;PFL2585c;Pf_882_882	-3.5084867	Down	4.1147	Up	1.3246471	Up
Pfa3D7;chr14;PF14_0002;Pf_657_657	-4.563509	Down	5.3797154	Up	1.0431743	Up
Pfa3D7;chr6;PFF0035c;Pf_678_678	-3.7739615	Down	3.3317773	Up	-1.2685941	Down
Pfa3D7;chr14;PF14_0770;Pf_83_83	-6.0258374	Down	5.773053	Up	-1.0148876	Down
Pfa3D7;chr10;PF10_0405;Pf_927_927	-3.7016544	Down	3.510996	Up	-1.0819823	Down
Pfa3D7;chr7;PF07_0134;Pf_896_896	-3.0479102	Down	5.407679	Up	1.6571094	Up
Pfa3D7;chr7;PF07_0133;Pf_768_768	-3.102441	Down	3.7240632	Up	1.2983562	Up
Pfa3D7;pfal_chr9;PFI0075w;Pf_839_839	-6.263824	Down	8.359712	Up	1.1377883	Up
Pfa3D7;chr7;PF07_0135;Pf_499_499	-3.4094892	Down	4.6392035	Up	1.44928	Up
Pfa3D7;pfal_chr9;PFI0020w;Pf_582_582	-8.2368355	Down	6.2291846	Up	-1.4622017	Down
Pfa3D7;chr10;PF10_0397;Pf_202_202	-2.0039814	Down	3.3587322	Up	1.5973675	Up
Pfa3D7;pfal_chr4;PFD0055w;Pf_736_736	-13.439356	Down	27.238102	Up	2.3637118	Up
Pfa3D7;chr14;PF14_0770;Pf_571_571	-7.492197	Down	7.639693	Up	-1.049223	Down
Pfa3D7;pfal_chr9;PFI0070w;Pf_853_853	-9.312627	Down	9.105055	Up	1.0414033	Up
Pfa3D7;chr6;PFF0035c;Pf_360_360	-3.8838959	Down	3.283737	Up	-1.3549132	Down
Pfa3D7;pfal_chr3;PFC0040w;Pf_782_782	-4.4414787	Down	3.0046086	Up	-1.0824434	Down
Pfa3D7;chr11;PF11_0515;Pf_774_774	-3.3118024	Down	4.5214767	Up	1.3660967	Up
Pfa3D7;pfal_chr9;PFI0065w;Pf_830_830	-5.8716435	Down	6.3255076	Up	-1.0687405	Down
Pfa3D7;chr7;PF07_0135;Pf_776_776	-4.4902077	Down	5.046313	Up	1.0320339	Up
Pfa3D7;chr10;PF10_0396;Pf_831_831	-2.0796838	Down	7.1463146	Up	3.5541472	Up
Pfa3D7;chr8;PF08_0104;Pf_763_763	-10.413141	Down	7.7950745	Up	-1.1680151	Down
Pfa3D7;pfal_chr3;PFC0010c;Pf_777_777	-5.150574	Down	4.6296043	Up	-1.0978557	Down
Pfa3D7;chr10;PF10_0396;Pf_964_964	-2.1123443	Down	7.3080554	Up	3.5127118	Up
Pfa3D7;chr14;PF14_0003;Pf_632_632	-1.4916779	Down	2.390091	Up	1.4158113	Up
Pfa3D7;chr7;PF07_0132;Pf_743_743	-9.438867	Down	11.305987	Up	1.0593544	Up
Pfa3D7;pfal_chr4;PFD0060w;Pf_469_469	-2.5050752	Down	3.480093	Up	1.3780298	Up
Pfa3D7;pfal_chr9;PFI0055c;Pf_534_534	-4.3969235	Down	4.3462653	Up	1.0972888	Up
Pfa3D7;chr14;PF14_0003;Pf_106_106	-2.1624706	Down	2.8701885	Up	1.295533	Up
Pfa3D7;chr11;PF11_0515;Pf_734_734	-3.5061195	Down	4.5741115	Up	1.1708788	Up
Pfa3D7;pfal_chr4;PFD0030c;Pf_821_821	-2.918548	Down	2.131531	Up	-1.0683634	Down
Pfa3D7;pfal_chr1;PFA0760w;Pf_855_855	-2.8663354	Down	3.1995428	Up	1.1410131	Up
Pfa3D7;chr7;PF07_0134;Pf_956_956	-2.8083768	Down	4.385767	Up	1.4025835	Up
Pfa3D7;chr14;PF14_0772;Pf_764_764	-3.8020444	Down	5.2914267	Up	1.2516409	Up
Pfa3D7;pfal_chr4;PFD1240w;Pf_764_764	-4.4437284	Down	4.847006	Up	1.1765921	Up
Pfa3D7;chr14;PF14_0005;Pf_764_764	-2.9809413	Down	3.0280752	Up	-1.0328984	Down
Pfa3D7;pfal_chr2;PFB1050w;Pf_622_622	-2.0948825	Down	2.1488364	Up	1.1990427	Up
Pfa3D7;pfal_chr9;PFI0065w;Pf_952_952	-5.0987697	Down	4.918041	Up	1.0308528	Up
Pfa3D7;chr13;MAL13P1.495;Pf_749_749	-6.743451	Down	5.944607	Up	-1.0294032	Down
Pfa3D7;pfal_chr4;PFD1020c;Pf_136_136	-5.3168855	Down	9.317782	Up	1.7030703	Up
Pfa3D7;chr13;MAL13P1.2;Pf_785_785	-1.8500974	Down	2.1848693	Up	1.1604522	Up
Pfa3D7;chr14;PF14_0769;Pf_599_599	-3.3431964	Down	6.082906	Up	1.8065101	Up
Pfa3D7;pfal_chr1;PFA0045c;Pf_862_862	-4.266775	Down	4.513426	Up	1.0104941	Up
Pfa3D7;chr10;PF10_0398;Pf_818_818	-8.367955	Down	15.077175	Up	2.200182	Up
Pfa3D7;chr10;PF10_0405;Pf_877_877	-3.4522743	Down	4.2046175	Up	1.3023064	Up
Pfa3D7;pfal_chr4;PFD1240w;Pf_625_625	-4.994949	Down	5.13337	Up	1.0353045	Up
Pfa3D7;pfal_chr1;PFA0020w;Pf_718_718	-4.451296	Down	4.7283545	Up	-1.1644986	Down
Pfa3D7;chr10;PF10_0403;Pf_888_888	1.2946079	Up	2.4035254	Up	3.855637	Up
Pfa3D7;chr11;PF11_0021;Pf_781_781	-12.068761	Down	12.842937	Up	1.2572918	Up

Accession number	[UT8] vs [UT16] Fold-change	Regulation	[UT16] vs [T16] Fold-change	Regulation	[UT8] vs [T16] Fold-change	Regulation
Pfa3D7;pfal_chr2;PFB1015w;Pf_868_868	-2.0839741	Down	3.1629043	Up	1.5663687	Up
Pfa3D7;pfal_chr1;PFA0030c;Pf_624_624	-1.691824	Down	2.3002875	Up	1.3657492	Up
Pfa3D7;chr12;PFL2615w;Pf_848_848	-2.7456536	Down	4.674256	Up	1.4940432	Up
Pfa3D7;pfal_chr4;PFD1020c;Pf_96_96	-9.408181	Down	6.4807878	Up	-1.3393394	Down
Pfa3D7;chr12;PFL2640c;Pf_788_788	-4.4310703	Down	5.4600134	Up	1.3017423	Up
Pfa3D7;chr7;PF07_0138;Pf_807_807	-4.2947173	Down	5.129685	Up	1.3369217	Up
Pfa3D7;chr7;PF07_0138;Pf_862_862	-6.505157	Down	5.2364364	Up	-1.125194	Down
Pfa3D7;pfal_chr3;PFC0040w;Pf_628_628	-2.9313574	Down	3.6823769	Up	1.1128387	Up
Pfa3D7;chr12;PFL2640c;Pf_748_748	-13.397569	Down	16.048334	Up	1.3943334	Up
Pfa3D7;pfal_chr1;PFA0045c;Pf_729_729	-3.2651544	Down	4.5850153	Up	1.4429605	Up
Pfa3D7;chr11;PF11_0011;Pf_86_86	-2.2709432	Down	2.8783648	Up	1.3542016	Up
Pfa3D7;pfal_chr4;PFD1225w;Pf_64_64	-5.2140703	Down	4.6890564	Up	-1.4279703	Down
Pfa3D7;chr6;PFF0040c;Pf_673_673	-7.638709	Down	8.442142	Up	1.0492177	Up
Pfa3D7;pfal_chr4;PFD0120w;Pf_778_778	-1.1152478	Down	-2.6842957	Down	-4.321766	Down
Pfa3D7;pfal_chr5;PFE1635w;Pf_831_831	-1.9604808	Down	3.9714944	Up	2.022031	Up
Pfa3D7;pfal_chr4;PFD1225w;Pf_104_104	-5.185873	Down	6.9099455	Up	1.2416732	Up
Pfa3D7;chr13;MAL13P1.8;Pf_693_693	-4.331346	Down	4.3782063	Up	1.0320613	Up
Pfa3D7;pfal_chr5;PFE1635w;Pf_951_951	-1.5850785	Down	3.1591222	Up	1.9290181	Up
Pfa3D7;pfal_chr4;PFD0765w;Pf_2569_2569	2.0157192	Up	-1.9803231	Down	1.0555679	Up
Pfa3D7;pfal_chr9;PFI1755c;Pf_869_869	2.2528381	Up	-1.6191436	Down	1.2861336	Up
Pfa3D7;chr11;PF11_0512;Pf_2345_2345	3.3351743	Up	-1.812522	Down	1.7142093	Up
Pfa3D7;chr14;PF14_0096;Pf_1256_1256	-2.524426	Down	2.1981823	Up	-1.0783759	Down
Pfa3D7;chr14;PF14_0096;Pf_1296_1296	-2.4547374	Down	2.0442166	Up	-1.1395025	Down
Pfa3D7;chr10;PF10_0047;Pf_2541_2541	-3.5190098	Down	2.7478054	Up	-1.3632778	Down
Pfa3D7;chr6;PFF1425w;Pf_1461_1461	-1.9524829	Down	2.1220808	Up	1.0247884	Up
Pfa3D7;chr6;PFF1425w;Pf_1833_1833	-1.8412819	Down	2.0650942	Up	1.1332085	Up
Pfa3D7;pfal_chr4;PFD0775c;Pf_1629_1629	-2.3113267	Down	2.190174	Up	-1.0102129	Down
Pfa3D7;chr10;PF10_0047;Pf_2501_2501	-3.3242738	Down	2.6433148	Up	-1.2952927	Down
Pfa3D7;pfal_chr4;PFD0775c;Pf_1589_1589	-2.281434	Down	2.159493	Up	-1.0480471	Down
Pfa3D7;chr11;PF11_0058;Pf_615_615	-2.5856175	Down	1.7926904	Up	-1.3758533	Down
Pfa3D7;chr11;PF11_0058;Pf_574_574	-2.3083715	Down	1.8405365	Up	-1.2192428	Down
Pfa3D7;chr14;PF14_0151;Pf_865_865	-3.911389	Down	2.595744	Up	-1.4505253	Down
Pfa3D7;chr14;PF14_0151;Pf_824_824	-3.7184272	Down	2.7368345	Up	-1.3268518	Down
Pfa3D7;chr11;PF11_0320;Pf_383_383	-2.2168112	Down	2.0894551	Up	-1.0070713	Down
Pfa3D7;chr8;PF08_0100;Pf_1183_1183	-1.2273576	Down	-2.0493126	Down	-2.2616284	Down
Pfa3D7;chr8;MAL8P1.41;Pf_563_563	1.3316814	Up	1.6787685	Up	2.158088	Up
Pfa3D7;chr12;PFL1775c;Pf_1440_1440	-3.8075407	Down	2.9114528	Up	-1.2394061	Down
Pfa3D7;chr13;PF13_0324;Pf_2769_2769	2.3224902	Up	-2.0360155	Down	1.1170595	Up
Pfa3D7;chr14;PF14_0040;Pf_502_502	1.3004612	Up	-3.3723798	Down	-2.4117029	Down
Pfa3D7;chr14;PF14_0040;Pf_542_542	1.5164347	Up	-3.5737467	Down	-2.2817967	Down
Pfa3D7;pfal_chr9;PFI0505c;Pf_3370_3370	-1.6192021	Down	2.213	Up	1.3184365	Up
Pfa3D7;pfal_chr9;PFI0505c;Pf_3514_3514	-1.8884859	Down	2.487249	Up	1.3568404	Up
Pfa3D7;pfal_chr2;PFB0360c;Pf_2810_2810	1.6678938	Up	-2.0407705	Down	-1.1607244	Down
Pfa3D7;pfal_chr2;PFB0325c;Pf_1857_1857	1.7242345	Up	-10.95894	Down	-8.417901	Down
Pfa3D7;pfal_chr4;PFD1175w;Pf_3388_3388	-1.1209186	Down	-1.7363344	Down	-2.036624	Down
Pfa3D7;chr12;PFL0040c;Pf_1498_1498	2.3860588	Up	-2.0920928	Down	1.1281767	Up
Pfa3D7;pfal_chr9;PFI0115c;Pf_1414_1414	2.2604222	Up	-1.8949379	Down	1.0601721	Up
Pfa3D7;chr12;PFL0040c;Pf_1543_1543	2.558793	Up	-2.2264278	Down	1.119438	Up
Pfa3D7;pfal_chr9;PFI0095c;Pf_1470_1470	2.5136273	Up	-1.45986	Down	1.6064113	Up
Pfa3D7;chr11;PF11_0510;Pf_1568_1568	3.2427573	Up	-1.6699113	Down	1.9846011	Up
Pfa3D7;pfal_chr9;PFI0120c;Pf_1682_1682	4.481132	Up	-2.061685	Down	2.055258	Up
Pfa3D7;chr11;PF11_0510;Pf_1429_1429	3.2852337	Up	-1.6786206	Down	1.9365202	Up
Pfa3D7;pfal_chr3;PFC0060c;Pf_1686_1686	7.5721736	Up	-2.9638202	Down	2.3644474	Up
Pfa3D7;pfal_chr4;PFD1175w;Pf_3595_3595	-1.175264	Down	-1.8696532	Down	-2.2913828	Down
Pfa3D7;pfal_chr3;PFC0060c;Pf_1536_1536	7.0810585	Up	-3.5128138	Down	1.9693382	Up
Pfa3D7;pfal_chr9;PFI0100c;Pf_1448_1448	2.4993737	Up	-1.9685473	Down	1.198888	Up

	[UT8] vs [UT16]		[UT16] vs [T16]		[UT8] vs [T16]	
**Accession number**	**Fold-change**	**Regulation**	**Fold-change**	**Regulation**	**Fold-change**	**Regulation**
Pfa3D7;chr14;PF14_0227;Pf_852_852	-2.597834	Down	1.3594087	Up	-1.8159928	Down
Pfa3D7;chr8;PF08_0129;Pf_1695_1695	1.2700531	Up	-2.0193372	Down	-1.463034	Down
Pfa3D7;chr11;PF11_0321;Pf_2007_2007	-4.9628057	Down	2.0653026	Up	-1.8526917	Down
Pfa3D7;chr11;PF11_0321;Pf_2163_2163	-5.8281226	Down	2.0001225	Up	-2.812568	Down
Pfa3D7;pfal_chr4;PFD0190w;Pf_3431_3431	1.9460539	Up	-2.1576195	Down	-1.1549175	Down
Pfa3D7;pfal_chr4;PFD0310w;Pf_153_153	-1.2211138	Down	-1.85125	Down	-2.3915393	Down
Pfa3D7;chr12;PFL2245w;Pf_694_694	-3.422214	Down	2.4156506	Up	-1.3624465	Down
Pfa3D7;pfal_chr5;PFE0435c;Pf_794_794	-3.7104027	Down	3.113011	Up	-1.2687868	Down
Pfa3D7;chr8;MAL8P1.78;Pf_1_1	-1.7246023	Down	-2.670083	Down	-3.5411582	Down
Pfa3D7;chr8;MAL8P1.78;Pf_262_262	-1.6950119	Down	-2.4065447	Down	-3.6584044	Down
Pfa3D7;pfal_chr5;PFE0285c;Pf_238_238	-1.6381838	Down	2.1740818	Up	1.3830495	Up
Pfa3D7;chr8;MAL8P1.21;Pf_640_640	-3.0429952	Down	2.3958676	Up	-1.2775055	Down
Pfa3D7;chr6;PFF1025c;Pf_785_785	-2.0969098	Down	1.7185557	Up	-1.1865449	Down
Pfa3D7;chr6;PFF1025c;Pf_667_667	-2.22733	Down	1.4502485	Up	-1.5116383	Down
Pfa3D7;chr13;MAL13P1.206;Pf_1984_1984	1.6275814	Up	-2.0879567	Down	-1.2773701	Down
Pfa3D7;chr14;PF14_0493;Pf_2150_2150	-2.8829641	Down	1.2370274	Up	-2.2141263	Down
Pfa3D7;pfal_chr3;PFC0165w;Pf_6867_6867	-3.2763276	Down	2.8551793	Up	-1.0744545	Down
Pfa3D7;chr11;PF11_0480;Pf_8461_8461	1.8063785	Up	-4.312257	Down	-2.5480268	Down
Pfa3D7;pfal_chr4;PFD0425w;Pf_2717_2717	1.4117054	Up	-2.3682816	Down	-1.6930009	Down
Pfa3D7;pfal_chr4;PFD0425w;Pf_2619_2619	1.353934	Up	-2.2549248	Down	-1.7121291	Down
Pfa3D7;chr13;PF13_0201;Pf_1650_1650	-1.1525694	Down	-2.6691308	Down	-2.9927313	Down
Pfa3D7;chr13;PF13_0201;Pf_1364_1364	-1.3239589	Down	-2.326111	Down	-2.972547	Down
Pfa3D7;pfal_chr4;PFD1035w;Pf_825_825	-2.144371	Down	2.2934198	Up	-1.0076038	Down
Pfa3D7;pfal_chr4;PFD1035w;Pf_904_904	-2.0674994	Down	2.2045813	Up	-1.0081872	Down
Pfa3D7;pfal_chr3;PFC0025c;Pf_699_699	-2.4567604	Down	2.66338	Up	1.1679615	Up
Pfa3D7;chr14;PF14_0771;Pf_676_676	-2.9961348	Down	3.6613564	Up	1.4896494	Up
Pfa3D7;chr13;MAL13P1.7;Pf_697_697	-7.50286	Down	10.504428	Up	1.3702246	Up
Pfa3D7;pfal_chr4;PFD1220c;Pf_538_538	-13.550726	Down	23.112585	Up	2.1218543	Up
Pfa3D7;pfal_chr1;PFA0750w;Pf_678_678	-4.9754696	Down	9.8282995	Up	1.8578887	Up
Pfa3D7;chr6;PFF1550w;Pf_665_665	-10.04054	Down	18.997358	Up	1.9490459	Up
Pfa3D7;chr13;MAL13P1.490;Pf_577_577	-12.5089035	Down	18.420258	Up	1.5240763	Up
Pfa3D7;chr6;PFF0850c;Pf_692_692	-9.031766	Down	25.403711	Up	3.0966306	Up
Pfa3D7;chr13;MAL13P1.490;Pf_537_537	-13.572703	Down	21.744726	Up	1.7837807	Up
Pfa3D7;chr14;PF14_0007;Pf_692_692	-5.3236704	Down	7.466056	Up	1.3180206	Up
Pfa3D7;chr10;PF10_0395;Pf_759_759	-15.774636	Down	62.8726	Up	4.7900825	Up
Pfa3D7;chr13;MAL13P1.7;Pf_609_609	-7.556169	Down	11.527165	Up	1.3239621	Up
Pfa3D7;pfal_chr4;PFD1220c;Pf_634_634	-12.495864	Down	22.335556	Up	2.2654855	Up
Pfa3D7;chr12;PFL2610w;Pf_588_588	-1.7109298	Down	3.525811	Up	1.9086733	Up
Pfa3D7;chr12;PFL2610w;Pf_648_648	-1.675805	Down	2.3537374	Up	1.4647849	Up
Pfa3D7;chr10;PF10_0395;Pf_719_719	-16.983034	Down	49.57544	Up	3.0020816	Up
Pfa3D7;chr6;PFF1550w;Pf_623_623	-10.049598	Down	14.385746	Up	1.6836971	Up
Pfa3D7;chr6;PFF0850c;Pf_613_613	-8.083183	Down	15.967463	Up	2.1505296	Up
Pfa3D7;chr14;PF14_0007;Pf_618_618	-3.7965212	Down	5.7805014	Up	1.9196258	Up
Pfa3D7;chr12;PFL2635w;Pf_348_348	-4.9598346	Down	5.007126	Up	-1.1365215	Down
Pfa3D7;chr14;PF14_0767;Pf_587_587	-7.8142085	Down	12.007656	Up	1.6173648	Up
Pfa3D7;chr14;PF14_0771;Pf_571_571	-2.7229538	Down	3.8072798	Up	1.4593463	Up
Pfa3D7;chr11;PF11_0516;Pf_407_407	-7.367809	Down	7.5457945	Up	1.110596	Up
Pfa3D7;pfal_chr1;PFA0750w;Pf_638_638	-10.3709	Down	14.274201	Up	1.5711033	Up
Pfa3D7;chr13;MAL13P1.505;Pf_670_670	-1.9981116	Down	2.871446	Up	1.4003931	Up
Pfa3D7;chr12;PFL2620w;Pf_375_375	-3.7968915	Down	3.9388452	Up	-1.0884243	Down
Pfa3D7;chr11;PF11_0516;Pf_570_570	-7.1105967	Down	11.537958	Up	1.7333858	Up
Pfa3D7;pfal_chr4;PFD0035c;Pf_734_734	-1.9003583	Down	2.0672534	Up	1.1462479	Up
Pfa3D7;chr14;PF14_0767;Pf_695_695	-2.4729314	Down	3.8231633	Up	1.6964124	Up
Pfa3D7;pfal_chr9;PFI0045c;Pf_531_531	-1.927499	Down	2.5478752	Up	1.4128741	Up
Pfa3D7;chr7;PF07_0130;Pf_598_598	-2.1098912	Down	1.8119774	Up	1.0939581	Up

	[UT8] vs [UT16]		[UT16] vs [T16]		[UT8] vs [T16]	
**Accession number**	**Fold-change**	**Regulation**	**Fold-change**	**Regulation**	**Fold-change**	**Regulation**
Pfa3D7;chr7;PF07_0130;Pf_697_697	-2.646605	Down	2.7974045	Up	1.1876702	Up
Pfa3D7;pfal_chr3;PFC1105w;Pf_563_563	-7.3235245	Down	8.388672	Up	1.2704353	Up
Pfa3D7;pfal_chr3;PFC1105w;Pf_663_663	-10.103441	Down	13.376127	Up	1.1400242	Up
Pfa3D7;pfal_chr4;PFD0065w;Pf_896_896	-2.1958652	Down	2.801209	Up	1.377489	Up
Pfa3D7;chr11;PF11_0013;Pf_524_524	-4.398066	Down	4.5395985	Up	-1.0289284	Down
Pfa3D7;pfal_chr4;PFD0065w;Pf_711_711	-1.7814031	Down	2.854486	Up	1.7107567	Up
Pfa3D7;chr11;PF11_0013;Pf_569_569	-5.323786	Down	5.6944823	Up	1.0845532	Up
Pfa3D7;chr10;PF10_0009;Pf_573_573	-4.4838195	Down	8.72424	Up	1.7665944	Up
Pfa3D7;chr10;PF10_0009;Pf_700_700	-6.6395316	Down	9.398292	Up	1.1460778	Up
Pfa3D7;chr14;PF14_0382;Pf_4491_4491	-3.2469501	Down	2.824223	Up	-1.3129134	Down
Pfa3D7;pfal_chr1;PFA0650w;Pf_2330_2330	7.6314654	Up	-3.4156435	Down	2.0293245	Up
Pfa3D7;pfal_chr1;PFA0650w;Pf_2390_2390	8.440719	Up	-3.449857	Down	2.2353845	Up
Pfa3D7;pfal_chr1;PFA0655w;Pf_2441_2441	9.328502	Up	-2.7795923	Down	2.7345047	Up
Pfa3D7;chr13;PF13_0074;Pf_5250_5250	8.288827	Up	-5.0894294	Down	1.4350828	Up
Pfa3D7;chr13;PF13_0074;Pf_5210_5210	8.097024	Up	-4.6506543	Down	1.600625	Up
Pfa3D7;pfal_chr4;PFD0105c;Pf_2277_2277	4.8951197	Up	-1.4443665	Down	3.2285001	Up
Pfa3D7;pfal_chr4;PFD0105c;Pf_2229_2229	4.84807	Up	-1.42452	Down	3.2451706	Up
Pfa3D7;pfal_chr4;PFD1160w;Pf_7039_7039	4.538997	Up	-1.8926142	Down	2.0873902	Up
Pfa3D7;chr8;MAL8P1.1;Pf_2858_2858	-3.302323	Down	2.9246824	Up	1.0298592	Up
Pfa3D7;chr12;PFL0505c;Pf_818_818	-2.296601	Down	1.8088738	Up	-1.1626688	Down
Pfa3D7;pfal_chr9;PFI0790w;Pf_313_313	2.0268188	Up	-1.6220852	Down	1.2142407	Up
Pfa3D7;chr14;PF14_0545;Pf_239_239	-1.6479403	Down	2.885146	Up	1.8290467	Up
Pfa3D7;chr7;MAL7P1.88;Pf_1253_1253	-2.051615	Down	1.741798	Up	-1.1794411	Down
Pfa3D7;chr12;PFL2465c;Pf_497_497	1.430005	Up	-2.1623647	Down	-1.46863	Down
Pfa3D7;chr12;PFL0430w;Pf_98_98	-2.9127965	Down	2.6594305	Up	-1.0690103	Down
Pfa3D7;pfal_chr9;PFI0970c;Pf_3178_3178	2.4203947	Up	-1.8291525	Down	1.2492129	Up
Pfa3D7;chr10;PF10_0075;Pf_4593_4593	-4.1857924	Down	2.5058014	Up	-1.8437892	Down
Pfa3D7;chr11;PF11_0091;Pf_5337_5337	-6.390821	Down	1.3982557	Up	-4.118069	Down
Pfa3D7;chr13;PF13_0097;Pf_7815_7815	-6.1187477	Down	2.3074286	Up	-2.4615989	Down
Pfa3D7;chr7;PF07_0126;Pf_3888_3888	2.4891737	Up	-1.6497486	Down	1.5475844	Up
Pfa3D7;chr7;PF07_0126;Pf_3828_3828	2.5682476	Up	-1.6337366	Down	1.5950463	Up
Pfa3D7;chr6;PFF0200c;Pf_5517_5517	2.0787826	Up	-2.057131	Down	1.1238402	Up
Pfa3D7;pfal_chr4;PFD0360w;Pf_812_812	2.2028182	Up	-1.6958923	Down	1.135398	Up
Pfa3D7;pfal_chr5;PFE0305w;Pf_907_907	2.2212696	Up	-1.7620292	Down	1.2029546	Up
Pfa3D7;pfal_chr5;PFE0305w;Pf_767_767	2.3168187	Up	-1.6759226	Down	1.2735348	Up
Pfa3D7;pfal_chr5;PFE0870w;Pf_3279_3279	-2.014028	Down	1.8341657	Up	-1.1605897	Down
Pfa3D7;pfal_chr5;PFE0870w;Pf_3237_3237	-2.0155933	Down	1.8384188	Up	-1.1492131	Down
Pfa3D7;chr14;PF14_0344;Pf_2735_2735	-1.0661632	Down	-1.8550837	Down	-2.0118642	Down
Pfa3D7;pfal_chr9;PFI0720w;Pf_1461_1461	2.2250104	Up	-2.3410146	Down	-1.0197326	Down
Pfa3D7;pfal_chr5;PFE1185w;Pf_1856_1856	2.506407	Up	-2.5901299	Down	-1.0578626	Down
Pfa3D7;pfal_chr9;PFI0720w;Pf_1421_1421	2.1049178	Up	-2.1466305	Down	1.0079434	Up
Pfa3D7;chr11;PF11_0310;Pf_1769_1769	-2.4167647	Down	1.4543699	Up	-1.6089525	Down
Pfa3D7;pfal_chr5;PFE1185w;Pf_1761_1761	2.4876542	Up	-2.5239413	Down	-1.02886	Down
Pfa3D7;chr11;PF11_0310;Pf_1621_1621	-2.4427683	Down	1.5126209	Up	-1.5249596	Down
Pfa3D7;pfal_chr5;PFE0410w;Pf_957_957	-1.8750066	Down	2.0817013	Up	1.1229632	Up
Pfa3D7;pfal_chr5;PFE0410w;Pf_369_369	-2.0195162	Down	2.2057207	Up	1.1203772	Up
Pfa3D7;pfal_chr3;PFC0831w;Pf_954_954	1.1176881	Up	-2.8084404	Down	-2.4474845	Down
Pfa3D7;chr14;PF14_0378;Pf_511_511	-1.0609324	Down	2.0168357	Up	1.7708415	Up
Pfa3D7;chr14;PF14_0378;Pf_168_168	-1.0199747	Down	2.068132	Up	1.864608	Up
Pfa3D7;chr11;PF11_0198;Pf_667_667	-2.1541302	Down	1.8692267	Up	-1.1221886	Down
Pfa3D7;chr8;PF08_0003;Pf_1957_1957	5.7390428	Up	-2.185284	Down	2.5527236	Up
Pfa3D7;chr8;PF08_0003;Pf_1855_1855	5.9343395	Up	-2.8531604	Down	2.021776	Up
Pfa3D7;chr14;PF14_0725;Pf_1427_1427	1.008558	Up	-2.6063986	Down	-2.7903197	Down
Pfa3D7;chr14;PF14_0502;Pf_944_944	-1.7159652	Down	2.08643	Up	1.1747334	Up
Pfa3D7;chr14;PF14_0502;Pf_484_484	-1.9494838	Down	2.3418922	Up	1.1928395	Up
Pfa3D7;chr8;MAL8P1.9;Pf_161_161	-1.679611	Down	2.256581	Up	1.3080281	Up

	[UT8] vs [UT16]		[UT16] vs [T16]		[UT8] vs [T16]	
**Accession number**	**Fold-change**	**Regulation**	**Fold-change**	**Regulation**	**Fold-change**	**Regulation**
Pfa3D7;chr13;MAL13P1.227;Pf_613_613	-2.1839547	Down	1.7787642	Up	-1.2406946	Down
Pfa3D7;chr12;PFL2175w;Pf_336_336	-1.3941178	Down	2.6209989	Up	1.8797537	Up
Pfa3D7;pfal_chr9;PFI1030c;Pf_376_376	-1.8156888	Down	2.1615694	Up	1.2325306	Up
Pfa3D7;chr11;PF11_0393;Pf_182_182	2.793835	Up	-2.0582697	Down	1.3692751	Up
Pfa3D7;pfal_chr4;PFD0165w;Pf_1139_1139	-2.0140738	Down	1.9852178	Up	-1.0963767	Down
Pfa3D7;chr13;MAL13P1.64;Pf_135_135	-2.032307	Down	1.87068	Up	-1.0287813	Down
Pfa3D7;chr13;PF13_0084;Pf_474_474	2.526586	Up	-3.6455228	Down	-1.6630652	Down
Pfa3D7;chr13;PF13_0084;Pf_434_434	2.423914	Up	-4.25215	Down	-2.0973957	Down
Pfa3D7;pfal_chr3;PFC0550w;Pf_1759_1759	-3.2798235	Down	2.0874512	Up	-1.4255887	Down
Pfa3D7;pfal_chr3;PFC0550w;Pf_1799_1799	-3.2252707	Down	2.17476	Up	-1.5068218	Down
Pfa3D7;chr14;PF14_0462;Pf_2336_2336	-1.9267459	Down	2.3693743	Up	1.2015934	Up
Pfa3D7;chr14;PF14_0462;Pf_2416_2416	-1.8328427	Down	2.4044762	Up	1.3254657	Up
Pfa3D7;chr11;PF11_0385;Pf_1208_1208	-2.5362995	Down	1.8896244	Up	-1.3112448	Down
Pfa3D7;chr11;PF11_0385;Pf_1087_1087	-2.42557	Down	2.0008216	Up	-1.1969957	Down
Pfa3D7;chr10;PF10_0261;Pf_1715_1715	1.0706543	Up	-2.0583813	Down	-1.7709906	Down
Pfa3D7;chr14;PF14_0608;Pf_1991_1991	2.7068746	Up	-1.936532	Down	1.333354	Up
Pfa3D7;chr14;PF14_0608;Pf_1891_1891	2.3995376	Up	-2.0638735	Down	1.1267214	Up
Pfa3D7;chr12;PFL0440c;Pf_3427_3427	1.5850893	Up	-2.0238695	Down	-1.2931726	Down
Pfa3D7;chr6;PFF0485c;Pf_712_712	2.0349562	Up	-2.1232812	Down	1.0669193	Up
Pfa3D7;chr14;PF14_0610;Pf_951_951	2.3622766	Up	-1.9660877	Down	1.125255	Up
Pfa3D7;chr6;PFF0485c;Pf_752_752	2.0604258	Up	-2.0535142	Down	1.0935987	Up
Pfa3D7;chr12;PFL0465c;Pf_4313_4313	-2.0562124	Down	1.5615336	Up	-1.3967856	Down
Pfa3D7;chr10;PF10_0216;Pf_615_615	1.0352788	Up	-2.0014305	Down	-1.9074595	Down
Pfa3D7;pfal_chr1;PFA0170c;Pf_4661_4661	-2.2696257	Down	1.7648969	Up	-1.2113625	Down
Pfa3D7;pfal_chr4;PFD0015c;Pf_801_801	-2.6595805	Down	3.0704975	Up	1.1559079	Up
Pfa3D7;pfal_chr4;PFD0015c;Pf_721_721	-2.8016038	Down	3.2891905	Up	1.2740954	Up
