# Supplementary material for: In vitro antiplasmodial activity of cepharanthine
Source: Malar J. 2014 Aug 22;13:327. doi: 10.1186/1475-2875-13-327 (PMC4152577; doi:10.1186/1475-2875-13-327)
Supplement: Supplementary file 3 — Additional file 3: Hierarchical classification of Gene Ontology terms enriched during the transcriptomic experiment. (PDF 943 KB) [file 12936_2014_3366_MOESM3_ESM.pdf]

Additional file 3: Hierarchical classification of Gene Ontology terms enriched during the transcriptomic experiment.

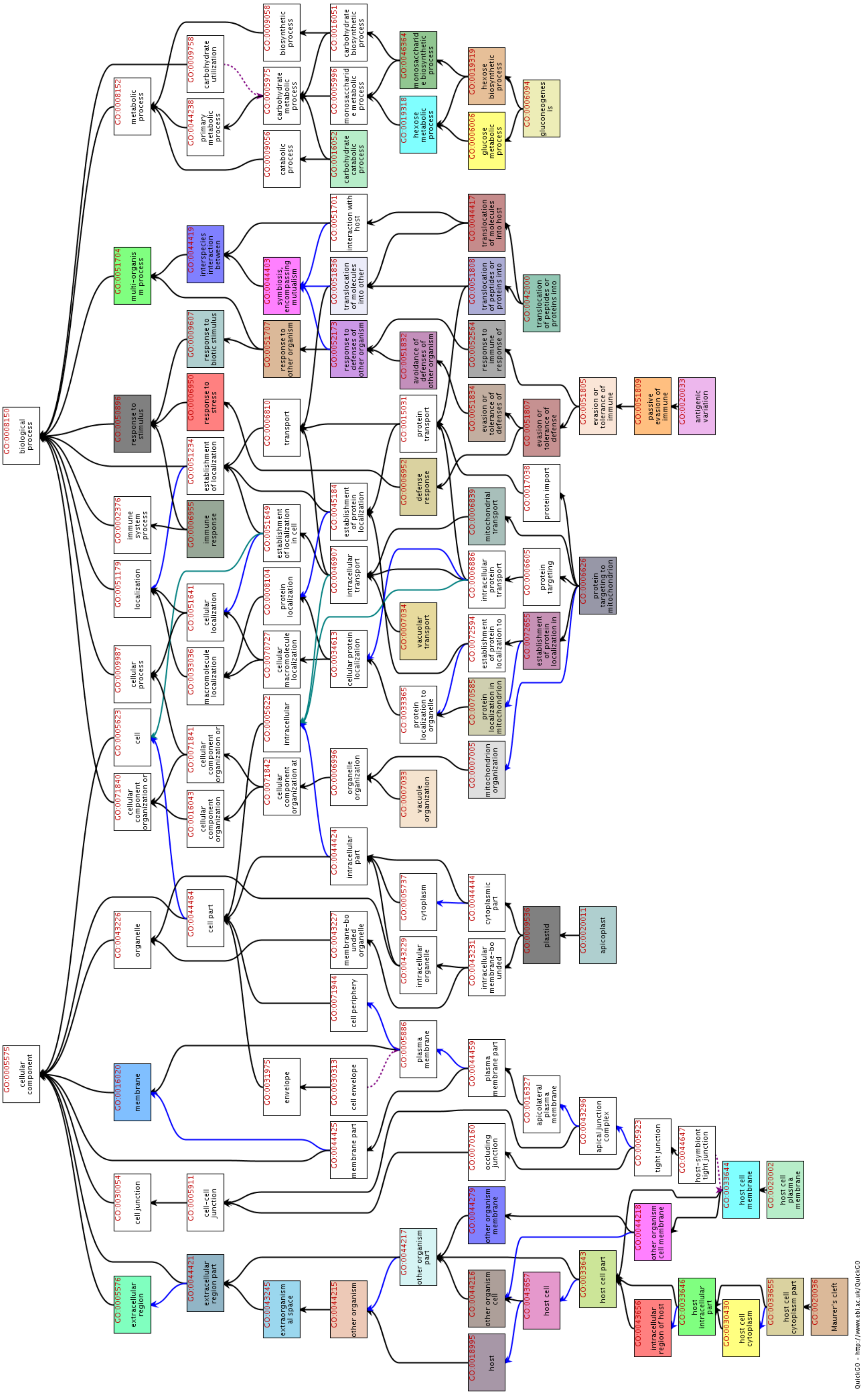

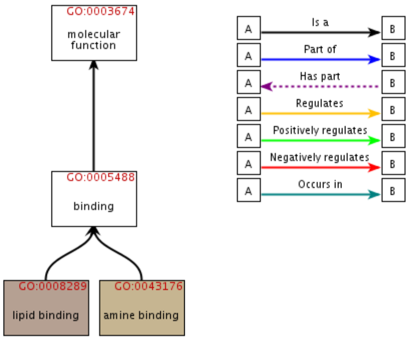

QuickGO was used to produce this Ancestral Chart representing significantly overrepresented GOs. This graph is divided into three groups: biological process, cellular component and molecular function.
