# Supplementary material for: In vitro antiplasmodial activity of cepharanthine
Source: Malar J. 2014 Aug 22;13:327. doi: 10.1186/1475-2875-13-327 (PMC4152577; doi:10.1186/1475-2875-13-327)
Supplement: Supplementary file 4 — Additional file 4: Differences of expression in pathways due to cepharanthine. These three histograms compare the expression of pathways by pairs of experimental conditions. Horizontal bars lengths correspond to the number of genes up-regulated (red) and down-regulated (green) from each metabolic pathway. FC = fold change, UT = untreated, T = treated. (PDF 92 KB) [file 12936_2014_3366_MOESM4_ESM.pdf]

Additional file 4: Differences of expression in pathways due to cepharanthine.

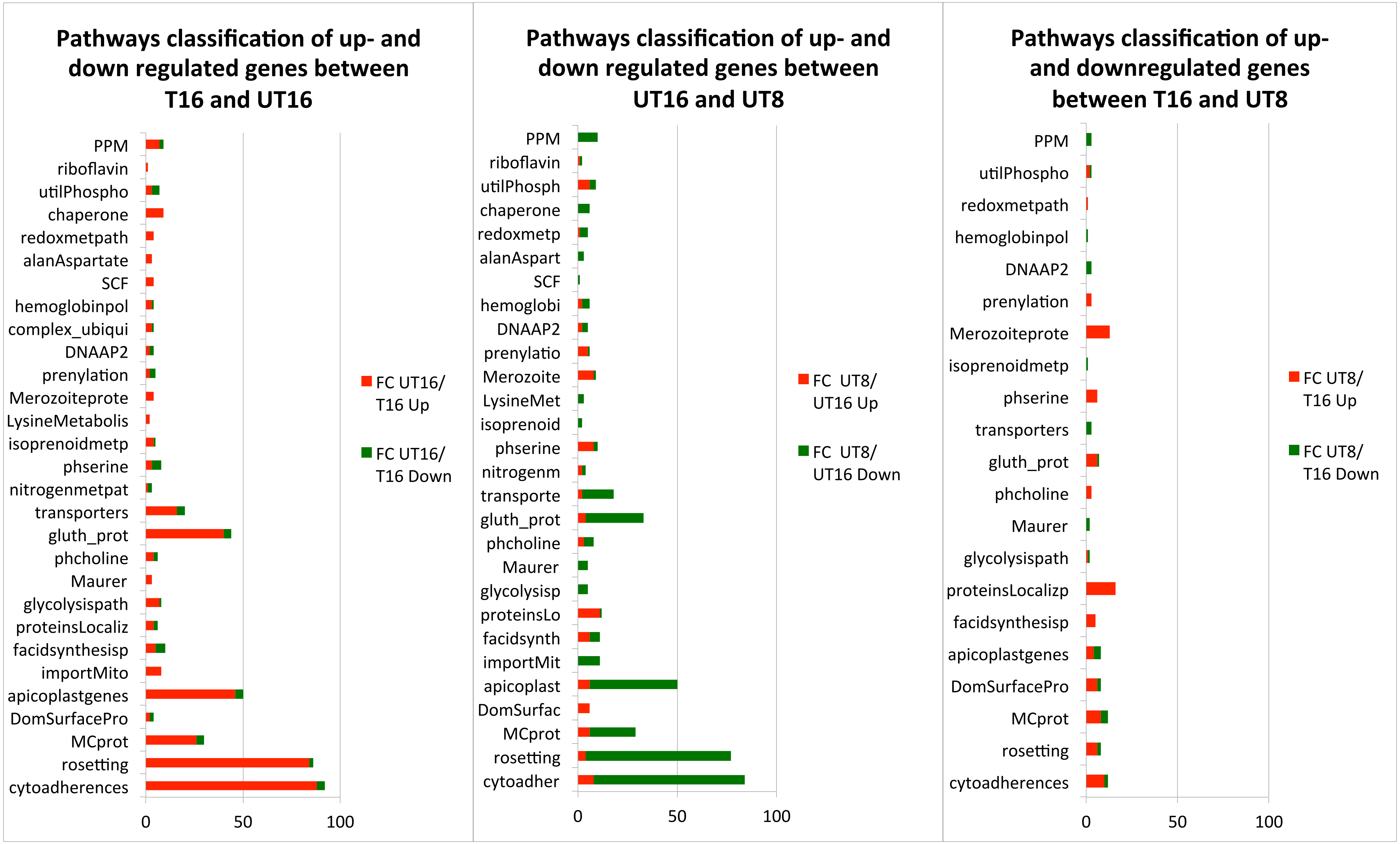

These three histograms compare the expression of pathways by pairs of experimental conditions. Horizontal bars lengths correspond to the number of genes up-regulated (red and down-regulated (green) from each metabolic pathway. FC = foldchange, UT = untreated, T = treated.
