# Supplementary material for: In vitro antiplasmodial activity of cepharanthine
Source: Malar J. 2014 Aug 22;13:327. doi: 10.1186/1475-2875-13-327 (PMC4152577; doi:10.1186/1475-2875-13-327)
Supplement: Supplementary file 5 — Additional file 5: Metabolic pathways modified by cepharanthine. Over-represented pathways were grouped into functions according to the Malaria Parasite Metabolic Pathways database. P-values were obtained by Fisher exact t-tests. The threshold of significance is 0.05. (PDF 102 KB) [file 12936_2014_3366_MOESM5_ESM.pdf]

Additional file 5 : Metabolic pathways modified by cepharanthine.

Function	Metabolic Pathway	Complet name of the Pathway	Number of pathway's gene in the selection	Total number of genes in the pathway	Number of genes in the selection	Number of genes in PlasmoDB	P-value Fisher exact t-test right tail
Amino Acid	LysineMetabolism	Lysine metabolism	3	6	781	5685	0.038
Amino Acid	alanAspartate	Asparagine and Aspartate Metabolism	3	8	781	5685	0.085
Amino Acid	nitrogenmetpath	Nitrogen Metabolism	4	9	781	5685	0.025
Carbohydrate	glycolysispath	Glycolysis	9	28	781	5685	0.010
Cell-Cell interaction	cytoadherencescheme	Cytoadherence: constitutive and inducible receptors	99	333	781	5685	2.49E-013
Cell-Cell interaction	rosetting	Cytoadherence: Rosette formation between normal and infected RBC	91	317	781	5685	1.54E-011
Cofactors & other substances	riboflavin	Riboflavin metabolism	2	4	781	5685	0.094
Hemoglobine digestion	hemoglobinpolpath	Hemoglobin Digestion & Ferriprotoporphyrin IX Polymerization	7	26	781	5685	0.057
Invasion & motility	DomSurfaceProt	Domains of merozoite surface proteins	9	14	781	5685	1.88E-005
Invasion & motility	proteinsLocalizpath	Subcellular localization of proteins involved in invasion	19	79	781	5685	0.010
Invasion & motility	Merozoiteproteins	Functional annotation of merozoite invasion-related proteins	16	74	781	5685	0.043
Lipids	phcholine	Phosphatidylcholine metabolism	8	24	781	5685	0.012
Lipids	phserine	Phosphatidylethanolamine and phosphatidylserine metabolism	12	48	781	5685	0.027
Lipids	isoprenoidmetpath	Isoprenoids Metabolism	5	14	781	5685	0.034
Lipids	utilPhospho	Utilization of phospholipids	10	46	781	5685	0.093
Organellar function	apicoplastgenes	Nuclear genes with apicoplast signal sequences	63	281	781	5685	8.38E-005
Organellar function	importMito	Import of proteins into the mitochondrion	11	24	781	5685	1.52E-004
Organellar function	chaperone	Chaperone network and protein quality control of the mitochondrion	9	40	781	5685	0.091
Organellar function. Lipids	facidsynthesispath	Fatty acid synthesis in the apicoplast	13	40	781	5685	0.002
Post translational modifications	gluth_prot	S-Glutathionylated proteins	58	318	781	5685	0.017
Post translational modifications	prenylation	N-myristoylation. S-acylation and prenylation of proteins	7	25	781	5685	0.047
Post translational modifications	complex_ubiquitin_ligase	Anaphase promoting complex ubiquitin-ligase	4	11	781	5685	0.053
Post translational modifications	SCF	SCF (Skp1-Cullin-F-box) ubiquitin-ligase	5	16	781	5685	0.058
Protein traffic	MCprot	Established and putative Maurer's clefts proteins	38	109	781	5685	3.29E-008
Protein traffic	Maurer	Exported parasite proteins associated with Maurer's clefts	5	11	781	5685	0.011
Redox metabolism	redoxmetpath	Redox Metabolism	8	34	781	5685	0.087
Transcription	DNAAP2	DNA binding proteins with AP2 domain(s)	6	20	781	5685	0.047
Transport	transporters	Genes coding for transport proteins	25	120	781	5685	0.022
Transport	PPM	Transporters of the plasma membrane	13	64	781	5685	0.095

Over-represented pathways were annotated/grouped into functions according to the Malaria Parasite Metabolic Pathways database. P-values were obtained by Fisher exact t-tests. The threshold of significance is 0.05.
